# Therapeutic modulation of the vitreoretinal fibrosis microenvironment in female mice using engineered macrophage-derived extracellular vesicles

**DOI:** 10.1038/s41467-026-76550-z

**Published:** 2026-08-25

**Authors:** Yingjie Wang, Fuxiao Luan, Jiawei Zhao, Peilin Guo, Shuang Wang, Jinghui Wang, Dongfang Wang, Jing Hou, Xiaofeng Hu, Li Chen, Xusheng Cao, Guanghui Ma, Yong Tao, Ying Tian, Wei Wei

**Affiliations:** 1 Department of Ophthalmology, Beijing Chaoyang Hospital, Capital Medical University, Beijing, PR China; 2 State Key Laboratory of Biopharmaceutical Preparation and Delivery, Institute of Process Engineering, Chinese Academy of Sciences, Beijing, PR China; 3 School of Chemical Engineering, University of Chinese Academy of Sciences, Beijing, PR China; 4 Department of Gastroenterology, Tongren Hospital, Shanghai Jiao Tong University School of Medicine, Shanghai, PR China; 5 Biomedical Pioneering Innovation Center (BIOPIC), Peking University, Beijing, PR China; 6 Department of Ophthalmology, Peking University People’s Hospital, Beijing, PR China; 7 Beijing Key Laboratory of Ocular Disease and Optometry Science, Peking University People’s Hospital, Beijing, PR China; 8 Beijing Key Laboratory of Ophthalmology and Visual Sciences, Beijing Tongren Eye Center, Beijing Tongren Hospital, Capital Medical University, Beijing, PR China; 9 National Engineering Research Center for Ophthalmology, Beijing, PR China; 10 Engineering Research Center of Ophthalmic Equipment and Materials, Ministry of Education, Beijing, PR China; 11 Chinese Institutes for Medical Research, Beijing, PR China

**Keywords:** Retinal diseases, Drug delivery

## Abstract

Vitreoretinal fibrosis, a hallmark of proliferative vitreoretinopathy (PVR) triggered by retinal detachment or ocular trauma, necessitates surgery. Through data mining of the vitreoretinal fibrosis microenvironment in PVR patients and mice, showing elevated transforming growth factor β1 (TGFβ1) and M2 macrophage enrichment, we designed and engineered extracellular vesicles that conferred anti-fibrotic efficacy against PVR. These M1 macrophage-derived vesicles (M1evs) were conjugated with anti-TGFβ1 antibodies (aT) via MMP-cleavable linkers (aT-cl-M1ev, termed ACE). Upon intravitreal injection in female PVR model mice, ACE selectively accumulates in lesions, where released antibodies neutralize TGFβ1 and M1evs inhibit M2 macrophage polarization, modulating the microenvironment to diminish vitreoretinal fibrosis. Further incorporating anti-platelet-derived growth factor receptor antibodies yields aTP-cl-M1ev (ACE^Plus^) to prevent retinal detachment progression in advanced-stage PVR, an efficacy validated in a patient-derived PVR membrane xenograft model. Therapeutic modulation of the vitreoretinal fibrosis microenvironment with the ACE platform provides an efficacious alternative to surgery for PVR.

## Abstract (web summary)

Vitreoretinal fibrosis is a hallmark of proliferative vitreoretinopathy, a severe blinding disease that necessitates surgery. Here, the authors show that engineered extracellular vesicles therapeutically modulate this microenvironment to effectively inhibit fibrosis and prevent further retinal detachment.

## Introduction

Vitreoretinal fibrosis is a common pathological feature characterized by excessive extracellular matrix (ECM) deposition and fibrotic membrane formation. As an intractable vitreoretinal fibrosis disease, proliferative vitreoretinopathy (PVR) is thought to be an abnormal wound healing response^1–3^. It exerts tractional forces on the retina, potentially exacerbating retinal detachment (RD) and subsequent vision loss or even blindness. Among patients with open globe injuries and rhegmatogenous RD, the incidence of PVR is 40–60% and 8–10%^4,5^, respectively. Currently, the clinical treatment for PVR relies on surgical removal of tractional membranes with poor prognosis^6–8^. Furthermore, this approach is invasive and carries a substantial risk of recurrence^2^. Accordingly, there is an unmet clinical need to develop alternative pharmacological treatments for such a difficult-to-treat disease.

Inspired by the success of anti-tumor strategies that target tumor microenvironment^9–12^, we envisioned that the vitreoretinal fibrosis microenvironment could also be the target to develop a viable and effective pharmacological treatment for PVR. According to previous studies, the blood-retinal barrier is disrupted at an early stage of PVR, leading to the detachment of retinal pigment epithelial (RPE) cells from Bruch’s membrane. Infiltration of immune cells and secretion of cytokines shape the microenvironment, thereby promoting the transition of RPE cells from a quiescent to an active state^13^. However, current understanding of the vitreoretinal fibrosis microenvironment in PVR is still largely at a descriptive level and lacks comprehensive and quantitative analysis, which makes it difficult to accurately identify key therapeutic targets within the complex microenvironment. To solve this problem, we performed data mining on an integrated dataset comprising our own and publicly available datasets (GSE179603) from PVR and non-PVR clinical samples^14^. Among cytokines, transforming growth factor β1 (TGFβ1) ranked highest among fibrosis-associated cytokines, while among immune cells, M2 macrophages were identified as the most prevalent cell type with statistically significant enrichment in PVR samples. These findings underscore both TGFβ1 and M2 macrophages as promising and rational targets for focused therapeutic intervention.

Given that only small amounts of drugs reached the retinal lesion site after intravitreal injection^15,16^, we further developed an extracellular vesicle-based therapy that enabled both spatiotemporal delivery and combined interventions against the above two targets, i.e., TGFβ1 and M2 macrophages. Briefly, we engineered extracellular vesicles derived from M1 macrophages (M1ev) and conjugated them with anti-TGFβ1 antibodies (aT) using a peptide linker (cl) that should undergo cleavage by matrix metalloproteinases (MMPs), which allowed aT to act in the ECM and M1ev to act on macrophages. After intravitreal injection, the formed aT-cl-M1ev (denoted as ACE) exploited the ability of M1ev to accumulate to PVR lesions. Upon MMPs-mediated cleavage, ACE separated into aT and M1ev to neutralize pathogenic TGFβ1 activity and inhibit M2 macrophage polarization, respectively. These two aspects together led to therapeutic modulation of the vitreoretinal fibrosis environment and potent anti-fibrotic benefits. For the treatment of PVR at an advanced stage, we further incorporated an anti-platelet-derived growth factor receptor α (PDGFRα) antibody (aP) on M1ev, which was demonstrated, especially, in an innovative patient-derived PVR membrane xenograft (PDMX) model.

## Results

### Data mining for depicting the vitreoretinal fibrosis microenvironment and verification in samples from PVR patients and model mice

**Fig. 1:**
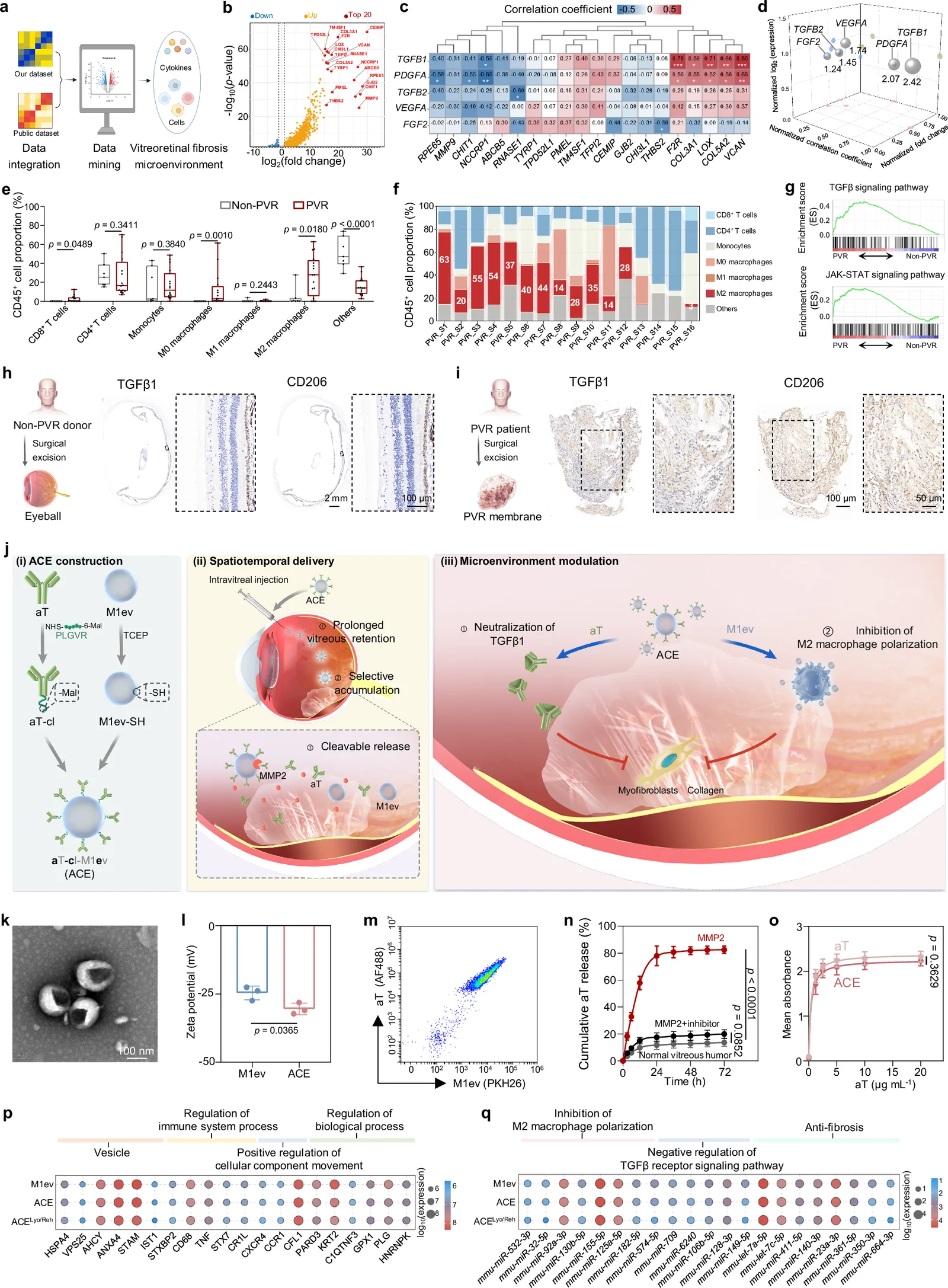
Depiction of the vitreoretinal fibrosis microenvironment and the design, fabrication, and characterization of ACE. **a** Schematic of data mining for ranking representative fibrosis-associated cytokines and performing cell enrichment analysis to depict the microenvironment. Created in BioRender. Tian, Y. (2026) https://BioRender.com/dgyk19l. **b** Volcano plot showing DEGs from bulk RNA-seq of PVR and non-PVR samples. **c** Correlation between representative fibrosis-related cytokines and top 20 DEGs. **d** Three-dimensional integrated ranking of representative cytokines. **e** CIBERSORTx cell enrichment analysis of RNA-seq from PVR (*n* = 16 PVR membranes) and non-PVR (*n* = 7 ILMs). **f** Relative immune cell abundance as a percentage of total infiltrates. **g** GSEA for gene sets changed in PVR vs. non-PVR. Left: schematic of surgical excision yielding a non-PVR donor eyeball (**h**) and PVR patient membrane (**i**). Right: IHC of sectioned non-PVR eyeball (**h**) and PVR membrane (**i**), showing TGFβ1 and CD206 expression. Outlined areas are magnified below. **j** Schematic of ACE construction, spatiotemporal delivery, and microenvironment modulation. **j-**i aT was conjugated to tris(2-carboxyethyl)phosphine (TCEP)-treated M1ev via an MMP2-responsive linker PLGVR. **j-**ii Intravitreally injected ACE exhibited prolonged vitreous retention, selective accumulation, and controlled release at PVR lesions. **j-**iii Upon MMP2-responsive cleavage, aT neutralized TGFβ1, while M1ev inhibited M2 macrophage polarization. **k** Representative TEM image. **l** Zeta potential of M1ev before and after loading aT. **m** Colocalization efficiency between aT and M1ev. **n** In vitro, aT release in vitreous humor under PVR or normal MMP2 levels, with or without an inhibitor. **o** TGFβ1-binding capacity of ACE vs. free aT. Proteomics (**p**) and miRNA sequencing (**q**) of fresh M1ev, ACE, and ACE^Lyo/Reh^. Data in (**b**) were analyzed using a two-sided Wald test (DESeq2) with Benjamini–Hochberg adjustment. Data in (**e**) are presented as medians (25th–75th quartiles) and were compared using a two-tailed Mann–Whitney *U* test. Data in (**l**, **n**, **o**) are presented as mean ± s.d., *n* = 3 biologically independent samples. Data in (**l**, **o**) were compared using unpaired two-tailed Student’s *t* test. Data in (**n**) were compared using one-way ANOVA. All *p*-values are indicated. Source data are provided as a Source Data file.

To depict the vitreoretinal fibrosis microenvironment and identify potential intervention targets, we performed data mining on the integrated dataset (Fig. 1a), which included 16 PVR membranes (representing fibrotic samples) from PVR patients and seven internal limiting membranes (serving as non-fibrotic samples) from non-PVR patients. We focused on infiltrating immune cells and secreted cytokines, which together shaped the vitreoretinal fibrosis microenvironment. Initially, differentially expressed genes (DEGs) were extracted to gain a comprehensive view of the vitreoretinal fibrosis microenvironment (Fig. 1b). To identify the key cytokine, we assessed the correlation between representative fibrosis-related cytokines^17–19^ and the top 20 DEGs (Fig. 1c). Moreover, we quantified the cytokines’ relative abundance and analyzed the cytokines’ fold changes. After integration and normalization of the results from the above three aspects, TGFβ1 ranked highest (Fig. 1d). To identify the key immune cell type, we performed cell enrichment analysis using CIBERSORTx and revealed that M2 macrophages were the most prevalent cell type (Fig. 1e, f)^20,21^. Gene set enrichment analysis (GSEA) further demonstrated that TGFβ signaling and the JAK-STAT signaling pathways associated with M2 macrophages were significantly enriched in the PVR group, highlighting the pivotal role of cytokine TGFβ1 and M2 macrophages in the vitreoretinal fibrosis microenvironment (Fig. 1g).

To validate these findings, we collected PVR membranes from PVR patients and a cornea-free eyeball from a non-PVR donor (Supplementary Table 1), which was used for immunohistochemical (IHC) staining. Similar to α-smooth muscle actin (α-SMA) and Collagen1 (fibrotic markers), TGFβ1, CD206, and CD163 (M2 macrophage-associated markers) exhibited higher expression levels in the PVR membrane samples (Fig. 1h, i, and Supplementary Fig. 1a–c). Such upregulation was also validated in PVR model mice induced by intravitreal injection of gas and primary mouse RPE (mRPE) cells (Supplementary Fig. 1d–h)^22^. The above results from the data mining and IHC staining analysis highlighted TGFβ1 and M2 macrophages as pivotal molecular and cellular elements within the vitreoretinal fibrosis microenvironment.

### Design, fabrication, and characterization of ACE

This particular combination of key PVR pathological microenvironment features inspired us to design a therapeutic with dual modes of action to treat PVR. Our initial focus was on neutralizing the excessive expression and activation of TGFβ1 using therapeutic antibodies. In parallel, we aimed to inhibit M2 macrophage polarization using M1ev, and the chemokine receptors expressed on the extracellular vesicle surface indicated that the natural concentration gradient of their ligands^23–25^, from the vitreous cavity to fibrotic lesions, would drive the accumulation at PVR sites. Additionally, the aT was conjugated onto M1ev via an MMP2-cleavable linker PLGVR^26,27^, which took advantage of the high levels of MMP2 at the PVR lesion site (Supplementary Fig. 2). Upon such spatiotemporal delivery, aT and M1ev were released precisely at the lesion site, thereby establishing a therapeutic microenvironment by neutralizing TGFβ1 and inhibiting M2 macrophage polarization without mutual interference (Fig. 1j).

Characterization of ACE with transmission electron microscopy (TEM) indicated a cup-like morphology (Fig. 1k) and an average diameter of approximately 130 nm. The successful conjugation of aT on the surface of M1ev, derived from macrophages with an M1-dominant phenotype (Supplementary Fig. 3a), was verified by Western blotting analysis, showing the coexistence of extracellular vesicle markers (CD63, ALIX, and TSG101), M1 macrophage specific protein (iNOS), and conjugated aT (Supplementary Fig. 3b). Non-extracellular vesicle markers (LDHA, LDHB, and HSPA13) were absent, confirming the purity of the isolated extracellular vesicles^28^ (Supplementary Fig. 3c). Accordingly, comparing ACE to M1ev, we observed that nanoparticle tracking analysis (NTA) detected a slight decrease of zeta potential (Fig. 1l) and nano-flow cytometry revealed good colocalization efficiency of aT and M1ev (Fig. 1m). After calculation, each extracellular vesicle was conjugated with approximately 10 aT molecules, indicating the conjugation was efficient.

To test the cleavability of the cl linker, we incubated ACE with MMP2 and detected the proportion of the aT released from ACE. As expected, the proportion of aT increased up to 78.0% within 24 h, indicating that most of the cl linker had been cleaved. In contrast, the cl linker was stable and released negligible aT at MMP2 levels found in normal vitreous humor. Once the MMP2 inhibitor was incubated, the proportion of aT dramatically decreased (Fig. 1n), supporting the specificity of cleavage. Moreover, the amount of neutralized TGFβ1 showed a dose-dependent manner in both the ACE (released aT) and aT (free and unconjugated form) group, with no significant differences observed between these two groups (Fig. 1o). This suggested that the conjugation and release cycle of aT did not impair its neutralizing capacity.

Next, our evaluations shifted to the vesicle component, especially in the aspect of stability. We performed a proteomics analysis of the unconjugated form (M1ev), the fresh state, and the state after lyophilization and rehydration (Lyo/Reh). Proteomics analysis revealed comparable abundances of characteristic proteins related to vesicle markers, regulation of the immune system process, positive regulation of cellular component movement, and regulation of biological process (Fig. 1p). microRNA (miRNA) sequencing also revealed that the abundances of representative miRNAs related to inhibition of M2 macrophage polarization, negative regulation of TGFβ receptor signaling pathway and anti-fibrotic function remained consistent across the three groups (Fig. 1q). Note that ACE^Lyo/Reh^ incubated in PBS at 4 °C for 7 days showed no detectable changes in particle size or zeta potential (Supplementary Fig. 3d). Such good stability indicated that the M1evs preserved their activity following the processes of modification and lyophilization. We can also expect the potential of ACE as an off-the-shelf product that is well-suited for on-demand settings.

### Intraocular retention, accumulation, and release of ACE

**Fig. 2:**
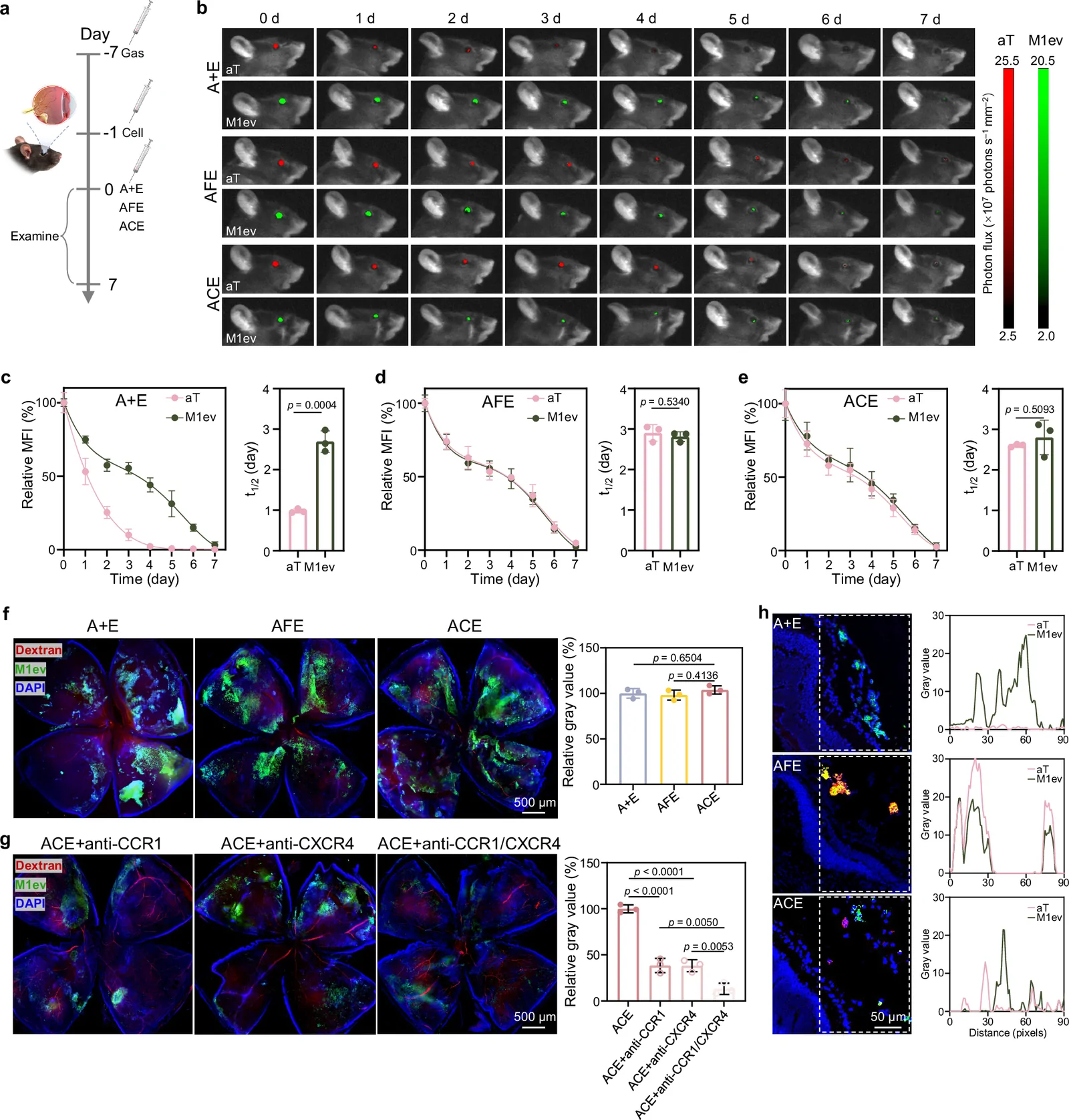
Intraocular behavior of ACE in PVR model mice: retention, delivery, and release. **a** Schematic showing the experimental design for evaluation of ACE after intravitreal injection in PVR model mice. **b** Representative in vivo fluorescence imaging of PVR model mice at the indicated timepoints after indicated treatments (green, Cy7-SE-labelled M1ev; red, Alexa Fluor 647-labelled aT). Left: relative mean fluorescence intensity (MFI) over time in the A+E (**c**), AFE (**d**), and ACE (**e**) groups. Right: calculated half-lives of aT and M1ev in the A+E (**c**), AFE (**d**), and ACE (**e**) groups (*n* = 3, 1 eye from each of 3 mice). Representative images and quantification of M1ev accumulation in flat-mounted retinas of PVR model mice on day 1 following indicated treatments (**f**) and ACE treatment with indicated blocking strategies (**g**) (*n* = 3, 1 eye from each of 3 mice; green, PKH67-labelled M1ev; red, dextran-labelled retinal vessels; blue, DAPI-labelled cell nuclei). **h** Left: representative CLSM images of frozen retinal tissue sections from PVR model mice, showing localization of M1ev and aT at PVR lesions (Red, Alexa Fluor 647-labelled aT; green, PKH67-labelled M1ev). Right: quantification of the localization of M1ev and aT for the corresponding images. The red line shows the variation in fluorescence intensity of aT within the area covered by the white dashed rectangular selection; The green line shows the variation in fluorescence intensity of M1ev within the same selection. Data in (**c**–**g**) are presented as mean ± s.d. Data in (**c**–**e**) were compared using unpaired two-tailed Student’s *t* test. Data in (**f**, **g**) were compared using one-way ANOVA. All *p*-values are indicated. Source data are provided as a Source Data file.

With the ACE in hand, we next evaluated the intraocular behavior in PVR model mice. Briefly, gas and mRPE cells were injected into the mouse vitreous cavity on day −7 and day −1, respectively, to simulate PVR pathogenesis^22^. On day 0, the model mice were randomly divided into three groups, which received intravitreal injections of a mixture of aT and M1ev (A+E), aT conjugated M1ev via a fixed (MMPs-uncleavable) linker (aT-fl-M1ev, AFE), or ACE (Fig. 2a). To investigate the retention, aT and M1ev were labeled with Alexa Fluor 647 and Cy7-SE, respectively, and their signals were simultaneously captured by an in vivo imaging system (Fig. 2b). In all groups, M1ev fluorescence signals gradually decreased in almost the same manner, with the disappearance consistently at day 7. On the contrary, aT fluorescence varied among the three groups: the fluorescent signal of free aT in the A+E group declined rapidly and nearly disappeared at day 3, whereas around 50% of the initial aT signal remained detectable after 3 days in groups AFE and ACE, indicating that the conjugation on M1ev reduced aT clearance from the vitreous cavity. After calculation, the half-life of aT in group A+E was 1.0 day (Fig. 2c), which was less than half of that in group AFE and ACE (Fig. 2d, e).

Considering PVR primarily occurs in the preretinal space, we next investigated the intraocular accumulation via retinal flat-mount imaging of PKH67-labeled M1ev. As shown in Fig. 2f, a substantial accumulation of M1ev was observed on the preretinal surface, with no significant differences among the groups of A+E, AFE, or ACE. This suggested that extracellular vesicles had an inherent ability to reach the lesions. To reveal the underlying mechanism, we repeated the evaluation by pretreating the M1ev with antibodies against C–C chemokine receptor type 1 (CCR1) and C–X–C chemokine receptor type 4 (CXCR4), either individually or in combination, since these two receptors were highly expressed on ACE in proteomic analysis (Fig. 1p), and their ligands were highly expressed in a PVR mouse model (Supplementary Fig. 4a). Taking ACE for an example, M1ev levels decreased by 61.5%, 61.8% and 86.8% in the ACE+anti-CCR1, ACE+anti-CXCR4, and ACE+anti-CCR1/CXCR4 groups, respectively (Fig. 2g). Similarly, a consistent effect was observed in the gene knockout setting (Supplementary Fig. 4b–e). Together, these findings confirm that the accumulation to PVR lesions is primarily mediated by CCR1 and CXCR4.

Moreover, we analyzed retina sections to investigate the release of aT following accumulation at lesions. In contrast to the pronounced overlap of aT and M1ev signals observed in AFE-treated mice and the significantly reduced aT signals in the A+E group, a notable separation between aT and M1ev signals was evident in the ACE group (Fig. 2h and Supplementary Fig. 4f). This difference should be attributed to the efficient cleavage of the linker by the elevated levels of MMP2 in PVR lesions. These results demonstrated that the ACE underwent cleavage-mediated release at PVR lesions, enabling the antibody and extracellular vesicles to function independently without interference.

### Therapeutic efficacy of ACE in the PVR model mice

Encouraged by the above efficient delivery, we investigated the in vivo therapeutic efficacies. The model mice were divided into six groups and received a single intravitreal injection of either PBS, aT, M1ev, A+E, AFE, or ACE on day 0. On day 28, anti-fibrotic effect and visual function were evaluated. Note that we also conducted a dose escalation study before conducting the above evaluations, with the optimized dose set as 2 μg aT and 10 μg M1ev (Supplementary Fig. 5).

**Fig. 3:**
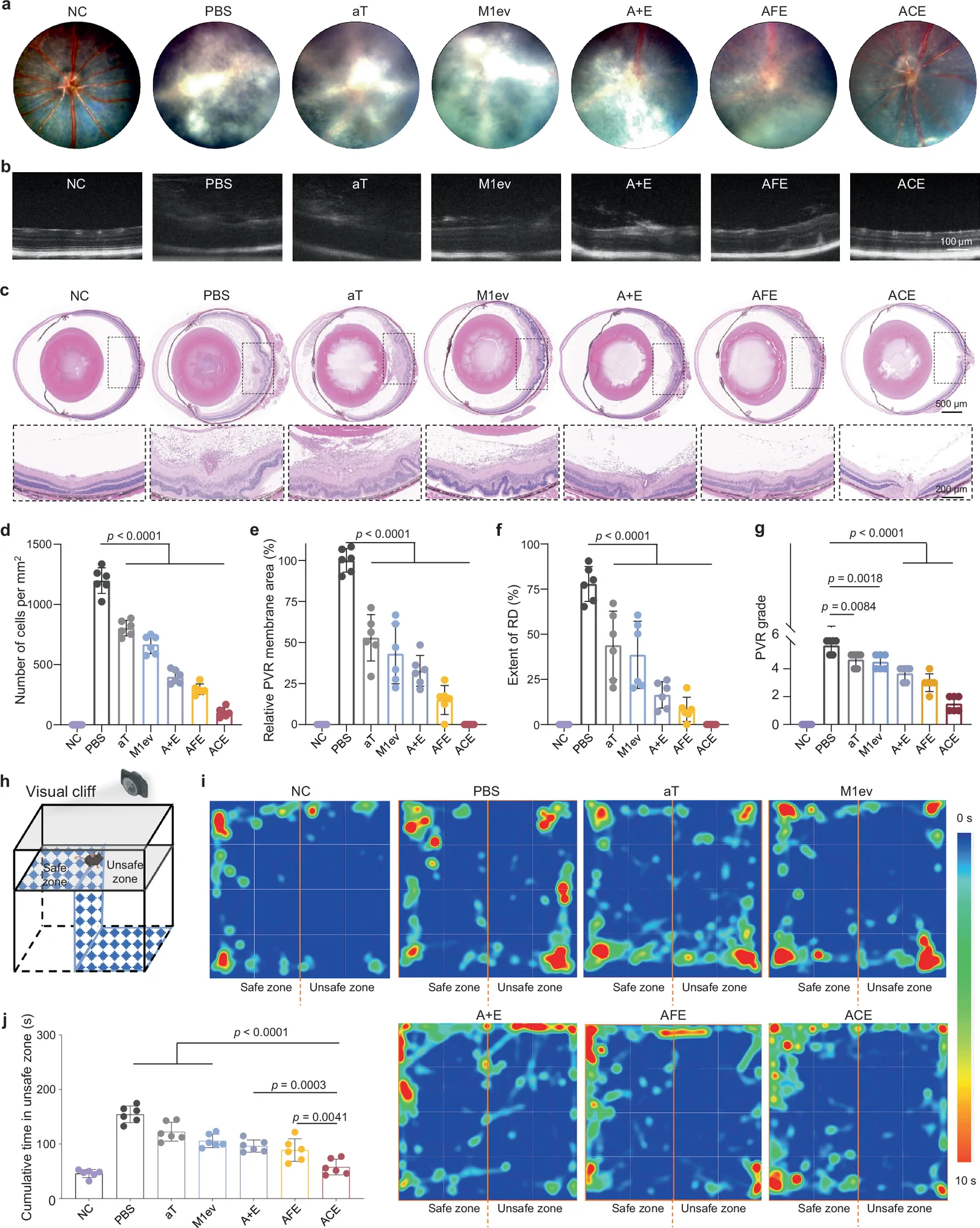
Anti-fibrotic efficacy of ACE at an early stage of the PVR model mice. Representative CFP (**a**) and OCT (**b**) images of NC and PVR model mice on day 28 after indicated treatments. The images are presented at the same magnification. **c** Representative H&E staining of retinal slices from PVR model mice of the indicated treatment groups on day 28. The images are presented at the same magnification. The outlined areas are magnified in the bottom sub-panels. Quantitative analysis of the number of cells in the vitreous cavity (**d**), relative PVR membrane area (**e**), extent of retinal detachment (RD) (**f**), and PVR grade (**g**, based on clinical assessment, CFP, OCT, and H&E staining; see Supplementary Table 2 for criteria) (*n* = 6, one eye from each of six mice). **h** Schematic for the “Visual Cliff” paradigm for behavioral testing. Created in BioRender. Tian, Y. (2026) https://BioRender.com/yextdle. **i** Representative heatmaps tracking the time spent at each position by NC mice and PVR model mice across indicated treatment groups (left: safe zone; right: unsafe zone). **j** Quantification of the cumulative time spent in the unsafe zone across indicated groups (*n* = 6 mice). Data in (**d**–**g**, **j**) are presented as mean ± s.d. Data in (**d**–**g**, **j**) were compared using one-way ANOVA. In **g**, ACE resulted in a mean difference of 4.17 (95% confidence interval: 3.02–5.31) compared to PBS. All *p*-values are indicated. Source data are provided as a Source Data file.

As shown in Fig. 3a–c and Supplementary Fig. 6a–e, color fundus photography (CFP), optical coherence tomography (OCT), and H&E staining revealed that mice treated with PBS showed a central hypopigmented area corresponding to the optic nerve, along with a large PVR membrane infiltrated by numerous cells and extensive RD. After treatments, the number of infiltrating cells, the area of PVR membrane, the extent of RD, and the PVR grade gradually decreased in the sequence of monotherapies (aT and M1ev) and the combination treatments (A+E, AFE, and ACE) (Fig. 3d–g). Compared with the PBS group, the PVR grade in group ACE were reduced by 3.8-fold, whereas this therapeutic efficacy was significantly attenuated by MMP2 inhibition (Supplementary Fig. 6f). Note that the area of PVR membrane formation and the extent of RD were comparable to those observed in the non-model control (NC) group, suggesting therapeutic potential in suppressing the progression of vision-threatening PVR.

Next, we examined whether above treatments improved visual function using the visual cliff test^29,30^, which assessed the innate tendency of mice to avoid the deep side (unsafe zone) of a visual cliff field (Fig. 3h). Briefly, the mouse was placed on the testing platform, and the cumulative time the mice spent in the safe and unsafe zones was recorded for 5 min (Fig. 3i). Owing to the extensive PVR membrane formation and widespread RD, PBS-treated mice had no preference for the safe or unsafe zone. On the contrary, mice in the treatment groups showed significantly reduced times in the unsafe zone. As expected, the ACE group again outperformed other treatments, with a 2.7-fold decrease in time spent in the unsafe zone as compared to the PBS group (Fig. 3j).

In addition to the above therapeutic efficacy, the ACE treatment did not alter intraocular pressure (Supplementary Fig. 7a) or anterior segment morphology (Supplementary Fig. 7b), and no detectable signs of intraocular inflammation were observed following intravitreal injection (Supplementary Fig. 7c), collectively supporting the apparent safety of ACE upon local application. Moreover, the ACE treatment did not induce any detectable changes in hematological parameters, serum biochemical indices or any changes to histological characteristics in the heart, liver, spleen, lung, or kidney (Supplementary Fig. 7d–f), supporting this local treatment without apparent side effects to other tissues.

### Therapeutic modulation of the vitreoretinal fibrosis microenvironment

**Fig. 4:**
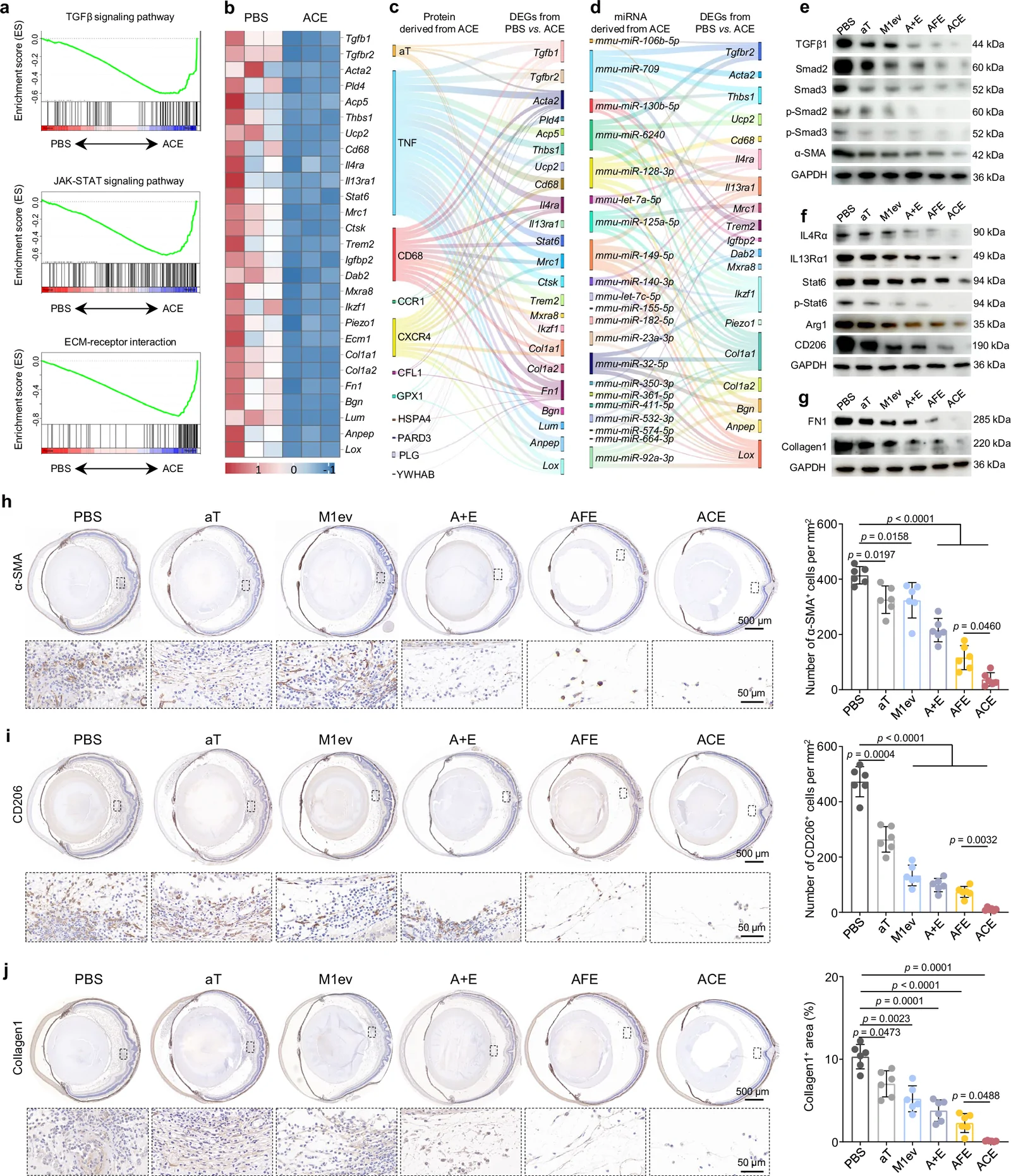
Therapeutic modulation of the vitreoretinal fibrosis microenvironment after ACE treatment. **a** GSEA for gene sets that were changed in the ACE treatment group vs. the PBS group. **b** Heatmap of DEGs from retinal tissue transcriptome analysis upon intravitreal injection of PBS or ACE in the PVR model mice. Sankey plots (ACE vs. PBS) illustrating inferred interactions between ACE proteins (**c**, left) or ACE-enriched miRNAs (**d**, left) and DEGs with known functions in vitreoretinal fibrosis regulation (right). **e**–**g** Western blot of retina tissues from PVR model mice on day 28 after intravitreal injection of indicated treatments; GAPDH was used as a loading control and for signal normalization. Left: representative IHC staining for α-SMA (**h**), CD206 (**i**), and Collagen1 (**j**) in ocular sections of PVR model mice across indicated treatment groups on day 28. Outlined areas are magnified in the bottom sub-panels. Right: quantification of α-SMA-positive cells (**h**), CD206-positive cells (**i**), and Collagen1-positive area (**j**) on the PVR membrane in the vitreous cavity (*n* = 6, one eye from each of six mice). Data in (**h**–**j**) are presented as mean ± s.d. and were compared using one-way ANOVA. All *p*-values are indicated. Source data are provided as a Source Data file.

We subsequently performed transcriptome profiling to investigate the biomolecular basis of the observed therapeutic benefit upon ACE treatment (Supplementary Fig. 8). We sampled retinal tissues from PVR model mice treated with PBS or ACE and performed RNA-seq. GSEA indicated inter-group transcriptome differences for three gene sets (“TGFβ signaling pathway,” “JAK–STAT signaling pathway,” and “ECM–receptor interaction”) (Fig. 4a). We then identified genes from these sets, supplemented by literature-curated genes associated with TGFβ signaling^31,32^, M2 macrophages^33,34^, and ECM components^35,36^, that were differentially expressed in ACE vs. PBS treated retinal tissues (Fig. 4b), and used these DEGs as inputs for two analyses of potential interactions with the biomolecules (proteins and miRNAs) we earlier characterized in the extracellular vesicles used to generate ACE (Fig. 1p, q). Briefly, these STRING- and miRanda-based analyses offered additional evidence supporting the potential involvement of retinal tissue molecules in the therapeutic benefit (Fig. 4c, d), which we next examined via Western blotting for the full set of six interventions used to treat PVR model mice.

The TGFβ–SMAD pathway has been previously implicated in myofibroblast activation^37^, including in the context of retinal pathology^38^. We examined the levels of multiple TGFβ–SMAD pathway components in retinal tissues, including α-SMA, TGFβ1, Smad2/3, and p-Smad2/3^39,40^. Compared to the PBS group, both the monotherapies (aT and M1ev) and the combination treatments (A+E, AFE, and ACE) showed reduced expression levels of all measured proteins, with the ACE group exhibiting the most significant reduction (Fig. 4e and Supplementary Fig. 9). Given that multiple DEGs from our transcriptome analysis are known to function in M2 macrophage polarization, we detected consistent decreasing trends across the six groups for proteins including IL4Rα, IL13Rα1, Stat6, p-Stat6, and Arg1 (Fig. 4f and Supplementary Fig. 9b)^41–45^, supporting an impact from inhibited M2 macrophage polarization in the observed ACE therapeutic benefits on retinal pathology. Notably, we also found that the levels of fibronectin (FN1) and Collagen1 (both included in the ECM–receptor interaction gene set) showed a similar decreased trend across the six groups (Fig. 4g and Supplementary Fig. 9c), lending additional support to anti-fibrotic impacts from each ACE component. Collectively, the convergence of these findings provides support for our rationale that inhibiting M2 macrophage polarization and neutralizing TGFβ1 drives the therapeutic efficacy. Establishing a direct causal link to confirm that the benefits are strictly M2-macrophage-dependent and that TGFβ1 suppression is the direct driver preventing myofibroblast transition would necessitate future investigations using cell-specific genetic models.

We then extended our investigation beyond the bulk RNA-seq and tissue lysate samples, specifically using IHC staining to examine in situ impacts of ACE in retinal slices of PVR model mice, including for α-SMA expression in activated myofibroblasts, for CD206-positive M2 macrophages, and for Collagen1 deposition on the PVR membranes. Consistent with our Western blotting results, both the monotherapies and the combination treatments significantly reduced α-SMA levels in myofibroblasts and the number of M2 macrophages compared to the PBS group, with the ACE group conferring the maximal reduction (Fig. 4h, i, and Supplementary Fig. 10). Therefore, we observed a similar decreasing trend in the Collagen1-positive area across the six groups (Fig. 4j). These results collectively support that the observed therapeutic benefits conferred by ACE result from multiple biomolecular manipulations that suppress pro-fibrotic signaling and M2 macrophage polarization pathway.

### aTP-cl-M1ev (ACE^Plus^) construction and therapeutic efficacy in PVR model mice at an advanced stage

**Fig. 5:**
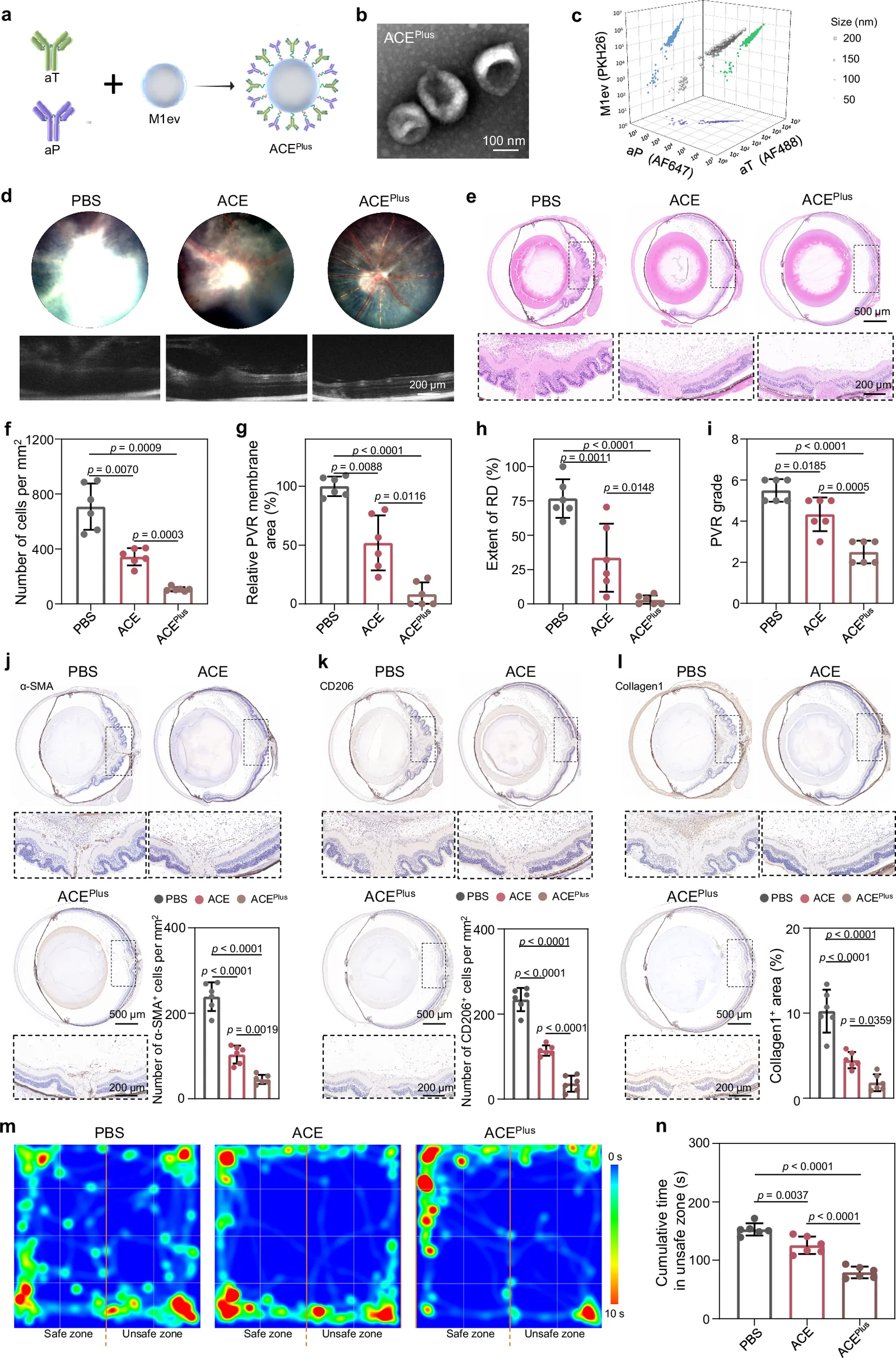
ACE^Plus^ construction and efficacy are at an advanced stage in the PVR model mice. **a** Schematic showing the conjugation of both aT and aP to M1ev. **b** Representative TEM image of ACE^Plus^. **c** Nano-flow cytometry assay of aT, aP, and M1ev colocalization. Representative CFP (top) and OCT (bottom) images (**d**), and H&E staining of ocular sections (**e**) from advanced-stage PVR model mice on day 28 after indicated treatments. Outlined areas in (**e**) are magnified below. Quantification of the number of cells in the vitreous cavity (**f**), relative PVR membrane area (**g**), extent of RD (**h**), and PVR grade (**i**, based on clinical assessment, CFP, OCT, and H&E staining; see Supplementary Table 2 for criteria) (*n* = 6, one eye from each of six mice). Top row and bottom left: representative IHC staining for α-SMA (**j**), CD206 (**k**), and Collagen1 (**l**) in ocular sections of advanced-stage PVR model mice across indicated treatment groups on day 28 (outlined areas are magnified below). Bottom right: quantification of α-SMA-positive cells (**j**), CD206-positive cells (**k**), and Collagen1-positive area (**l**) on the PVR membrane (*n* = 6, one eye from each of six mice). **m** Representative heatmaps tracking the time spent at each position by advanced-stage PVR model mice across indicated treatment groups on day 28 (left: safe zone; right: unsafe zone). **n** Quantification of the cumulative time spent in the unsafe zone across indicated groups (*n* = 6 mice). Data in (**f**–**l**, **n**) are presented as means ± s.d. Data in (**f**–**l**, **n**) were compared using one-way ANOVA. In **i**, the mean difference between the PBS and ACE^Plus^ groups was 3.00 (95% confidence interval: 2.03–3.97). All *p*-values are indicated. Source data are provided as a Source Data file.

In the clinic, most PVR patients are diagnosed with advanced stage. Aiming at efficient treatment of PVR in a severe condition, we herein proposed that our ACE could be iterated by conjugation with another drug that exerted anti-PDGFRα effect through a complementary mechanism. Given that our results of data mining revealed PDGFA as the second most strongly correlated cytokine with fibrosis markers, and considering the critical role of PDGFRα activation in membrane contraction during PVR progression^1,46–49^, we chose to block the PDGFA-PDGFRα axis. Correspondingly, aT and anti-PDGFRα (aP) were conjugated onto M1ev to develop ACE^Plus^ (Fig. 5a). Note that we introduced 1,2-distearoyl-sn-glycero-3-phosphoethanolamine-N-[poly(ethylene glycol)]-thiol (DSPE-PEG-SH) linker on the membrane of extracellular vesicles, which minimized competitive interference with aT conjugation.

As shown in Fig. 5b, ACE^Plus^ remained cup-like morphology, indicating the structure integrity. Compared to ACE, ACE^Plus^ showed a slightly increased particle size and a more negative zeta potential, indicating the success of aP conjugation (Supplementary Fig. 11a). Nano-flow cytometry analysis showed the colocalization of aT, aP, and M1ev, further suggesting the successful construction of ACE^Plus^ (Fig. 5c). As shown in Supplementary Fig. 11b, ACE^Plus^ enabled cleavable release of both aT and aP upon incubation with MMP2. Meanwhile, the released aT and aP retained their antigen-binding activity (Supplementary Fig. 11c). As expected, ACE^Plus^ exhibited good stability, showing a slight change in particle size and zeta potential after a lyophilization and rehydration cycle (Supplementary Fig. 11d).

For the assessment of ACE^Plus^ at an advanced PVR mouse model, intravitreal injections (PBS, ACE, or ACE^Plus^) were administered on day 7 after the mRPE injection, and evaluations of CFP, OCT, and H&E staining were conducted on day 28 (Fig. 5d, e, and Supplementary Fig. 12a–c). Both ACE and ACE^Plus^ attenuated cell infiltration, PVR membrane formation, RD, and PVR grade at an advanced stage in model mice (Fig. 5f–i), while ACE^Plus^ exhibited a more pronounced therapeutic effect across multiple pathological features. This enhanced effect was likely attributable to ACE^Plus^’s superior inhibition of PVR membrane contraction, as reflected by a 26.8-fold reduction in RD compared to the PBS group, whereas ACE achieved only a 2.3-fold reduction. This inhibition effectively suppressed PVR progression and disrupted the pathological vicious cycle. These results indicated that ACE^Plus^ effectively attenuated the major pathological processes of advanced PVR, underscoring its capacity to modulate the vitreoretinal fibrosis microenvironment.

Subsequently, IHC analysis was performed to further investigate the modulation of the vitreoretinal fibrosis microenvironment and the extent of fibrotic remodeling. Specifically, both ACE and ACE^Plus^ reduced fibroblast and M2 macrophage levels compared to the PBS group, with ACE^Plus^ achieving a greater reduction (Fig. 5j, k, and Supplementary Fig. 12d, e). Collagen1 deposition was also attenuated in both groups, with ACE^Plus^ exhibiting a stronger antifibrotic effect (Fig. 5l). Moreover, ACE^Plus^ led to a more pronounced improvement in visual performance (Fig. 5m, n). In addition to the above potent therapeutic outcomes, ACE^Plus^ was also demonstrated to be safe for treating these mice (Supplementary Fig. 13), further strengthening the promise as a modality for advanced-stage PVR therapy. These findings suggested that ACE^Plus^, which incorporated aP, served as a promising therapeutic approach for effectively treating advanced-stage PVR.

### Development of a patient-derived xenograft PVR model for assessing therapeutic efficacy

Encouraged by the benefits in the treatment of PVR mice, we wanted to extend our investigation into a more clinically relevant model. Inspired by the success of patient-derived tumor xenografts (PDX) in oncology research^50–52^, we envisioned that a patient-derived xenograft PVR model, ideally recapitulating the original characteristics of the patient (such as profibrogenic cytokines and cells within the vitreoretinal fibrosis microenvironment), could be a useful tool for the preclinical evaluation of our ACE platform. As we are unaware of any such mouse models, we established what we term a PDMX based on transplanting a clinical PVR membrane specimen into immunodeficient mice.

**Fig. 6:**
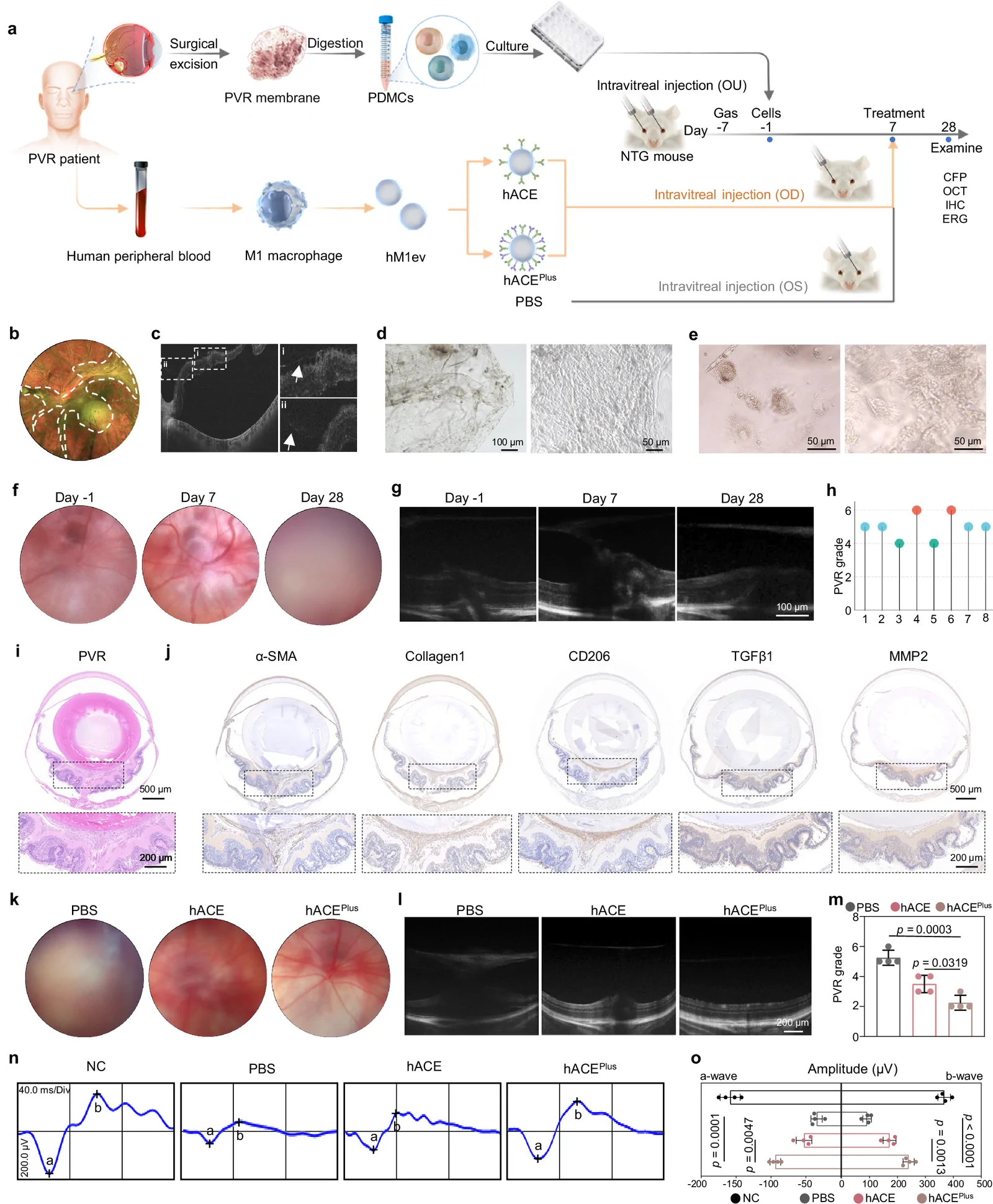
Anti-fibrotic efficacy of haTP–cl–M1ev (hACE^Plus^) at an advanced stage in PDMX model mice. **a** Schematic for the establishment of the PDMX and evaluation of the therapeutic efficacy of hACE^Plus^. Representative CFP (**b**) and OCT (**c**) images of patient eyes with PVR membranes. The white dashed line (**b**) and white arrow (**c**) indicate PVR membrane growth. Representative brightfield images of surgically resected patient PVR membranes (**d**) and in vitro cultured PDMCs on days 1 and 3 (**e**). Representative CFP (**f**) and OCT (**g**) images tracking PDMX model progression on days −1 (floating cells in the vitreous), 7 (initial PVR membrane formation), and 28 (thickened membrane and extensive RD) post-induction. **h** Quantification of PVR grade evaluating the establishment of the PDMX model (*n* = 8, one eye from each of eight mice; based on clinical assessment, CFP, and OCT; see Supplementary Table 2 for criteria). The model demonstrated consistent progression, with 75% reaching grade 5 or higher on day 28. Representative H&E staining (**i**) and IHC staining for α-SMA, Collagen1, CD206, TGFβ1, and MMP2 (**j**) in ocular sections of PDMX mice on day 28. The outlined areas in (**j**) are magnified below. Representative CFP (**k**) and OCT (**l**) images of PDMX mice across the indicated treatment groups on day 28. **m** Quantification of PVR grade across indicated treatment groups on day 28 (*n* = 4, one eye from each of four mice; based on clinical assessment, CFP, and OCT, see Supplementary Table 2 for criteria). Representative full-field ERG waveforms (**n**) and quantification of a-wave and b-wave amplitudes (**o**) across indicated treatment groups on day 28 (*n* = 4, one eye from each of four mice). Data in (**m**, **o**) are presented as mean ± s.d. and were compared using one-way ANOVA. All *p*-values are indicated. Source data are provided as a Source Data file.

The PDMX model is based on the principles of the PDX model and the gas- and cell-induced PVR animal model approach. The methodological steps included: administration of gas, digestion of the PVR membrane into a single-cell suspension, in vitro cultivation, and subsequent injection of cultured cells into the vitreous cavity (Fig. 6a). Specifically, surgically peeled PVR membrane samples were obtained from the vitreous cavity of PVR patients, who were diagnosed with grade C2 or higher by fundus and OCT images (Fig. 6b–d and Supplementary Table 1)^4^. The samples were dissected into small pieces and digested into a single-cell suspension. To ensure effective cell separation while minimizing damage to the cells from the digestion solution, we adopted a batch digestion approach, with centrifuging every 30 min to separate the already detached cells from the membrane. These patient-derived PVR membrane cells (PDMCs) were then cultured in epithelial cell-specific culture medium with 20% FBS^53,54^. After 72 h, the number of these cells increased 2.6-fold (Fig. 6e and Supplementary Fig. 14a), supporting proliferative capacity and viability.

Above cultured PDMCs were then intravitreally injected into female NOD-*Prkdc*^*scid*^/*Il2rg*^*null*^/Spf (NTG) mice, which had received an intravitreal injection of gas 6 days prior (day −7). Using CFP and OCT imaging, we monitored the progression of the PDMX model. Owing to the administration of PDMCs, the fundus immediately appeared hazy and unclear. Six days later, the proliferated PDMCs gradually formed a PVR membrane along the blood vessels, which contracted and resulted in a mild RD. After 4 weeks, we observed extensive RD (Fig. 6f, g), with an average detachment severity of 63.8% (Supplementary Fig. 14b). At this time point, the PVR grade scores were ≥grade 4 for all PDMX mice on day 28, indicating minimal variability across replicates (Fig. 6h). H&E staining of ocular sections from PDMX mice revealed characteristic pathological features associated with PVR (Fig. 6i). Notably, compared to the non-model control (NC) group, IHC staining showed significantly elevated levels for α-SMA, Collagen1, TGFβ1, M2-associated markers (CD206 and CD163) and MMP2 for the patient-derived PVR membrane (Fig. 6j and Supplementary Fig. 14c–e), supporting that this PDMX model informatively recapitulates key features of the PVR microenvironment.

### Evaluation of humanized ACE therapeutics (hACE and hACE^Plus^) in the PDMX model

Finally, we prepared hACE and hACE^Plus^ and tested its therapeutic potential in the PDMX model (Fig. 6a). Briefly, human M1 macrophages were obtained from human peripheral blood that were polarized using a routine differentiation protocol. The secreted human M1evs were then used as the chassis for conjugation with the antibody. Upon confirming the safety profile of hACE^Plus^ (Supplementary Fig. 15), PDMX model mice received a single intravitreal injection on day 7, with CFP and OCT imaging conducted on day 28 (Fig. 6k, l). For the PDMX model at an advanced stage, hACE treatment attenuated cellular proliferation and PVR membrane formation. Once we upgraded the therapeutic to hACE^Plus^, it promptly mitigated PVR membrane contraction, relieved RD, and modulated the pathological microenvironment loop. Notably, the hACE^Plus^ group showed significantly lower PVR grade scores than the hACE group (Fig. 6m).

We also used electroretinogram (ERG) to evaluate the potential therapeutic benefit on visual function^55,56^. Briefly, the amplitude of the a-wave represents the function of photoreceptor cells, while the amplitude of the b-wave is informative for the functions of both bipolar and Müller cells. In comparison with the NC group, the PBS group showed obvious reductions in the amplitudes of both a- and b-waves, with the electrophysiological curve being almost flat. In contrast, the reductions in the amplitudes of both the a- and b-waves were less pronounced for the hACE and hACE^Plus^ groups, with the hACE^Plus^ group again outperforming hACE for the treatment of the PDMX model at an advanced stage. Quantitatively, the hACE^Plus^ group exhibited a 2.7-fold increase in the a-wave and a 2.6-fold increase in the b-wave as compared to the PBS group (Fig. 6n, o). These data obtained using our PDMX model support that the hACE platform can be understood as a promising therapeutic platform for anti-fibrotic clinical interventions.

## Discussion

Inspired by the successful experience of targeting the tumor microenvironment, we aim to apply this strategy to the treatment of PVR, a challenging and refractory disease. To accurately identify key therapeutic targets within the complex microenvironment in PVR, we performed data mining on an integrated dataset comprising public databases and internally generated sample data, followed by the application of a multifaceted screening approach. Although the selection of TGFβ1, PDGFA, or M2 macrophages for targeted intervention has been reported for PVR treatment, we herein provided the rationalities. On one hand, through correlation coefficient analysis, we identified TGFβ1 and PDGFA as the top 2 associated fibrotic cytokines in PVR progression. On the other hand, cell enrichment analysis of the PVR samples revealed that M2 macrophages are the most abundant cell type in PVR samples. Considering cytokines and immune cells as two critical components of the microenvironment, we further adopted a rational combination strategy involving aT and M1ev for treating PVR, while aT, aP and M1ev treating PVR at an advanced stage.

This rational combination strategy was realized by conjugating antibodies onto extracellular vesicles via a PLGVR linker. Specifically, the NHS end of the linker reacted with amino groups on the antibody, while the maleimide end reacted with thiol groups on the vesicle surface. Through this design, the strategy conferred three major advantages: firstly, enhanced intraocular retention compared to free antibodies; secondly, selective accumulation at lesion sites via vesicle-displayed chemotactic receptors; and finally, site-specific antibody release triggered by cleavage of the MMP2-responsive linker in the pathological microenvironment. In the absence of this combination strategy, antibody monotherapy lacks the aforementioned advantages, exhibiting lower lesion-selective accumulation and reduced intraocular retention, thereby limiting its therapeutic duration to roughly 50% of that provided by our engineered platform^57^.

The flexibility of the ACE platform enables it to adapt to various clinical scenarios. Given that early-stage PVR is characterized by abnormal cellular proliferation predominantly driven by dysregulated TGFβ1 signaling and imbalance in M2 macrophage polarization, administration of ACE at this stage of PVR development (i.e., clinical stage prior to Grade C classification) holds promise for maximally preventing or delaying disease exacerbation. Moreover, ACE^Plus^, with aP exerting additional ability to inhibit membrane contraction, is better suited for treating advanced-stage PVR characterized by RD caused by PVR membrane contraction. Notably, when surgical intervention is unavoidable, pre-operative administration of ACE^Plus^ offers a potential treatment option, aiming to disrupt adhesions between proliferative membranes and the retina, thereby facilitating membrane dissection and improving ease of surgical removal. Even post-membrane peeling, the ACE platform remains a viable option to further mitigate the risk of PVR recurrence.

To address the challenges of conventional murine models in mimicking the complex pathophysiology of human PVR, we established the PDMX murine model by engrafting surgically excised patients’ PVR membranes into immunodeficient mice. This clinically relevant model faithfully recapitulated PVR pathology and the vitreoretinal fibrosis microenvironment, providing a useful tool for evaluating the therapeutic efficacy, especially for human-derived antibodies that are species-specific and incompatible with conventional murine models. Additionally, the PDMX model offers an opportunity for in vivo drug sensitivity testing and personalized treatment strategy development for PVR. Surgically excised PVR membranes, routinely discarded, can be repurposed to create individualized PDMX models. These patient-specific models enable direct assessment of therapeutic responses to a panel of existing or investigational agents, facilitating the identification of the most effective treatment strategy for each individual. This PDMX model serves as a versatile platform for precision medicine, applicable not only to PVR but potentially to other vitreoretinal fibrotic conditions that share fibrotic mechanisms, such as proliferative diabetic retinopathy and idiopathic epiretinal membrane, which are driven by aberrant cell migration, phenotypic transition, and excessive ECM deposition.

## Methods

### Reagents and materials

The peptide substrate Pro-Leu-Gly-Val-Arg (PLGVR), sensitive to protease cleavage, was sourced from ChinaPeptides Co., Ltd. Thiol-terminated 1,2-dis- tearoyl-sn-glycero-3-phosphoethanolamine-poly (ethylene glycol) (DSPE-PEG-SH, Mw 2000) was acquired from Shanghai ZZBIO Co., Ltd (Shanghai, China). Anti-TGFβ1 (ab215715), anti-Mannose Receptor (ab64693), anti-α-SMA (ab7817), anti-Collagen1 (ab88147), anti-MMP2 (ab86607), anti-CXCR4 (ab124824), anti-ALIX (ab275377), anti-TSG101 (ab125011), anti-CD63 (ab217345), anti-iNOS (ab178945), anti-GAPDH (ab181602), anti-Liver Arginase (ab124917), anti-Stat6 (ab32520), anti-phospho-Stat6 (Y641) (ab263947), Goat Anti-Rabbit IgG H&L (HRP) (ab205718), and Goat Anti-Mouse IgG H&L (HRP) (ab205719) antibodies were purchased from Abcam. Anti-HSPA13 (DF1307), anti-LDHA (DF6280), and anti-LDHB (DF12101) antibodies were purchased from Affinity. Anti-Smad2 (5339), anti-Smad3 (9523), anti-phospho-Smad2 (18338), and anti-phospho-Smad3 (9520) antibodies were purchased from Cell Signaling Technology. Anti-CCR1 (PA1-41062) antibody was purchased from ThermoFisher Scientific. Anti-IL4Rα (YT2337), anti-IL13Rα1 (YT2313), anti-FN1 (YM8309), and anti-Col1A1 (YM8576) were purchased from Immunoway. Recombinant proteins (mouse TGFβ1, MMP2) were purchased from R&D Systems, and the inhibitor GM6001 was from MedChemExpress. ELISA kits (TGFβ1, MMP2, TNF-α, IL-1β, and IL-6) and DAPI were from Solarbio. Fluorescent dyes were sourced from Thermo Fisher Scientific (Alexa Fluor 488, Alexa Fluor 594, and Alexa Fluor 647) and Sigma-Aldrich (PKH26 and PKH67). FITC anti-mouse CD80 (104705), PE anti-mouse F4/80 (123109), and APC anti-mouse CD206 (141708) antibodies were purchased from BioLegend. Cell culture was performed using Dulbecco’s Modified Eagle Medium (DMEM; HyClone), supplemented with fetal bovine serum (FBS; Cat. No. 16000044, Gibco) and penicillin–streptomycin (Corning Life Sciences). Mouse Retinal Pigment Epithelial Cell Complete Medium (CM-M116) was from Pricella Life Science & Technology. Endothelial basal medium 2 (EBM2) (190860), X-VIVO medium (04-418Q), glutamine (17-605E), and Endothelial Cell Growth Medium Bullet Kit (EGM) (CC-3124) were purchased from Lonza. The mouse retinal pigment epithelial cells (mRPE), ARPE-19 cells, and THP-1 cells were purchased from Pricella Life Science & Technology (Wuhan, China; Catalog Nos. CP-M116, CL-0026, and CL-0233, respectively).

### Collection of clinical samples

After surgical removal, clinical samples were processed according to their intended downstream applications. Some were snap-frozen in liquid nitrogen for transcriptomic analysis. Others were fixed in 10% neutral-buffered formalin for IHC staining. A portion of the specimens was preserved in Ringer’s solution and transported for subsequent cell isolation, in vitro culture, and animal model establishment. This study was approved by the Ethics Committee of Beijing Chaoyang Hospital, Capital Medical University (approval number: 2022-S-590). All participants provided written informed consent prior to sample acquisition. The participants consisted of 22 patients (15 males and 7 females), with a median age of 47.5 years (range: 22–81 years). All samples were destroyed according to standard procedures following the analysis.

### Data integration and RNA-sequencing analysis of clinical samples

Raw data of fastq format from the public dataset (GSE179603) and our dataset were separately processed through the fastp software. Clean reads were generated by removing adapter-containing sequences, reads with poly-N, and low-quality reads from the raw data. All the downstream analyses were based on the clean data with high quality. The reference genome and gene annotation files were downloaded from public genome databases. HISAT2 (v2.2.1) was employed to construct the genome index and to align both single-end and paired-end clean reads to the reference genome, creating splice-aware alignments to provide better alignment accuracy. And then, BAM files were generated, which are used for further analysis focused on quantification. Gene-level read counts were quantified using FeatureCounts (v2.0.6). The limma package was applied to correct for batch effects during data preprocessing. Differential expression analysis between the two groups was performed using the DESeq2 R package (v1.42.0), which provides statistical programs for determining differential expression in digital gene expression data using models based on a negative binomial distribution. The resulting *p*-values were adjusted using the Benjamini–Hochberg method to control the false discovery rate (FDR).

For visualization and reporting, gene expression levels were also normalized to fragments per kilobase of transcript per million mapped reads (FPKM). Differentially expressed genes were defined by an adjusted *p*-value (*p*adj) <0.05 and an absolute fold change ≥2.

Macrophage phenotypes were estimated using CIBERSORTx. Bulk RNA-seq data were uploaded to the CIBERSORTx platform, and the LM22 signature matrix was applied to deconvolute immune cell fractions. The relative abundance of macrophage subsets, including M0, M1, and M2 phenotypes, was calculated for each sample following the default parameters.

### IHC analysis of clinical samples

PVR membranes and the enucleated eye were promptly immersed in a fixative composed of formalin (10% neutral-buffered), ethanol (95%), and acetic acid in a 1:8.5:0.5 ratio. The preserved specimens were paraffin-embedded and subsequently sectioned at a thickness of 3–4 μm to maximize coverage of the tissue’s cross-sectional area. For IHC analysis, sections were incubated with anti-TGFβ1, anti-CD206, anti-MMP2, anti-α-SMA, and anti-Collagen1 antibodies at the recommended dilution. Following incubation with biotinylated secondary antibodies and color development, the IHC staining slides were scanned into digital images using a Pannoramic MIDI whole-slide scanner (3DHISTECH, Hungary). The acquired images were subsequently quantified and analyzed using Aipathwell software (version 2).

### Preparation of PVR models

PVR model mice were induced by intravitreal injection of gas, followed by an injection of RPE cells. Female C57BL/6J mice (6–8 weeks of age; 20 ± 1 g), selected to minimize confounding factors and ensure model reproducibility, were purchased from Charles River. Animals were housed under standardized environmental conditions, including a temperature of 23 °C, relative humidity of 55 ± 5%, and a 12-h light/dark cycle. Anesthesia was administered via intraperitoneal injection of 0.5% pentobarbital sodium at a dose of 0.1 mL per 10 g body weight. Mydriasis was induced using 1% tropicamide eye drops. Subsequently, 0.5 μL of sulfur hexafluoride (SF_6_, Alcon) was delivered into the vitreous chamber through a site 1 mm posterior to the limbus using a 10-μL Hamilton syringe fitted with a 33-gauge needle. The needle was left in place for 10 s to prevent gas egress. After 6 days (designated as day −1), the mouse received an intravitreal injection of 5 × 10^4^ mouse primary RPE (mRPE, Pricella Life Science & Technology) cells per eye by a 10-μL Hamilton syringe with a 33 G needle. The advanced stage of the PVR model mice can be assessed using a standardized grading scheme. This scheme, developed through fundus imaging and OCT, with confirmation from IHC analysis, was specifically tailored to the unique characteristics of the mouse model to ensure accurate evaluation. All animal procedures were conducted in accordance with protocols approved by the Institutional Animal Care and Use Committee of the Institute of Process Engineering, Chinese Academy of Sciences (IPEAECA 2021112). Specifically, animals were randomly assigned to treatment groups using a computer-generated randomization sequence, and investigators performing the assessments were blinded to the group allocations until data analysis was complete.

To assess the translational relevance of the ACE platform, a PDMX model of PVR was established. NTG mice aged 6–8 weeks (20 ± 1 g) were procured from SPF Biotechnology Co., Ltd. (Beijing, China). PVR membranes were obtained from PVR patients undergoing membrane-stripping surgery. These membranes were enzymatically digested into single-cell suspensions, and 1 × 10^4^ cells were used for xenotransplantation. The modeling procedure involved intravitreal injection of gas using a micro syringe, followed by injection of the prepared single-cell suspension 6 days later. CFP, OCT, ERG, and IHC were employed to monitor and assess the disease progression.

### IHC analysis of samples from the PVR model mice

Following enucleation, the eyeballs from the PVR model mice were immediately fixed in a solution consisting of 10% neutral-buffered formalin, 95% ethanol, and acetic acid at a ratio of 1:8.5:0.5. The fixed tissues were subsequently processed and embedded in paraffin. Serial sections of 4–5 μm thickness were prepared, extending from the pupil to the optic nerve. For IHC analysis, the sections were incubated with primary antibodies targeting TGFβ1, CD206, CD163, MMP2, α-SMA, and Collagen1, using the manufacturer-recommended dilutions.

### Measurement of MMP2 concentrations

Aqueous humor was extracted from the eyes of mice using a Hamilton microinjector. For each group, three pooled samples were prepared, each comprising fluid from six eyes. Vitreoretinal tissues were harvested from mice, homogenized in seven volumes of pre-chilled PBS supplemented with protease inhibitors, and sonicated on ice until fully disrupted. The homogenates were then centrifuged at 5000 × *g* for 5 min at 2–8 °C, and the resulting supernatants were collected for subsequent determination of MMP2 concentrations. MMP2 levels in these physiological and pathological samples were then quantified by ELISA to guide the subsequent in vitro assays.

### Induction of M1 macrophage

Mouse M1 macrophages were generated by isolating primary macrophages from the peritoneal cavity of C57BL/6J mice, followed by stimulation with lipopolysaccharide (LPS, 1 μg mL^−^^1^) for 48 h. After treatment, cells were thoroughly washed to remove residual LPS. The polarization status of the cells was assessed by flow cytometry, with cells first stained with Ghost V450 viability dye at 4 °C for 30 min to exclude dead cells, followed by surface staining with fluorophore-conjugated antibodies against F4/80 (PE), CD80 (FITC), and CD206 (APC), and finally analyzed to evaluate macrophage identity and predominant M1 polarization.

Human M1 macrophages were obtained by stimulating peripheral blood mononuclear cell (PBMC)-derived macrophages with LPS (1 μg mL^−1^) for 48 h, followed by washing to eliminate remaining LPS.

### Preparation of extracellular vesicles

Extracellular vesicles were collected from the culture supernatant of mouse or human M1 macrophages through a differential ultracentrifugation protocol. In brief, supernatants underwent sequential centrifugation steps to eliminate cells (300 × *g*, 10 min), cell fragments (2000 × *g*, 10 min), and larger debris (10,000 × *g*, 30 min, 4 °C). The clarified fluid was subsequently subjected to ultracentrifugation centrifuged at 100,000 × *g* for 70 min to pellet the extracellular vesicles. To improve purity, the pellets were washed with PBS and centrifuged again under identical conditions. The final extracellular vesicle preparations were resuspended in PBS and stored at 4 °C for short-term use (within 1 week) or at −80 °C for long-term storage prior to downstream applications. All samples were processed consistently following standardized procedures.

### Preparation of ACE

#### Conjugation of aT to the linker peptide

The conjugation was achieved through a condensation reaction, where the cl linker, PLGVR, with an NHS group at the Arg end, reacted with the amino group on the aT. The cl linker and aT were mixed in a 50:1 molar ratio and incubated at 4 °C for 12 h. Following incubation, ultracentrifugation at 7000 × *g* for 30 min was used to remove any excess linker.

#### Conjugation of aT-cl to M1ev

M1ev was pretreated with 1 mM TCEP at 37 °C for 30 min to reduce surface disulfide bonds and expose sulfhydryl groups. These reactive groups then underwent a site-specific reaction with maleimide on the cl linker. Subsequently, aT-cl was incubated with the thiol-exposed M1ev at 25 °C for 1 h to facilitate maleimide–thiol coupling. The resulting mixture was purified via ultracentrifugation to isolate the modified ACE.

### Morphology of ACE

The morphology of extracellular vesicles was visualized using a JEM-1400 transmission electron microscope (Jeol) operated at 120 kV. Nanoparticle tracking analysis (NTA, Particle Metrix) was employed to assess the size distribution and zeta potential of the samples.

### Western blot analysis

The levels of ALIX, CD63, TSG101, iNOS, aT, and non-extracellular vesicle markers (HSPA13, LDHA, and LDHB) in aT, M1ev, M1 macrophages, and ACE samples were evaluated by Western blot. Additionally, to assess therapeutic efficacy and underlying mechanisms, the expression of proteins associated with the TGF-β/Smad signaling pathway, M2 macrophage polarization, and ECM remodeling across various treatment groups were similarly evaluated, utilizing GAPDH as the internal control. Proteins extracted from the samples were separated on 4–20% SDS-PAGE gels and transferred onto polyvinylidene fluoride (PVDF) membranes using a wet transfer system. Following blocking, the membranes were incubated with appropriate primary antibodies, followed by horseradish peroxidase (HRP)-conjugated secondary antibodies (goat anti-rabbit IgG H&L and goat anti-mouse IgG H&L). Protein bands were visualized using chemiluminescence with an HRP substrate (Millipore). Protein levels were quantified by calculating grayscale values using ImageJ software.

### Colocalization of ACE

M1ev and aT were labeled with PKH26 and Alexa Fluor 488, respectively. Fluorescent signals were acquired using Nano flow cytometry (CytoFLEX Nano, Beckman Coulter).

### Proteomic and miRNA-sequencing analysis

ACE samples in three states (unconjugated with antibodies, freshly prepared, and Lyo/Reh, *n* = 1 biological replicate per group) were submitted to Personalbio for label-free quantitative proteomic analysis and miRNA sequencing. Briefly, proteins were extracted using SDT lysis buffer (4% sodium dodecyl sulfate, 100 mM Tris-HCl, pH 7.6, 0.1 M dithiothreitol) and digested with trypsin using the FASP method. Peptides were then analyzed using an Easy nLC system coupled to a Q-Exactive mass spectrometer (Thermo Fisher Scientific) via data-dependent acquisition. Raw data were processed with MaxQuant (v1.5.5.1) against the UniProt *Mus musculus* database (release 20220617) for label-free quantification (LFQ). Search parameters included up to two missed cleavages, carbamidomethyl (C) as a fixed modification, and oxidation (M) as a variable modification, with both peptide and protein false discovery rate (FDR) below 1%. Gene Ontology (GO), Kyoto Encyclopedia of Genes and Genomes (KEGG) enrichment, and protein–protein interaction network analyses were performed using the STRING database. Total RNA was extracted using TRIzol, and small RNA libraries were constructed with the NEBNext kit. The libraries were quality-assessed, quantified, and sequenced on the NovaSeq 6000 platform.

### Preparation of ACE^Plus^

DSPE-PEG-SH (10 mg mL^−1^ in ethanol) was combined with ACE (extracellular vesicle concentration: 1 mg mL^−1^) at a volume ratio of 1:100 and gently stirred at 4 °C for 20 min. The aP-cl conjugation was performed using the same approach as for aT, involving a condensation reaction in which the NHS-functionalized PLGVR linker reacted with primary amines on the aP antibody to form stable amide bonds. The cl linker and aP were incubated together following the same procedure as previously described, and excess linker was removed by centrifugation. Subsequently, aP-cl was incubated with the thiol-functionalized ACE under the same maleimide–thiol coupling conditions as previously described. The resulting product, termed ACE^Plus^, was purified by ultracentrifugation to remove unbound components.

### Colocalization of ACE^Plus^

M1ev, aT, and aP were respectively labeled with PKH26, Alexa Fluor 488, and Alexa Fluor 647. Fluorescent signals were detected by Nano flow cytometry (CytoFLEX Nano, Beckman Coulter).

### In vitro measurement of antibody release profiles

To determine the release of aT or aP, ACE or ACE^Plus^ nanomedicines were treated under varying conditions and at predetermined time intervals. Specifically, the nanomedicines were incubated with MMP2 at normal levels (3.38 ng mL^−1^) or proliferative vitreoretinopathy (PVR)-mimicking levels (406.87 ng mL^−1^, consistent with the mouse lesion site). An additional PVR-mimicking group supplemented with an MMP inhibitor was included to evaluate protease-mediated release. Following incubation, the amount of released antibody was quantified using ELISA to determine the cumulative release.

### Evaluation of TGFβ1-binding capacity

To verify the functional integrity of the released antibodies, an ELISA-based binding analysis was performed. Specifically, the aT released from the nanomedicines following incubation with proliferative vitreoretinopathy (PVR)-mimicking levels of MMP2 (406.87 ng mL^−1^) was collected. The TGFβ1-binding capacity of this released aT was then quantified and compared to that of free, unconjugated aT. For this assay, TGFβ1 was utilized at a concentration of 56.2 pg mL^−1^, a level consistent with the pathological microenvironment at the mouse lesion site. The binding analyses were conducted using three biologically independent samples.

### Construction of Transwell co-culture model

The Transwell co-culture model was constructed as follows. ARPE-19 cells were activated by stimulation with TGFβ1 (10 ng mL^−1^) for 48 h, after which the cytokine was removed by washing. The activated cells were then placed in the upper chamber of a Transwell insert. In parallel, THP-1 cells were induced to differentiate into macrophages by exposure to PMA (100 nM) for 48 h, followed by polarization into M2 macrophages using IL-4 and IL-13 (both at 20 ng mL^−1^) for an additional 48 h. After cytokine withdrawal, the resulting M2 macrophages were introduced into the lower chamber. The co-culture system was treated with either PBS or ACE and incubated at 37 °C for 24 h. Upon completion, cells from both chambers were harvested separately for RNA sequencing analysis.

### Spatiotemporal delivery

#### Prolonged vitreous retention

The A+E, AFE, or ACE formulations containing Cy7-SE labeled M1ev and Alexa Fluor 647 labeled aT were intravitreally injected into PVR model mice. In vivo imaging was conducted at defined time points using the FX Pro system (Kodak) with Carestream MI software (v5.0.7). Imaging parameters included excitation/emission wavelengths of 750/790 nm for Cy7-SE and 647/700 nm for Alexa Fluor 647, each with a 30-s exposure time. The “Green” lookup table was applied for Cy7-SE detection, and the “Red” table was used for Alexa Fluor 647 after enabling the “Select All” checkbox. Regions of interest (ROIs) in the in vivo fluorescence images were automatically identified using the “Auto-ROIs” function and quantified with the “Magic Wand” tool. Relative MFI was calculated as the MFI at observation time/MFI at day 0.

#### Selective accumulation

A+E, AFE, ACE, or ACE pretreated with different blocking strategies (PKH67-labeled M1ev) was intravitreally injected and allowed to release over a 24-h period. Specifically, ACE was incubated with the corresponding chemokine receptor antibodies (CCR1, CXCR4) at a 1:100 dilution overnight at 4 °C. Prior to enucleation, dextran was administered via tail vein injection to label retinal vasculature. Eyeballs were then collected, and retinas were flat-mounted for fluorescence imaging to quantify the selective accumulation of PKH67-labeled M1ev in lesion areas.

We employed the CRISPR system (Ubigene) to generate *Ccr1* and/or *Cxcr4* knockout models in RAW 264.7 macrophages (YC-C020, Ubigene) cultured in DMEM supplemented with 10% FBS. Gene-specific sgRNA pairs were designed to delete key coding regions within the target genes. Detailed oligonucleotide sequences, including the sgRNAs and genotyping primers used in this study, are provided in Supplementary Table 3. For transfection, cells were electroporated with ribonucleoprotein (RNP) complexes consisting of Cas9 protein and the respective sgRNA(s) at 1500 V for 10 ms (1 pulse). Following transfection, cells were subjected to limited dilution in 96-well plates to isolate single-cell clones. Knockout efficacy was confirmed by PCR amplification of the target loci followed by Sanger sequencing analysis of the monoclonal populations. To generate double-knockout cells, *Cxcr4* was sequentially knocked out in the validated *Ccr1* knockout line using identical RNP and screening procedures.

We next isolated extracellular vesicles from the aforementioned gene-edited cells via sequential differential ultracentrifugation and performed retinal flat-mount imaging to quantify the selective accumulation of PKH67-labeled M1ev in lesion areas, following the same experimental paradigm. The newly generated *Ccr1* knockout, *Cxcr4* knockout, and *Ccr1*/*Cxcr4* double-knockout RAW 264.7 cell lines in this study are available from the corresponding author upon reasonable request.

#### Controlled release

For controlled release assessment, A+E, AFE, or ACE (PKH67-labeled M1ev and Alexa Fluor 647-labeled aT) was intravitreally administered and allowed to release for 24 h. After enucleation, the eyeballs were embedded in OCT compound, rapidly frozen in liquid nitrogen for 30 s, and subsequently kept at –20 °C. Cryosections were prepared perpendicular to the cornea and parallel to the optic nerve using a Leica microtome, followed by CLSM imaging to visualize and analyze the spatiotemporal distribution of the nanomedicine. Subsequent colocalization analysis of the acquired images was performed using ImageJ software.

### Intravitreal injection

PVR mice were anesthetized by intraperitoneal injection of 0.5% pentobarbital (0.1 mL per 10 g body weight). For early-stage treatment, 10 μg of M1ev and 2 μg of aT, either alone or in combination (A+E, AFE, or ACE), were administered into the vitreous cavity. For advanced-stage intervention, a single dose containing 10 μg of M1ev, 2 μg each of aT and aP, either individually or as a combined formulation (ACE or ACE^Plus^), was administered similarly. All injections were performed using a 33-G Hamilton microinjector.

### RNA-sequencing analysis

Total RNA was extracted from mouse PVR retinal tissues and reverse-transcribed into cDNA using SuperScript III reverse transcriptase according to the manufacturer’s instructions. The resulting cDNA was fragmented with a Covaris S2 ultrasonicator, and sequencing libraries were constructed using the KAPA high-throughput preparation kit. Sequencing was performed on the BGISEQ-500 platform. Transcript abundance was quantified by the fragments per kilobase of transcript per million mapped reads (FPKM) approach. Differential gene expression was analyzed with the DESeq2 R package (v1.20.0) based on fold-change calculations. Genes with an absolute fold change ≥2 and an adjusted *P*-value < 0.05 were considered significantly differentially expressed.

### Visual cliff

The visual cliff test was performed to assess visual function using a standardized protocol. The apparatus comprised two transparent plastic enclosures. The lower box (50 × 40 × 50 cm^3^) displayed a checkerboard of black-and-white squares (2 × 2 cm^2^) covering half of its top and bottom surfaces. The upper box, positioned above it, featured a clear base and opaque black side walls, dividing the field into a “safe zone” above the patterned area and an “unsafe zone” over the transparent floor. Mice were initially placed in the safe zone and given 5 min to freely explore. Their movements were tracked, and time spent in each zone was analyzed using ANY-maze software (Stoelting Co., Wood Dale, USA). The apparatus was cleaned with water and ethanol between trials, and all behavioral assessments were conducted by a blinded operator to ensure objectivity.

#### OCT

Following treatment with PBS, aT, M1ev, A+E, AFE, or ACE, retinal scans and the anterior segment of mouse eyes were acquired by OCT imaging using an ultramicro ophthalmol imaging system (Optoprobe Science, United Kingdom).

#### ERG

Retinal function was evaluated by ERG following standard procedures. Mice were dark-adapted for 12 h under low-intensity red light before being anesthetized and undergoing pharmacological pupil dilation. After topical anesthesia of the cornea, electrodes were arranged with the active lead on the corneal surface, the reference on the cheek, and a ground electrode placed subcutaneously at the tail. Under low-light conditions, a flash stimulus was delivered every 15 s, repeated three times per eye to ensure reproducibility. ERG waveforms were captured and processed using a visual electrophysiological recording system (Optoprobe Science, UK), with data metrics, such as response amplitude, latency, and intensity extracted for analysis.

### Safety assessment in vivo

To assess the ocular safety and tolerance of the intravitreal injection of nanomedicines, intraocular pressure was determined by averaging six consecutive readings from an iCare tonometer (TONOLAB), and the anterior segment was evaluated with an ultramicro ophthalmol imaging system (Optoprobe Science, United Kingdom). In addition, aqueous humor samples were collected for ELISA analysis of inflammatory cytokines to monitor potential intraocular inflammatory responses.

### Statistics and reproducibility

Statistical analyses were conducted using GraphPad Prism 9.0.0 and Origin 2022. The CIBERSORTx analytical tool (https://cibersortx.stanford.edu/) was employed to evaluate immune cell infiltration profiles. The online platform OmicShare tools (https://www.omicshare.com/tools) was specifically utilized to perform correlation coefficient analyses. Additionally, this platform was used for data visualization, including the generation of volcano plots for DEGs, heatmaps, immune cell proportions, and Sankey diagrams. Quantitative data are presented as mean ± s.d. or median (25th–75th quartiles) for non-normally distributed data. A two-tailed unpaired Student’s *t* test or Mann–Whitney *U* test was used to compare two groups, while one-way analysis of variance (ANOVA) was applied for comparisons among multiple groups. A two-sided *p* < 0.05 was considered statistically significant. Exact *p-*values are reported (unless *p* < 0.0001), and 95% confidence intervals for the difference in means are provided for key outcome measures. Significance levels were denoted as *p < 0.05, **p < 0.01, ***p < 0.001, and ****p < 0.0001 (ns, not significant). The correlations were analyzed by Spearman’s rank correlation or Pearson correlation analysis.

Experiments were replicated multiple times with similar results. Specifically, in vitro and ex vivo assays were repeated three times. For in vivo animal evaluations and clinical sample analyses, representative images were selected from biologically independent animals or patients (sample sizes are detailed in the respective figure legends), with similar features observed within the same group.

### Reporting summary

Further information on research design is available in the Nature Portfolio Reporting Summary linked to this article.

## Supplementary information

Supplementary Information

Reporting Summary

Transparent Peer Review File

## Source data

Source Data

## Data Availability

The main data supporting the results in this study are available within the paper and its Supplementary Information. The RNA-sequencing data are available from the NCBI BioProject via the accession code PRJNA1415138. The mass spectrometry and proteomics data are available from the ProteomeXchange Consortium (through the iProX partner repository) via the dataset identifier PXD073679. Source data are provided with this paper and are also available in Figshare (https://doi.org/10.6084/m9.figshare.33080657). Additionally, the publicly available RNA-sequencing dataset analyzed in this study can be found in the NCBI BioProject under the accession code PRJNA744251. Source data are provided with this paper.
