# Supplementary material for: Therapeutic modulation of the vitreoretinal fibrosis microenvironment in female mice using engineered macrophage-derived extracellular vesicles
Source: Nat Commun. 2026 Aug 25;17:10180. doi: 10.1038/s41467-026-76550-z (PMC13612821; doi:10.1038/s41467-026-76550-z)
Supplement: Supplementary file 1 [file 41467_2026_76550_MOESM1_ESM.pdf]

Supplementary information

Therapeutic modulation of the vitreoretinal fibrosis microenvironment in female mice using engineered macrophage-derived extracellular vesicles

Yingjie Wang[1,2], Fuxiao Luan[1], Jiawei Zhao[1,2], Peilin Guo[2,3], Shuang Wang[2,3], Jinghui Wang[4], Dongfang Wang[5], Jing Hou[6,7], Xiaofeng Hu[1], Li Chen[1], Xusheng Cao[8], Guanghui Ma[2,3,*], Yong Tao[1,9,10,11,*], Ying Tian[1,*], and Wei Wei[2,3,*].

[1]Department of Ophthalmology, Beijing Chaoyang Hospital, Capital Medical University; Beijing 100020, P.R. China.

[2]State Key Laboratory of Biopharmaceutical Preparation and Delivery, Institute of Process Engineering, Chinese Academy of Sciences; Beijing 100190, P.R. China.

[3]School of Chemical Engineering, University of Chinese Academy of Sciences; Beijing 100049, P.R. China.

[4]Department of Gastroenterology, Tongren Hospital, Shanghai Jiao Tong University School of Medicine; Shanghai 200336, P.R. China.

[5]Biomedical Pioneering Innovation Center (BIOPIC), Peking University; Beijing 100871, P.R. China.

[6]Department of Ophthalmology, Peking University People's Hospital; Beijing 100044, P.R. China.

[7]Beijing Key Laboratory of Ocular Disease and Optometry Science, Peking University People's Hospital; Beijing 100044, P.R. China.

[8]Beijing Tongren Eye Center, Beijing Tongren Hospital, Capital Medical University, Beijing Key Laboratory of Ophthalmology and Visual Sciences; Beijing 100730, P.R. China.

[9]National Engineering Research Center for Ophthalmology; Beijing, P.R. China.

[10]Engineering Research Center of Ophthalmic Equipment and Materials, Ministry of Education; Beijing, P.R. China.

[11]Chinese Institutes for Medical Research, 10 Xitoutiao, Youanmen Wai, Fengtai District, Beijing 100069, P.R. China.

*Correspondence: weiwei@ipe.ac.cn (W.W.), tianying@mail.ccmu.edu.cn (Y.Tian), taoyong@mail.ccmu.edu.cn (Y.Tao), ghma@ipe.ac.cn (G.M.).

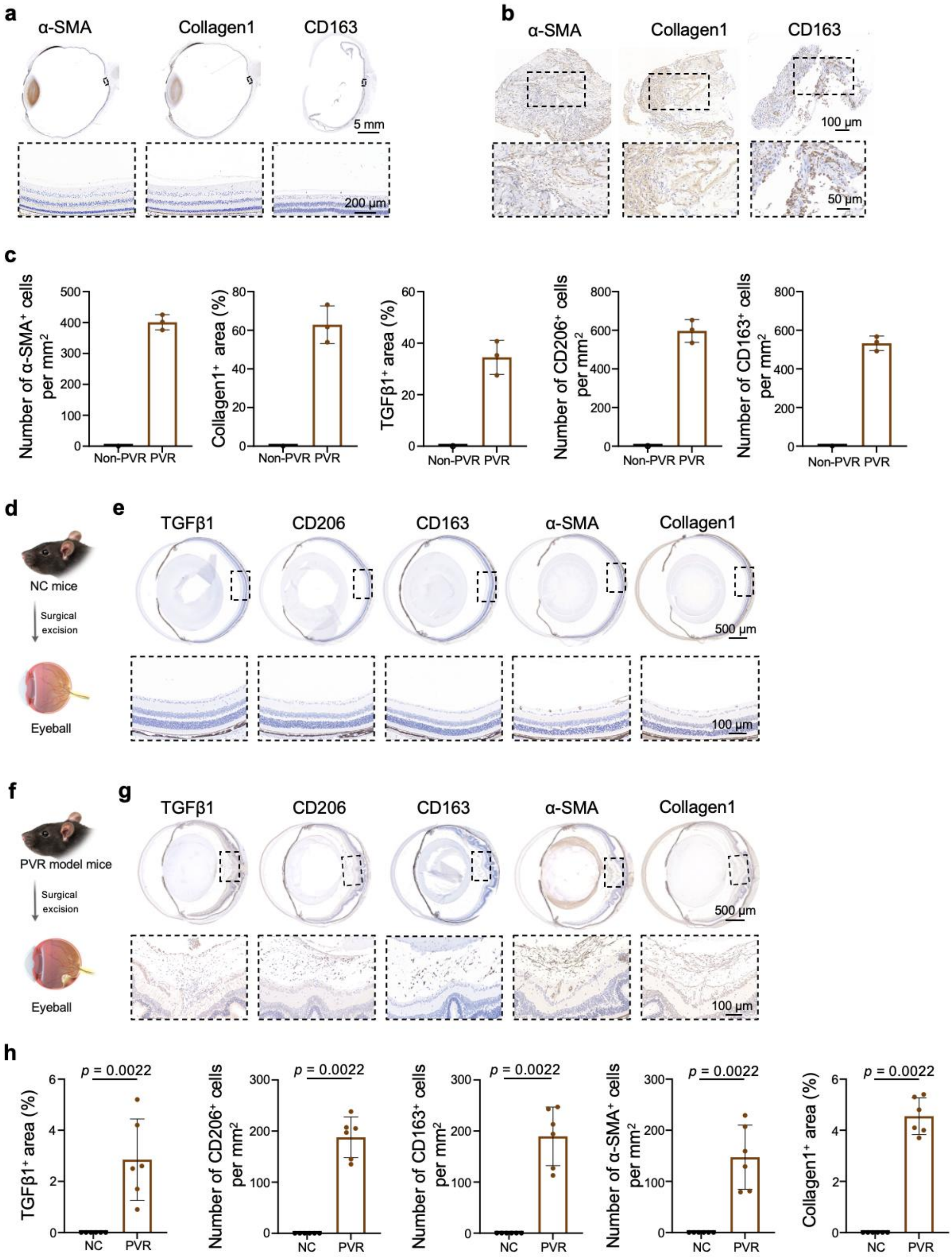

Supplementary Fig. 1 | Additional verifications and characterizations of clinical samples and PVR model mice, related to Fig. 1.

a Immunohistochemical (IHC) staining of sectioned eyeball from a non-PVR donor, showing the expression of α-SMA, Collagen1, and CD163. The outlined areas are magnified in the bottom sub-panels.

b IHC staining of sectioned PVR membrane from a PVR patient, showing the expression of α-SMA, Collagen1, and CD163. The outlined areas are magnified in the bottom sub-panels.

c Quantitative analysis of the transforming growth factor β1 (TGFβ1)-positive area, the number of CD206-positive cells, the number of CD163-positive cells, the number of α-SMA-positive cells, and the Collagen1-positive area on the PVR membrane in the vitreous cavity (*n* = 1 eyeball from a non-PVR donor, *n* = 3 PVR membranes from PVR patients).
d Schematic illustration of the eyeball from non-model control (NC) mice.
e IHC staining of sectioned eyeballs from NC mice, showing the expression of TGFβ1, CD206, CD163, α-SMA, and Collagen1. The outlined areas are magnified in the bottom sub-panels.
f Schematic illustration of the eyeball from PVR model mice.
g IHC staining of sectioned eyeballs from PVR model mice, showing the expression of TGFβ1, CD206, CD163, α-SMA, and Collagen1. The outlined areas are magnified in the bottom sub-panels.
h Quantitative analysis of the TGFβ1-positive area, the number of CD206-positive cells, the number of CD163-positive cells, the number of α-SMA-positive cells, and the Collagen1-positive area on the PVR membrane in the vitreous cavity (*n* = 6, 1 eye from each of 6 mice).
Data in **c** and **h** are presented as mean ± s.d. and data in **h** were compared using Mann-Whitney U test.

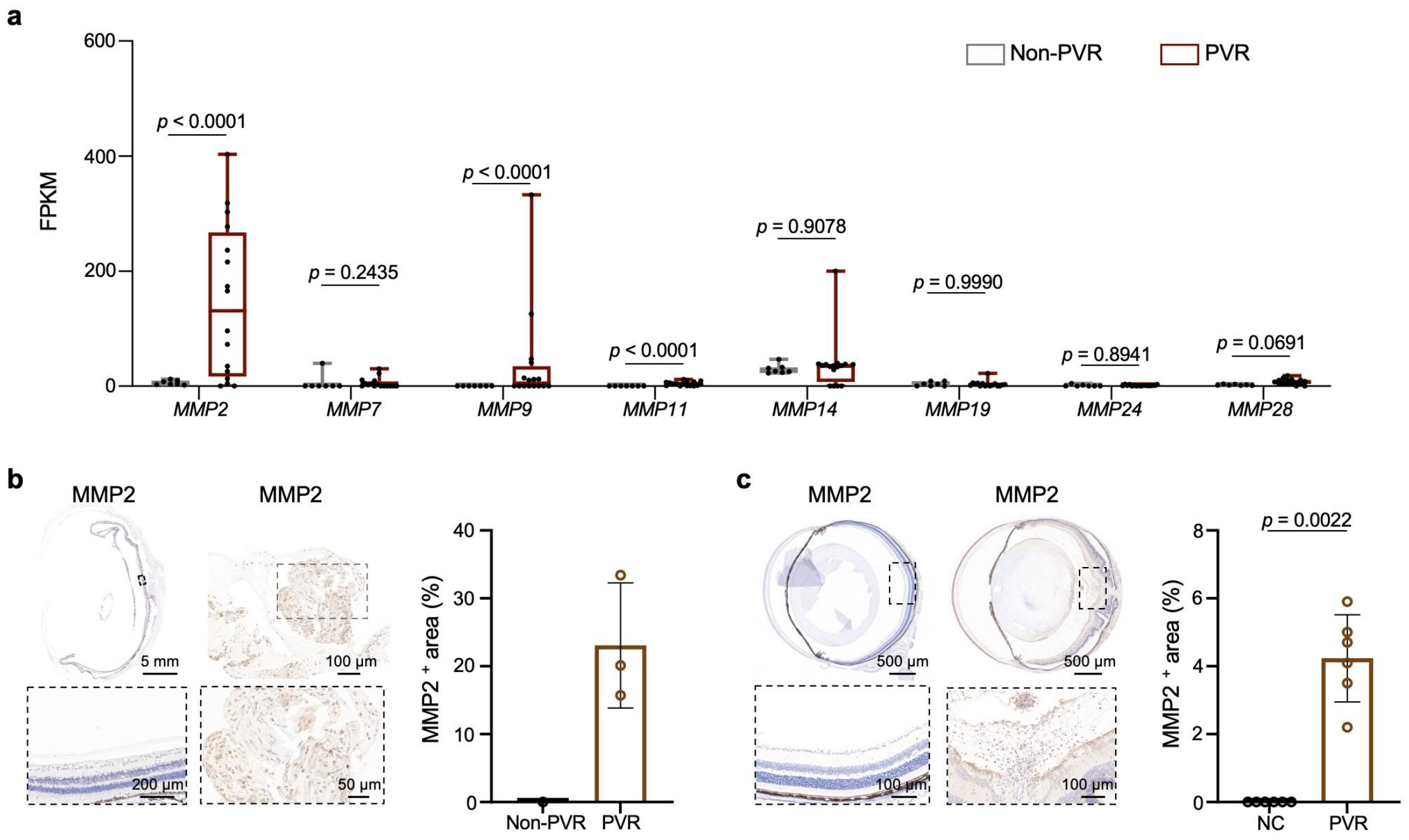

Supplementary Fig. 2 | Additional depiction of the vitreoretinal fibrosis microenvironment, related to Fig. 1.

a Box plot showing the expression of matrix metalloproteinase (MMP)-related differentially expressed genes (DEGs) in PVR samples (n = 16) compared to non-PVR samples (n = 7) from the integrated dataset.

b Left: IHC staining of a sectioned eyeball from a non-PVR donor and PVR membrane from PVR patients, showing the expression of MMP2. The outlined areas are magnified in the bottom sub-panels. Right: quantitative analysis of the MMP2-positive area on the PVR membrane (non-PVR donor: n = 1; PVR patients: n = 3).

c Left: IHC staining of sectioned eyeballs from NC and PVR model mice, showing the expression of MMP2. The outlined areas are magnified in the bottom sub-panels. Right: quantitative analysis of the MMP2-positive area on the PVR membrane in the vitreous cavity (n = 6, 1 eye from each of 6 mice).

Data in **a** were compared using two-sided Wald test (DESeq2) with Benjamini-Hochberg adjustment. Data in **b** and **c** are presented as mean ± s.d.. Data in **c** were compared using Mann-Whitney U test.

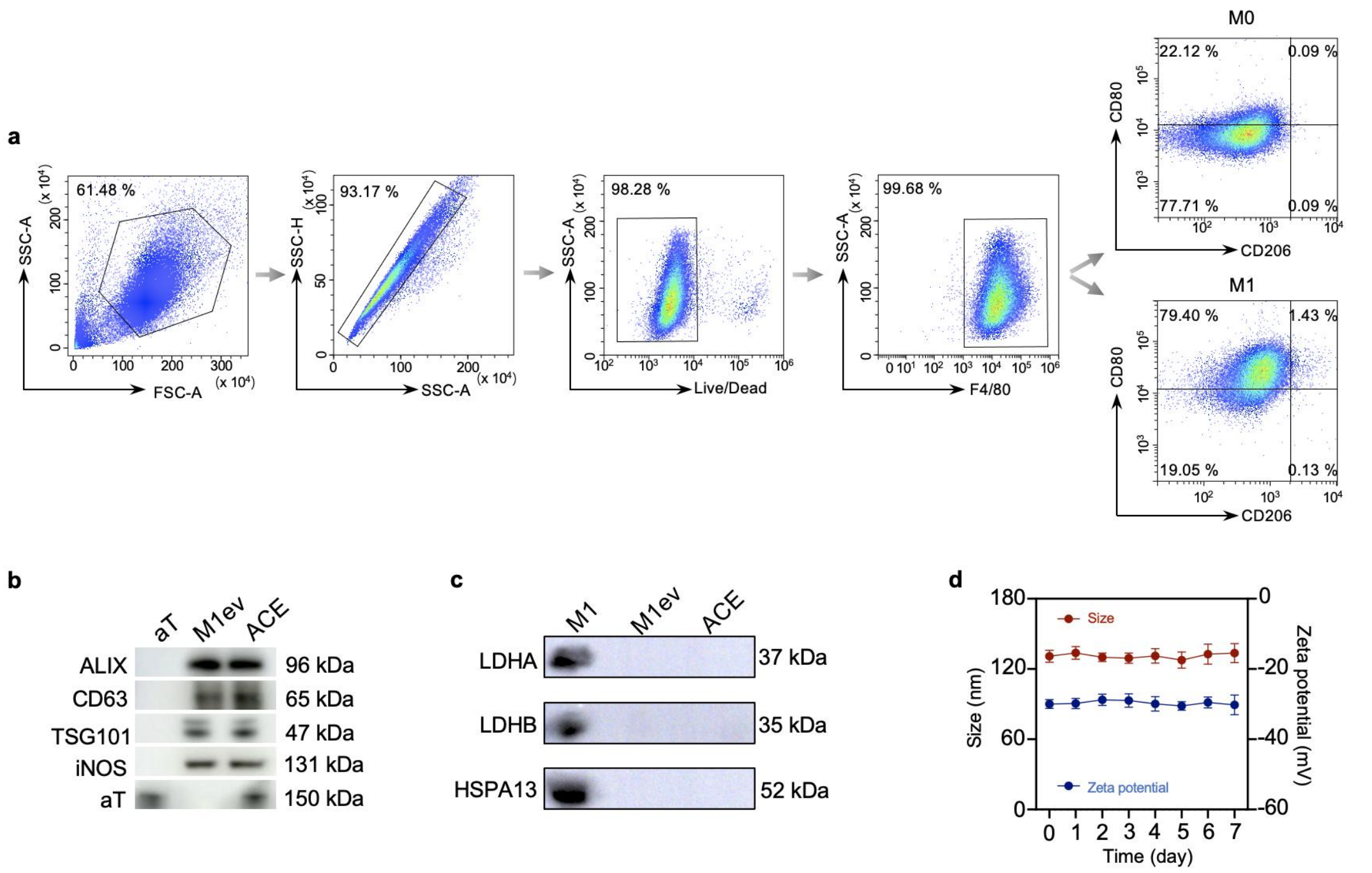

Supplementary Fig. 3 | Additional characterization of aT-cl-M1ev (ACE), related to Fig. 1.

a Flow cytometry gating strategy and phenotypic characterization of M1 macrophages.
b Western blot analysis of ACE. ALIX, CD63, and TSG101 are extracellular vesicle markers. iNOS is a specific protein on M1ev. aT is the antibody conjugated to the M1ev.
c Western blot analysis of the expression of LDHA, LDHB, and HSPA13 in parent M1 macrophages, M1ev, and ACE.
d Nanoparticle tracking analysis, showing particle size and zeta potential of ACE before and after storage in PBS at 4 °C for 7 days (*n* = 3 biologically independent experiments). Data in **d** are presented as the mean ± s.d..

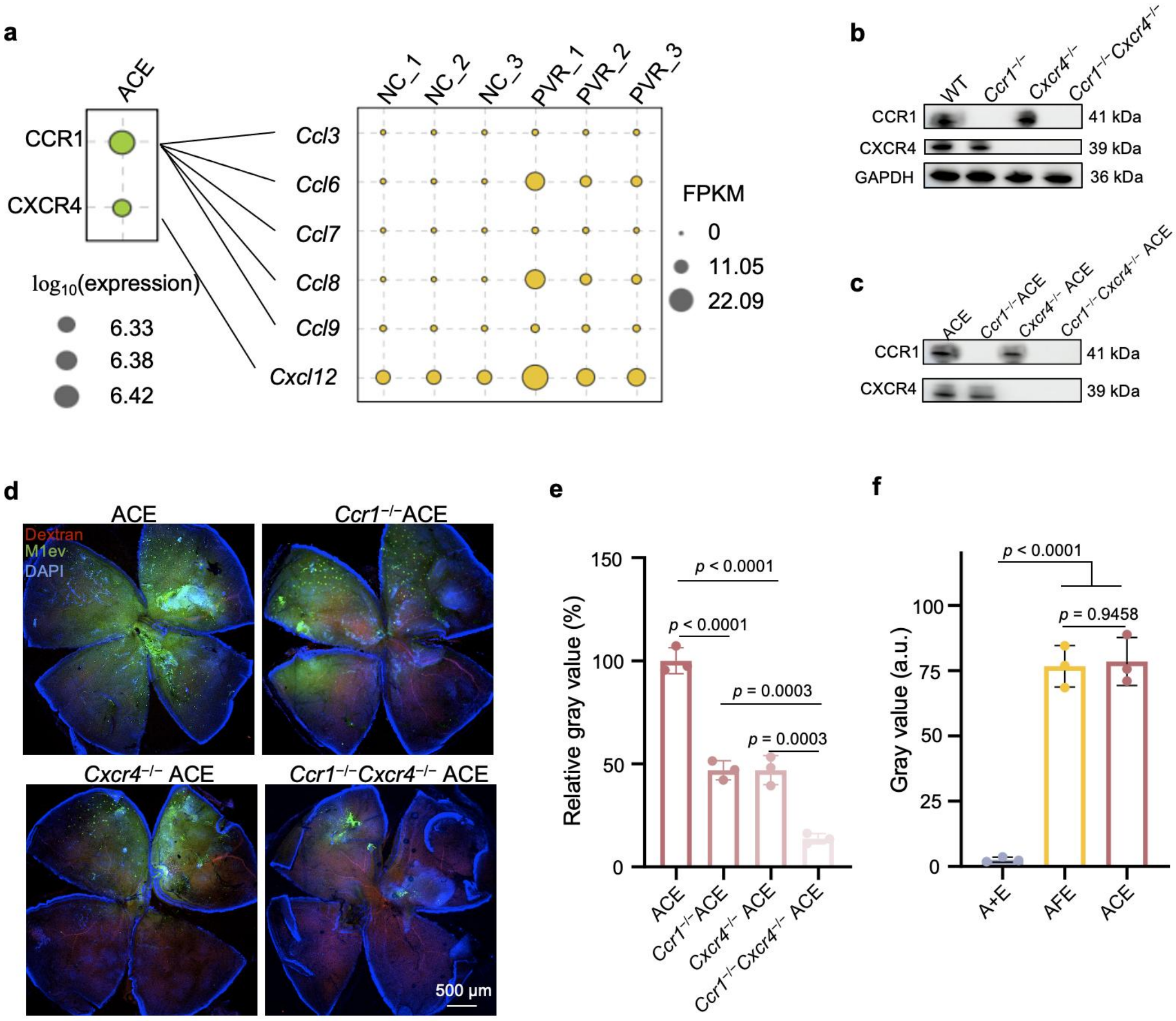

Supplementary Fig. 4 | Additional characterization of the selective accumulation of ACE, related to Fig. 2f–h.

a Chemokine receptors expressed on ACE and their corresponding ligands in the vitreoretinal fibrosis environment, related to Fig. 2g.

b Western blot analysis of the expression of CCR1 and CXCR4 in M1 macrophages, including wild-type (WT), *Ccr1*$^{-/-}$, *Cxcr4*$^{-/-}$, and double-knockout (*Ccr1*$^{-/-}$*Cxcr4*$^{-/-}$) groups.

c Western blot analysis of the expression of CCR1 and CXCR4 in ACE, including ACE, $^{Ccr1-/-}$ACE, $^{Cxcr4-/-}$ACE, and $^{Ccr1-/-Cxcr4-/-}$ACE groups.

d Representative images of the accumulation of M1ev in flat-mounted retinas of PVR model mice on day 1 after indicated treatment ($n = 3$, 1 eye from each of 3 mice; green, PKH67-labelled M1ev; red, Dextran-labelled retinal vessels; blue, DAPI-labelled cell nuclei). The images are presented at the same magnification.

e Quantification of the accumulation of M1ev in flat-mounted retinas of PVR model mice.

f Quantification of aT at the lesion site ($n = 3$, 1 eye from each of 3 mice), related to Fig. 2h.

Data in **e** and **f** are presented as mean ± s.d. and were compared using one-way ANOVA.

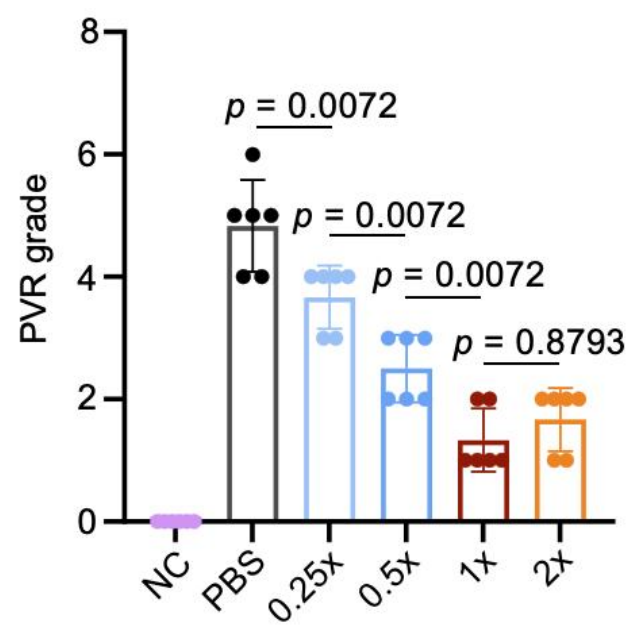

Supplementary Fig. 5 | A dose escalation study of ACE in gas- and cell-induced PVR model mice, related to Figs. 3 and 4.

Quantitative analysis of the PVR grade; Supplementary Table 2 for grade criteria (*n* = 6, 1 eye from each of 6 mice). The PVR model mice were divided into six groups, including NC, PBS, 0.25x, 0.5x, 1x, and 2x ACE groups. 1x ACE group contained 2 µg aT and 10 µg M1ev.

Data are presented as mean ± s.d. and were compared using one-way ANOVA.

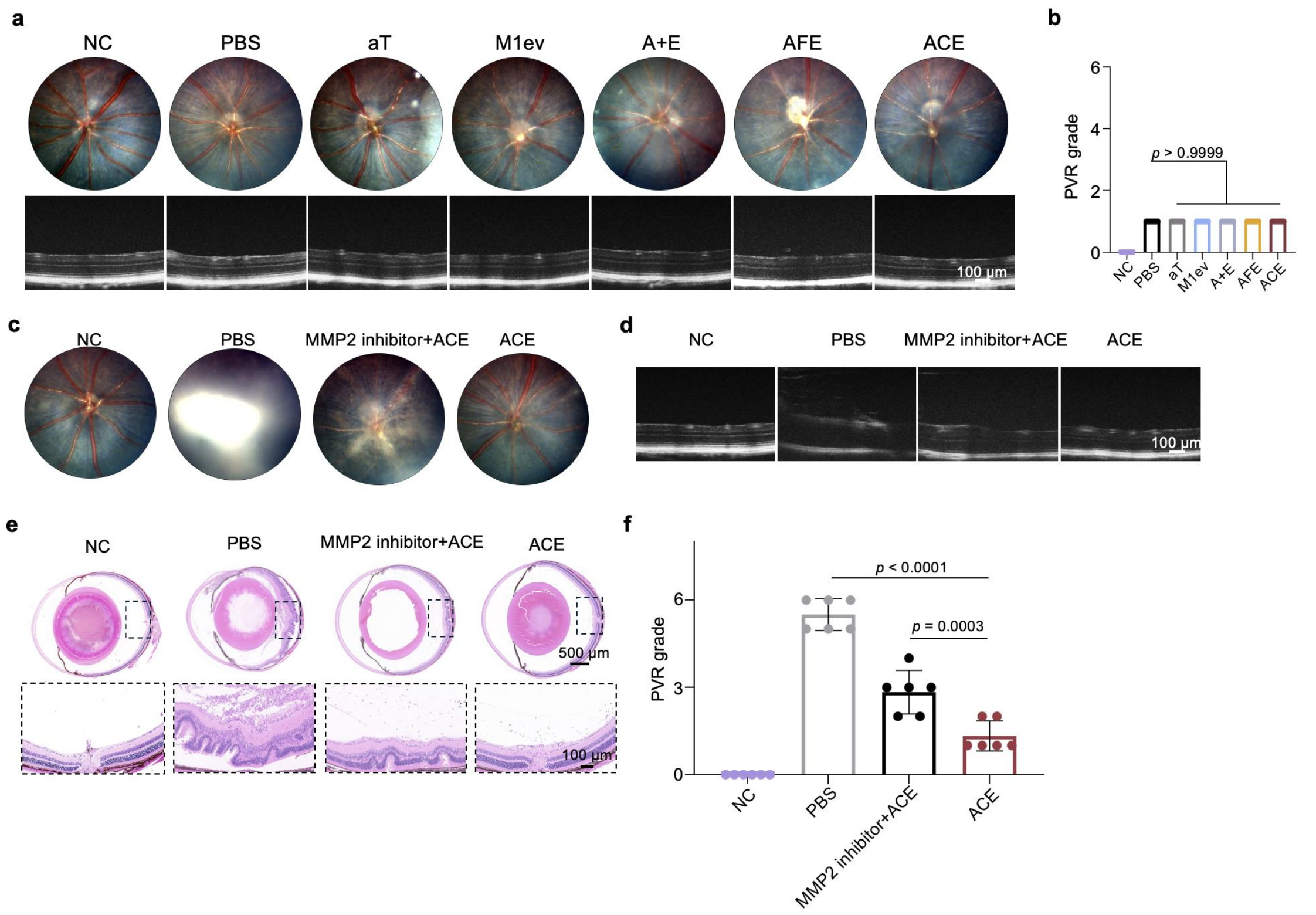

Supplementary Fig. 6 | Additional anti-fibrotic efficacy of ACE, related to Fig. 3.

a Representative color fundus photography (CFP) (top) and OCT (bottom) images of NC mice and PVR model mice, assessing PVR severity at the time of treatment initiation. The images are presented at the same magnification.

b Quantitative analysis of the PVR grade, performed based on clinical assessment, CFP, and OCT; Supplementary Table 2 for grade criteria ($n = 6$, 1 eye from each of 6 mice).

c, **d** Representative CFP (c) and OCT (d) images of NC mice and PVR model mice, assessing the role of MMP2-mediated linker cleavage *in vivo* on day 28 after intravitreal injection of indicated treatments. The images are presented at the same magnification.

e Representative hematoxylin and eosin (H&E) staining of retinal slices from PVR model mice of the indicated treatment groups on day 28. The images are presented at the same magnification. The outlined areas are magnified in the bottom sub-panels.

f Quantitative analysis of the PVR grade, performed based on clinical assessment, CFP images, OCT images, and H&E staining; Supplementary Table 2 for grade criteria ($n = 6$, 1 eye from each of 6 mice).

Data in **b** and **f** are presented as mean ± s.d. and were compared using one-way ANOVA.

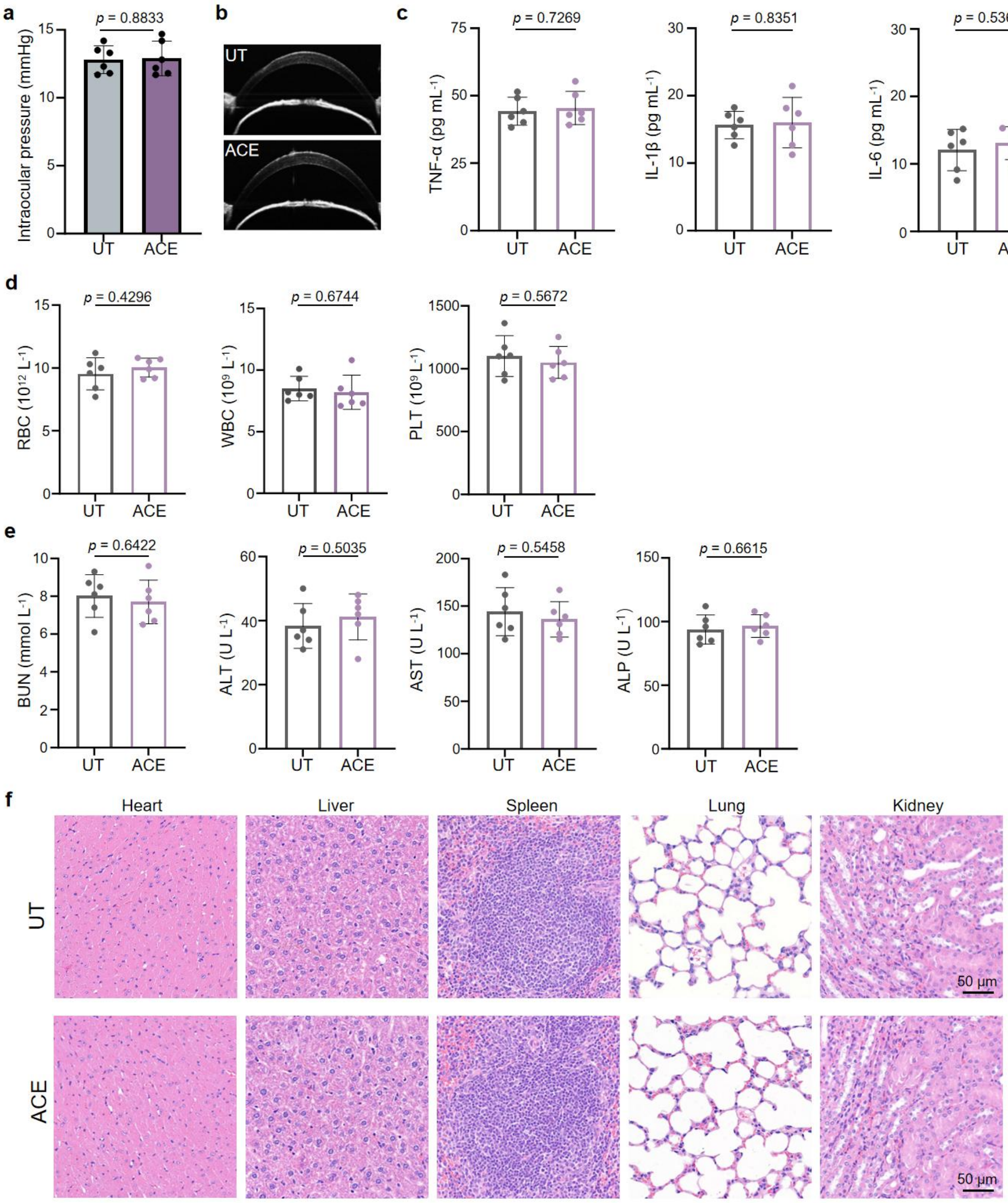

Supplementary Fig. 7 | Biosafety evaluation of ACE in C57BL/6J mice, related to Fig. 3.

a Intraocular pressure in untreated (UT) and ACE-treated C57BL/6J mice (n = 6, 1 eye from each of 6 mice).

b Anterior segment morphology of UT and ACE-treated C57BL/6J mice.

c Quantification of inflammatory cytokines (TNF-α, IL-1β, and IL-6) concentrations in the aqueous humor of UT or ACE-treated C57BL/6J mice by ELISA.

d Hematological parameters including red blood cell (RBC), white blood cell (WBC), and platelet (PLT) levels (n = 6 mice).

e Serum biochemical indices, including blood urea nitrogen (BUN), alanine aminotransferase (ALT), aspartate aminotransferase (AST), and alkaline phosphatase

(ALP) (n = 6 mice).

f Representative H&E staining images of the heart, liver, spleen, lung, and kidney tissues.

Data in **a** and **c**–**e** are presented as mean ± s.d. and were compared using unpaired two-tailed Student's *t*-test.

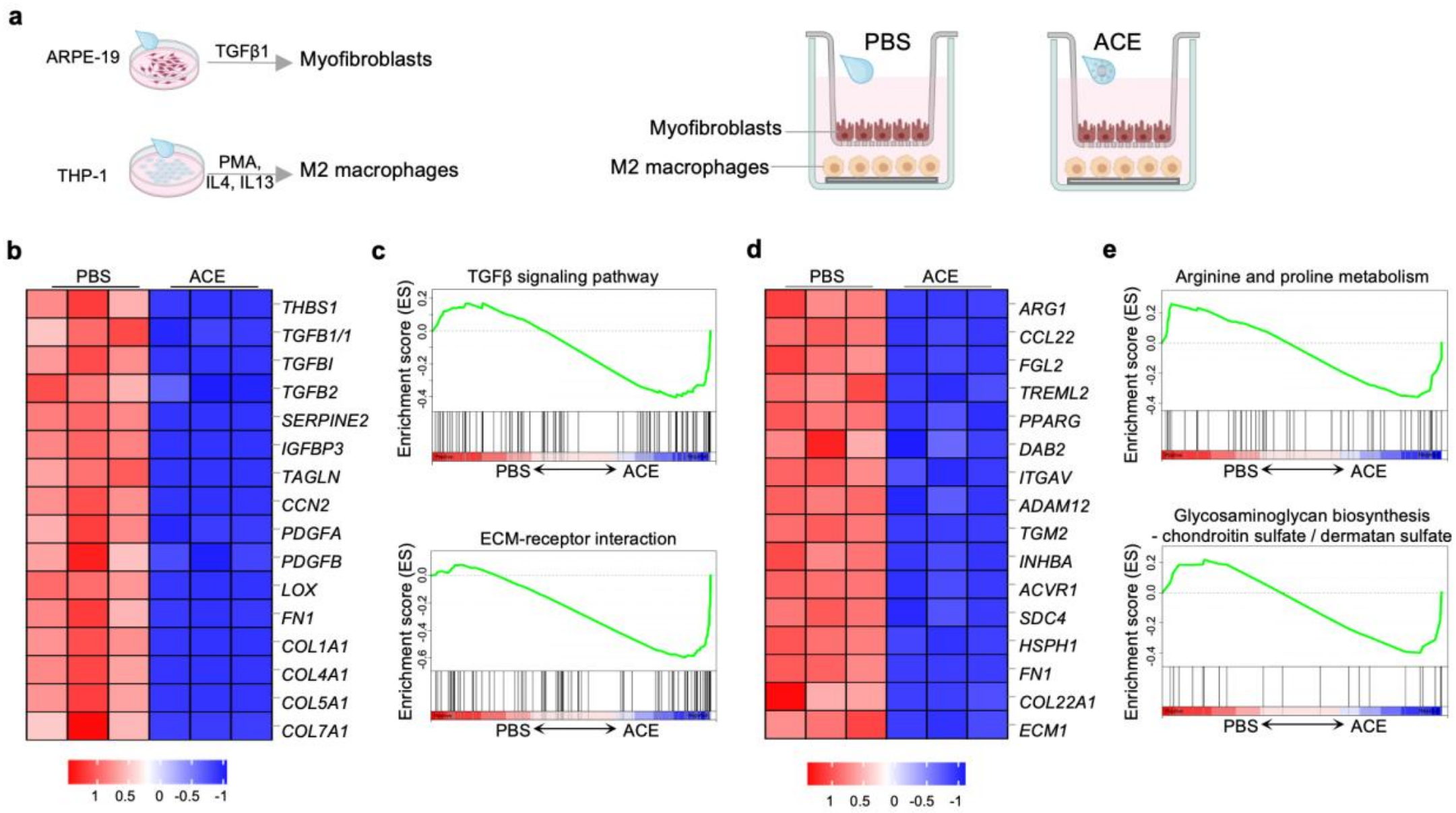

Supplementary Fig. 8 | Additional characterization of ACE-induced therapeutic modulation of the vitreoretinal fibrosis microenvironment in a Transwell^{TM} co-culture model, related to Fig. 4.

a Schematic of the experimental design for evaluating ACE-mediated therapeutic modulation of the vitreoretinal fibrosis microenvironment in an *in vitro* co-culture model. In this iterative Transwell^{TM} co-culture model, human retinal pigment epithelial cells (ARPE-19) were activated into myofibroblasts via TGFβ1 stimulation and subsequently seeded in the upper chamber. Concurrently, human monocytic leukemia cells (THP-1) were sequentially treated with phorbol 12-myristate 13-acetate (PMA), IL-4, and IL-13 to promote M2 macrophage polarization and seeded in the lower chamber. This co-culture model simulated the cellular interactions in the vitreoretinal fibrosis microenvironment. Created in BioRender. Tian, Y. (2026) https://BioRender.com/y7g38u2.

b Transcriptome analysis of myofibroblasts (upper layer) upon treatment with PBS or ACE.

c Gene set enrichment analysis (GSEA) of gene sets with expression profiles that differed between ACE-treated and PBS-treated myofibroblasts.

d Transcriptome analysis of M2 macrophages (lower layer) upon treatment with PBS or ACE.

e GSEA with expression profiles that differed between ACE-treated and PBS-treated M2 macrophages.

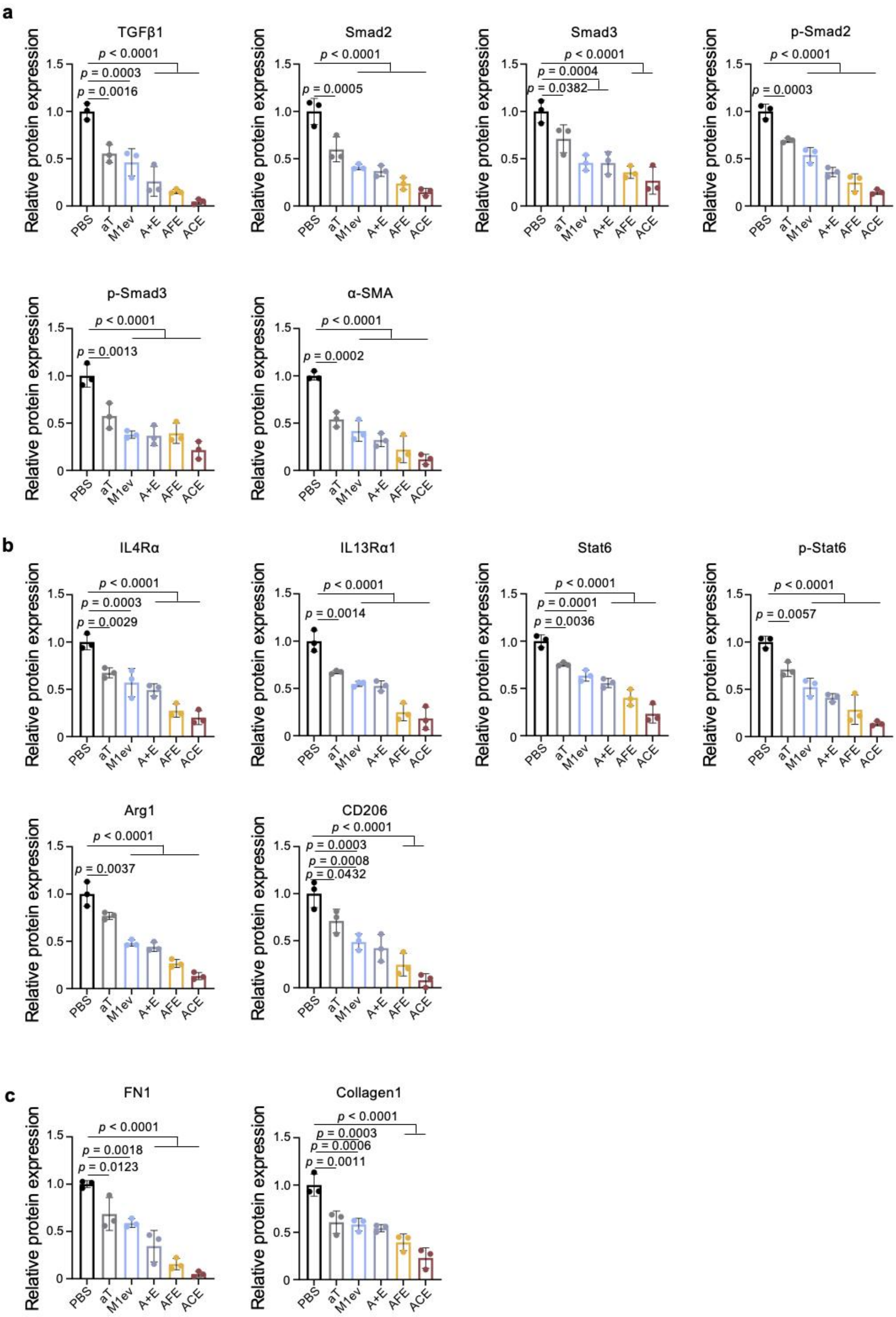

Supplementary Fig. 9 | Quantitative analysis related to Fig. 4e–g.

a Quantitative analysis of the expression of the proteins in retina from PVR model mice after intravitreal injection of indicated treatments, related to Fig. 4e (*n* = 3 biologically independent samples).

b Quantitative analysis of the expression of the proteins in retina from PVR model mice after intravitreal injection of indicated treatments, related to Fig. 4f (*n* = 3 biologically independent samples).
c Quantitative analysis of the expression of the proteins in retina from PVR model mice after intravitreal injection of indicated treatments, related to Fig. 4g (*n* = 3 biologically independent samples).
Data in **a**–**c** are presented as mean ± s.d. and were compared using one-way ANOVA.

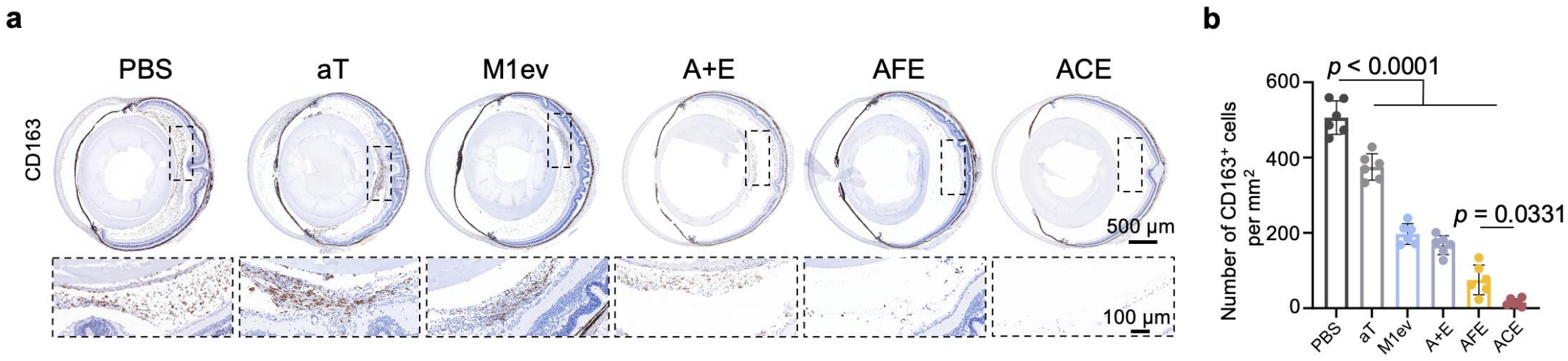

Supplementary Fig. 10 | Additional evaluation of therapeutic modulation of the vitreoretinal fibrosis microenvironment after ACE treatment, related to Fig. 4i.

a IHC staining showing the expression of CD163 in ocular sections of PVR model mice of the indicated treatment groups on day 28. The images are presented at the same magnification. The outlined areas are magnified in the bottom sub-panels.

b Quantitative analysis of the number of CD163-positive cells on the PVR membrane in the vitreous cavity (*n* = 6, 1 eye from each of 6 mice).

Data in **b** are presented as mean ± s.d. and were compared using one-way ANOVA.

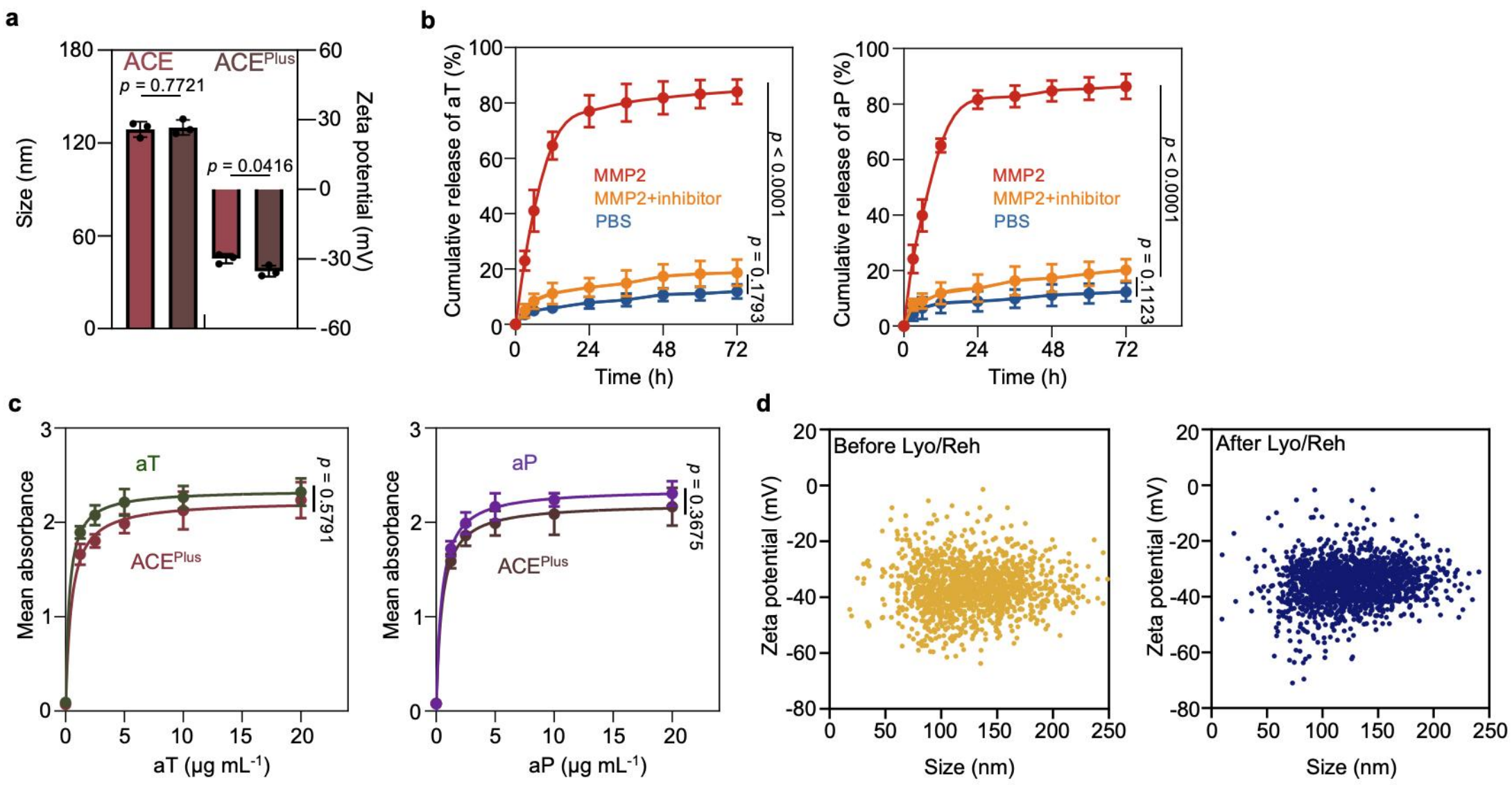

Supplementary Fig. 11 | Additional characterization of aTP-cl-M1ev (ACE^{Plus}), related to Fig. 5.

a Nanoparticle tracking analysis, showing particle size and zeta potential of ACE and ACE^{Plus} ($n = 3$ biologically independent samples).

b Enzyme-linked immunosorbent assay (ELISA)-based quantification of aT and aP levels under different conditions, showing ACE^{Plus}'s release of aT and aP upon MMP2 exposure ($n = 3$ biologically independent samples).

c ELISA-based binding analysis demonstrated that the neutralizing capacity of ACE^{Plus} (the released aT and aP) was comparable to that of free, unconjugated aT and aP ($n = 3$ biologically independent samples).

d Particle size and zeta potential of lyophilized ACE^{Plus} after rehydration. Images show the particles before (left) and after (right) Lyo/Reh.

Data in **a**–**c** are presented as mean ± s.d.. Data in **a** and **c** were compared using unpaired two-tailed Student's *t*-test. Data in **b** were compared using one-way ANOVA.

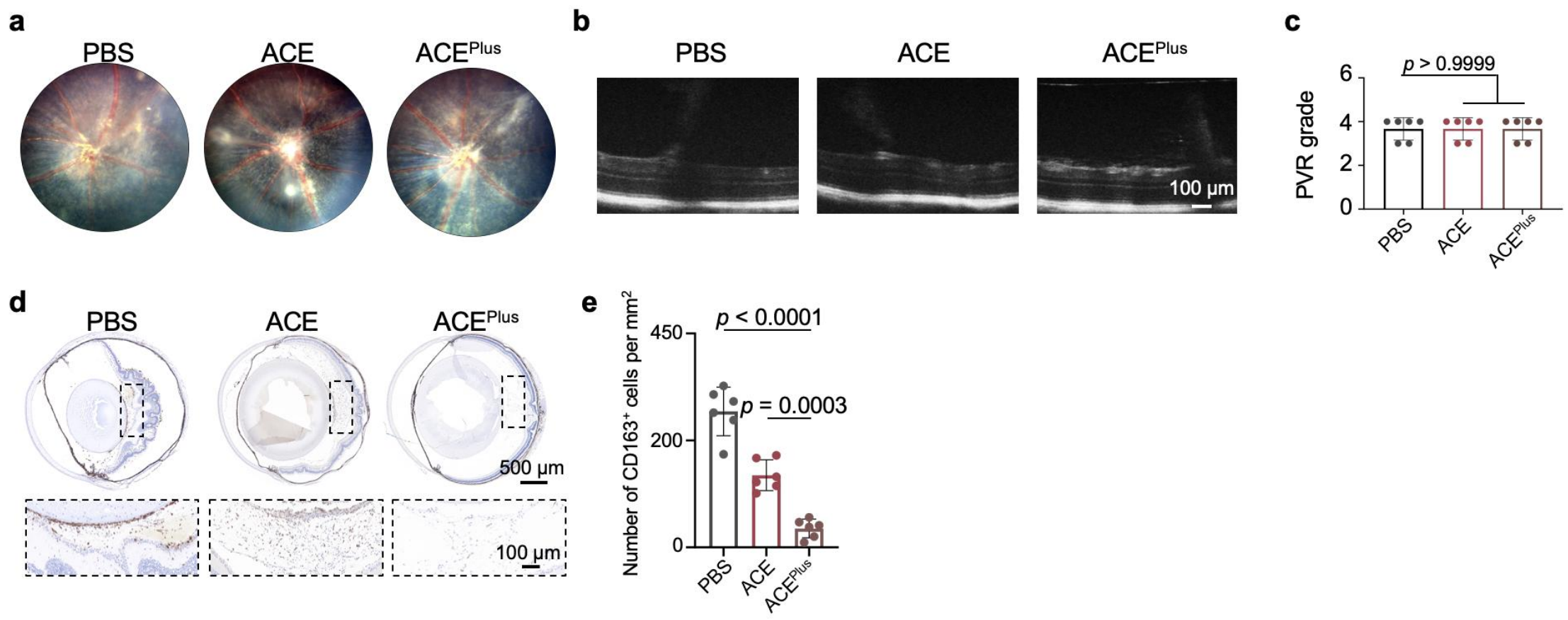

Supplementary Fig. 12 | Additional anti-fibrotic efficacy of ACE^{Plus}, related to Fig. 5.

a, **b** Representative CFP (a) and OCT images (b) assessing PVR severity in advanced-stage model mice at the time of treatment initiation. The images are presented at the same magnification.

c Quantitative analysis of the PVR grade, performed based on clinical assessment, CFP, and OCT; Supplementary Table 2 for grade criteria (n = 6, 1 eye from each of 6 mice).

d IHC staining showing CD163 expression in ocular sections from advanced-stage PVR model mice on day 28 following the indicated treatments. The images are presented at the same magnification.

e Quantitative analysis of the number of CD163-positive cells on the PVR membrane in the vitreous cavity (n = 6, 1 eye from each of 6 mice).

Data in **c** and **e** are presented as mean ± s.d. and were compared using one-way ANOVA.

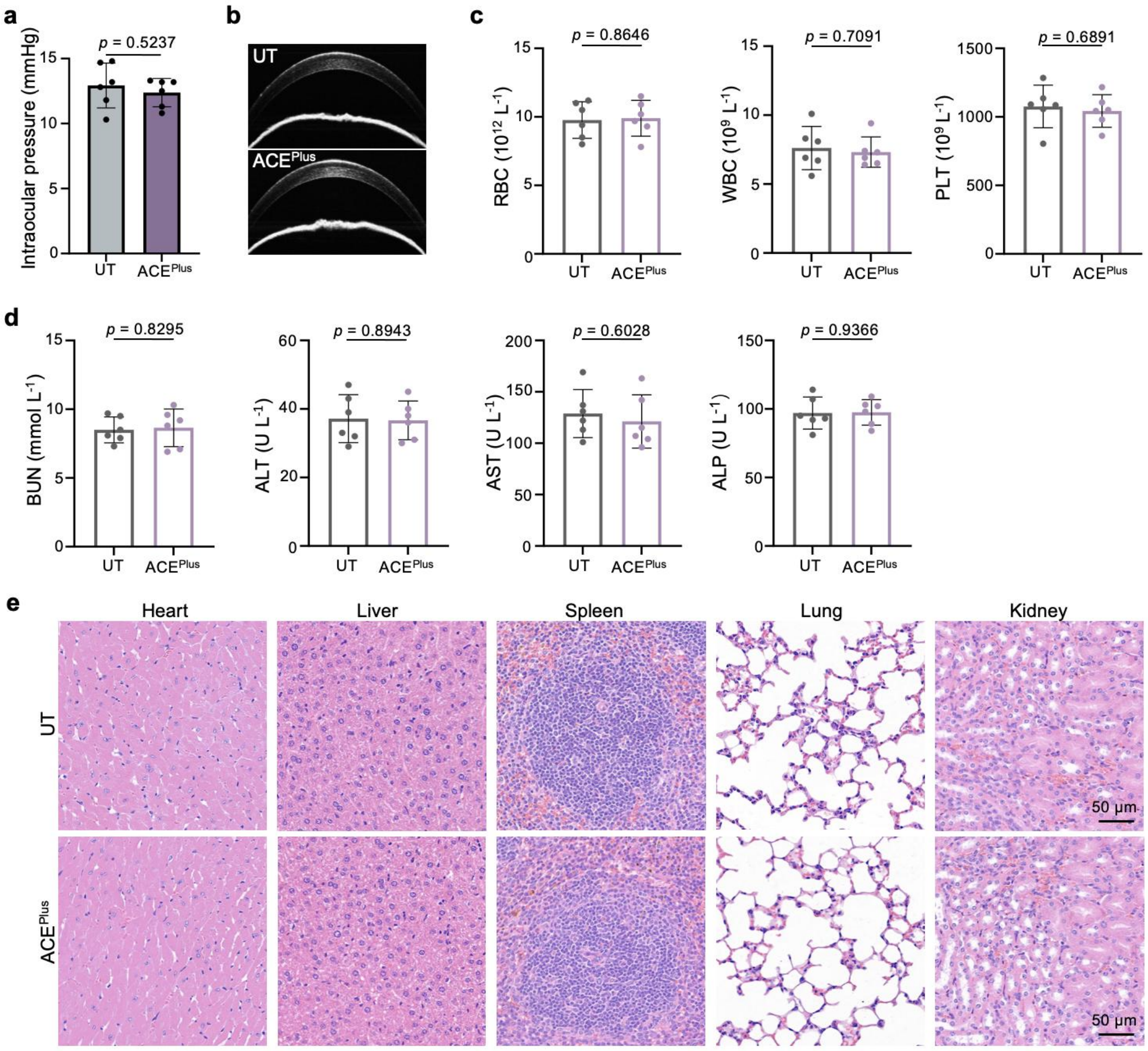

Supplementary Fig. 13 | Biosafety evaluation of ACE^{Plus} in C57BL/6J mice, related to Fig. 5.

a Intraocular pressure after UT or treatment with ACE^{Plus} in C57BL/6J mice (n = 6, 1 eye from each of 6 mice).

b Anterior segment morphology after UT or treatment with ACE^{Plus} in C57BL/6J mice.

c Hematological parameters including RBC, WBC and PLT levels (n = 6 mice).

d Serum biochemical indices, including BUN, ALT, AST, and ALP (n = 6 mice).

e Representative H&E staining images of the heart, liver, spleen, lung, and kidney tissues.

Data in **a**, **c**, **d** are presented as the mean ± s.d.. These data were compared using unpaired two-tailed Student's t-test.

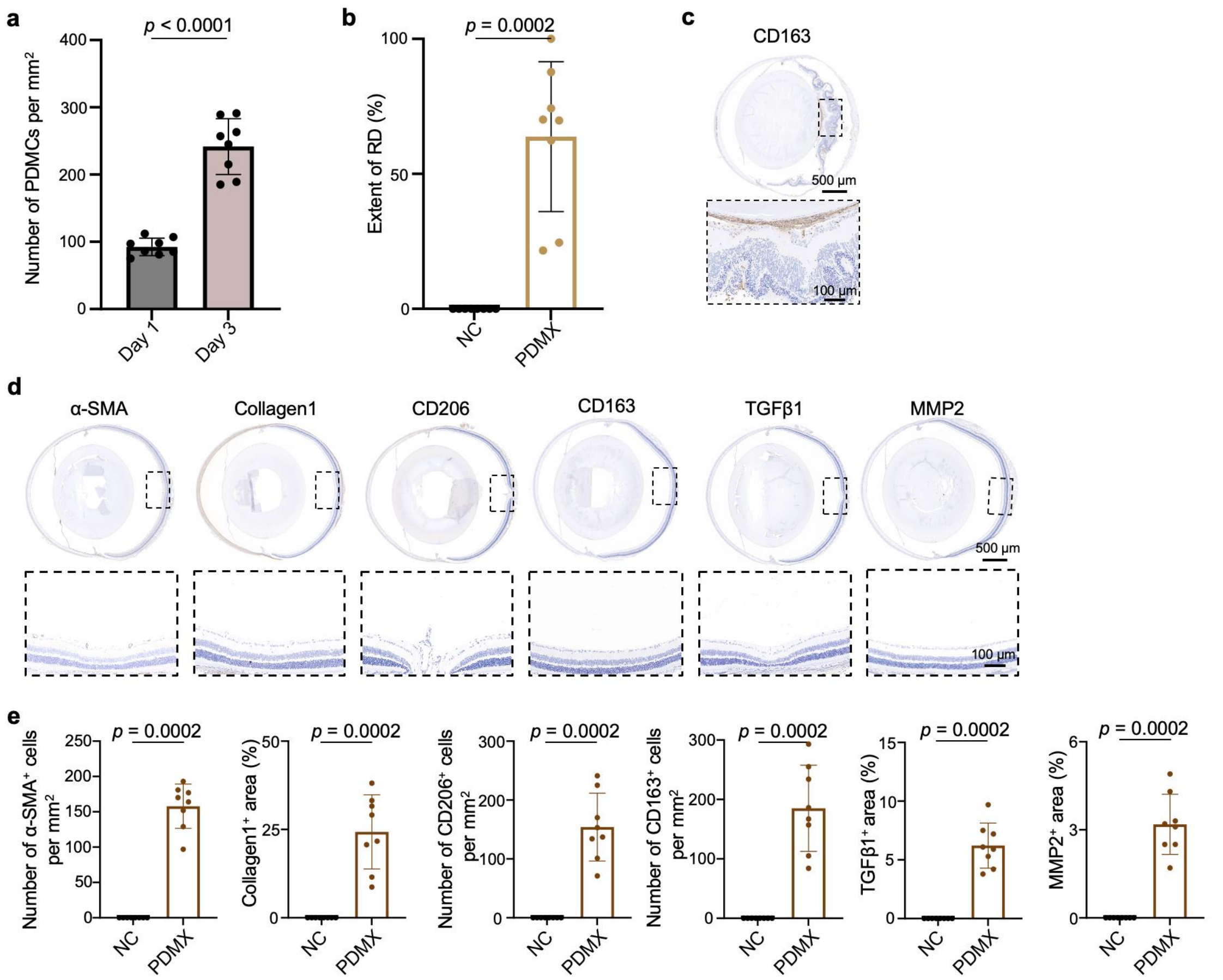

Supplementary Fig. 14 | Additional characterizations of PDMX, related to Fig. 6.

a Quantitative analysis of the number of patient-derived PVR membrane cells (PDMCs) on day 1 and day 3 after being cultured *in vitro* (n = 8 biologically independent experiments).

b Quantitative analysis of the extent of retinal detachment (RD) in NC and PDMX model mice (n = 8, 1 eye from each of 8 mice).

c IHC staining showing the expression of CD163 in ocular sections of NC and PDMX mice on day 28. The outlined area is magnified in the bottom sub-panel.

d IHC staining of sectioned eyeballs from NC mice, showing the expression of α-SMA, Collagen1, CD206, CD163, TGFβ1, and MMP2. The outlined areas are magnified in the bottom sub-panels.

e Quantitative analysis of the number of α-SMA-positive cells, the Collagen1-positive area, the number of CD206-positive cells, the number of CD163-positive cells, the TGFβ1-positive area, and the MMP2-positive area on the PVR membrane in the vitreous cavity (n = 8, 1 eye from each of 8 mice).

Data in **a**, **b**, **e** are presented as mean ± s.d.. Data in **a** were compared using unpaired two-tailed Student's t-test. Data in **b** and **e** were compared using Mann-Whitney U test.

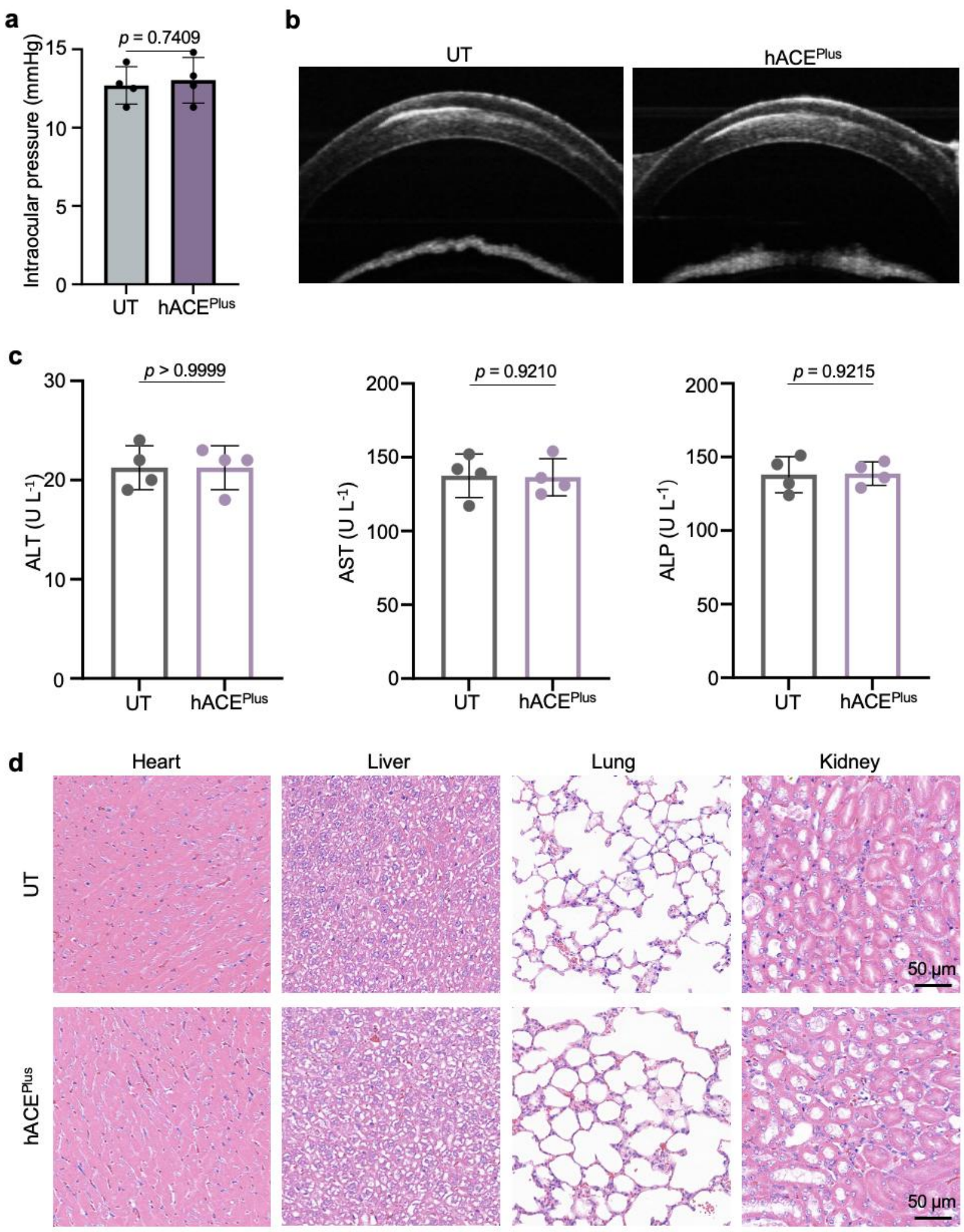

Supplementary Fig. 15 | Biosafety evaluation of humanized ACE^{Plus} (hACE^{Plus}) in NTG mice, related to Fig. 6.

a Intraocular pressure after UT or treatment with hACE^{Plus} in NTG mice (n = 4, 1 eye from each of 4 mice).

b Anterior segment morphology after UT or treatment with hACE^{Plus} in NTG mice.

c Serum biochemical indices, including ALT, AST, and ALP (n = 4 mice).

d Representative H&E staining images of the heart, liver, lung, and kidney tissues.

Data in **a** and **c** are presented as the mean ± s.d. and were compared using unpaired two-tailed Student's t-test.

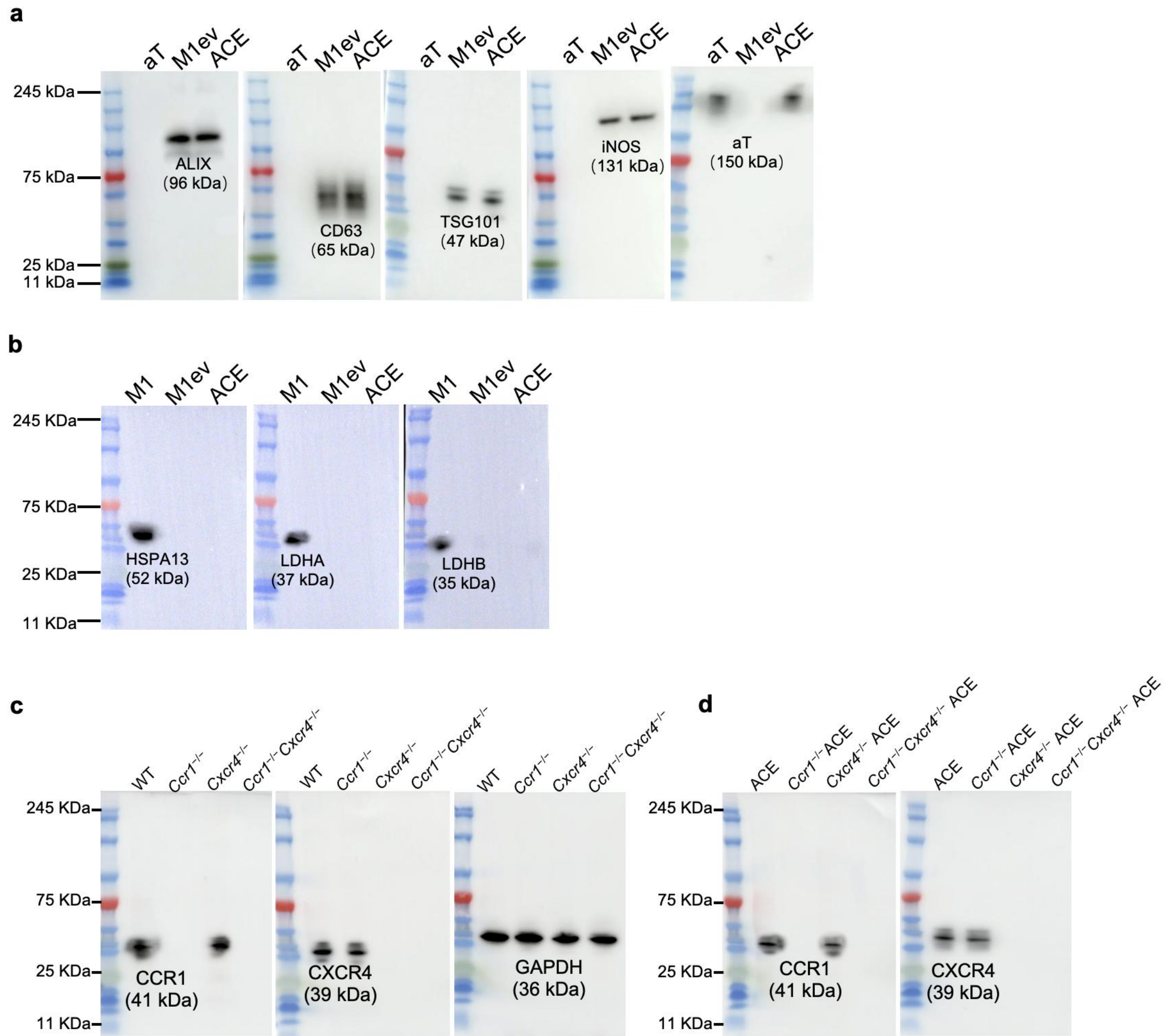

Supplementary Fig. 16 | Full-length western blot images related to Supplementary Figs. 3b,c and 4b,c.

a Original western blot images of Supplementary Fig. 3b.

b Original western blot images of Supplementary Fig. 3c.

c Original western blot images of Supplementary Fig. 4b.

d Original western blot images of Supplementary Fig. 4c.

Supplementary Table 1 | Clinical characteristics of a non-proliferative vitreoretinopathy (non-PVR) donor and PVR patients, related to Figs. 1 and 6.

ID	Gender	Non-PVR donor/ PVR patient	PVR grade	Application
1	Male	PVR	C3	RNA-seq
2	Male	PVR	C2	RNA-seq
3	Male	PVR	C3	RNA-seq
4	Female	PVR	C3	RNA-seq
5	Male	PVR	C2	RNA-seq
6	Male	PVR	C3	RNA-seq
7	Male	PVR	C3	IHC
8	Male	PVR	C2	IHC
9	Female	PVR	C3	IHC
10	Male	Non-PVR	NA	IHC
11	Male	PVR	C3	PDMX
12	Female	PVR	C3	PDMX
13	Male	PVR	C3	PDMX
14	Female	PVR	C3	PDMX
15	Female	PVR	C2	PDMX
16	Male	PVR	C3	PDMX
17	Male	PVR	C2	PDMX
18	Female	PVR	C3	PDMX
19	Male	PVR	C3	PDMX
20	Male	PVR	C2	PDMX
21	Male	PVR	C3	PDMX
22	Female	PVR	C3	PDMX

Supplementary Table 2 | Grading scale for the mouse model of PVR, related to Figs. 3, 5, and 6.

Grade	**Characteristics**	
	Vitreous	RD area
0	Clear	NA
1	Hazy	NA
2	Cells around vessels	NA
3	Cells forming tractional membrane	Very localized
4	Tractional membrane	<25%
5	Tractional membrane	25–75%
6	Tractional membrane	75–100%

Supplementary Table 3 | Oligonucleotide sequences used in this study.

Gene	Designation	Sequence (5' to 3')
Ccr1	sgRNA-1	CAACTACAGAATTTGACTATGGG
	sgRNA-2	TGATGAACACTAGAGAATACAGG
	Forward Primer	GTTCCCCTCTTAGTCTACAAGGTCC
	Reverse Primer	CACACTGATTGGTGAATAGAACATCTTGG
Cxcr4	sgRNA-1	GGAGCATGACGGACAAGTACCGG
	sgRNA-2	CCCAAAGTACCAGTCAGCCATGG
	Forward Primer	GAGGGCATATAACAAGTGACTGGTG
	Reverse Primer 1	TTGTGCACGATGCTCTCGAAG
	Reverse Primer 2	CCAGAAGGGGAGTGTGATGACAAAG

The PAM sequence is underlined.
