# Supplementary material for: Therapeutic modulation of the vitreoretinal fibrosis microenvironment in female mice using engineered macrophage-derived extracellular vesicles
Source: Nat Commun. 2026 Aug 25;17:10180. doi: 10.1038/s41467-026-76550-z (PMC13612821; doi:10.1038/s41467-026-76550-z)
Supplement: Supplementary file 2 [file 41467_2026_76550_MOESM2_ESM.pdf]

nature portfolio

Corresponding author(s): Guanghui Ma, Yong Tao, Ying Tian, Wei Wei

Last updated by author(s): Jul 24, 2026

Reporting Summary

Nature Portfolio wishes to improve the reproducibility of the work that we publish. This form provides structure for consistency and transparency in reporting. For further information on Nature Portfolio policies, see our Editorial Policies and the Editorial Policy Checklist.

Statistics

For all statistical analyses, confirm that the following items are present in the figure legend, table legend, main text, or Methods section.

n/a	Confirmed	
☐	☒	The exact sample size (*n*) for each experimental group/condition, given as a discrete number and unit of measurement
☐	☒	A statement on whether measurements were taken from distinct samples or whether the same sample was measured repeatedly
☐	☒	The statistical test(s) used AND whether they are one- or two-sided<br>*Only common tests should be described solely by name; describe more complex techniques in the Methods section.*
☒	☐	A description of all covariates tested
☐	☒	A description of any assumptions or corrections, such as tests of normality and adjustment for multiple comparisons
☐	☒	A full description of the statistical parameters including central tendency (e.g. means) or other basic estimates (e.g. regression coefficient) AND variation (e.g. standard deviation) or associated estimates of uncertainty (e.g. confidence intervals)
☐	☒	For null hypothesis testing, the test statistic (e.g. *F*, *t*, *r*) with confidence intervals, effect sizes, degrees of freedom and *P* value noted<br>*Give P values as exact values whenever suitable.*
☒	☐	For Bayesian analysis, information on the choice of priors and Markov chain Monte Carlo settings
☒	☐	For hierarchical and complex designs, identification of the appropriate level for tests and full reporting of outcomes
☐	☒	Estimates of effect sizes (e.g. Cohen's *d*, Pearson's *r*), indicating how they were calculated

Our web collection on statistics for biologists contains articles on many of the points above.

Software and code

Policy information about availability of computer code

Data collection	Images of ACE or ACEPlus were obtained using TEM (JEM-1400, JEOL). The size distribution of ACE or ACEPlus were determined using nanoparticle tracking analysis (PMX120, ZetaView). Nano-flow cytometry data were acquired via flow cytometry (CytoFLEX Nano, Beckman Coulter). Images of ACE distribution were collected using an in vivo imaging system (530 FX Pro, Kodak) with associated software. IHC staining images were scanned using a Pannoramic MIDI system (3DHISTECH, Hungary) and analyzed with Aipathwell (version 2) software. Fluorescent images were collected using CLSM (UltraVIEW VoX, PerkinElmer) with NIS-elements software (v.5.20.00 version). The visual cliff data were recorded using a video camera and analyzed with the ANY-maze Video Tracking System (version 7.48). OCT images were acquired using Ultramicro Ophthalmol Imaging System (Optoprobe Science Ltd., The United Kingdom). CFP images of the extracted porcine eye were acquired using a colour fundus camera. ERGdata were recorded using a visual electrophysiology instrument (OPTO-ERG, Optoprobe Science Ltd., UK). Intraocular pressure measurements were obtained using iCare Tonolab (TonoLab, Icare, Finland).
Data analysis	All statistical analyses were performed on GraphPad Prism (version 9.0.0) and Origin 2022. Nano flow cytometry data were analysed on CytExpert nano 1.0 (version 1.0.0.651). Flow-cytometry data were analysed on CytExpert software (v.2.4.0.28 version). RNA-sequencing analysis was performed using the DESeq2 R package (v1.42.0 for clinical samples and v1.20.0 for animal and cell samples).The online platform OmicShare tools (https://www.omicshare.com/tools) was specifically utilized to perform correlation coefficient analyses.The CIBERSORTx analytical tool (https://cibersortx.stanford.edu/) was employed to evaluate immune cell infiltration profiles. Fluorescence images and Western blot data were analyzed using ImageJ software.

For manuscripts utilizing custom algorithms or software that are central to the research but not yet described in published literature, software must be made available to editors and reviewers. We strongly encourage code deposition in a community repository (e.g. GitHub). See the Nature Portfolio guidelines for submitting code & software for further information.

Data

Policy information about availability of data

All manuscripts must include a data availability statement. This statement should provide the following information, where applicable:

- Accession codes, unique identifiers, or web links for publicly available datasets
- A description of any restrictions on data availability
- For clinical datasets or third party data, please ensure that the statement adheres to our policy

The main data supporting the results in this study are available within the paper and its Supplementary Information. The RNA-sequencing data are available from the NCBI BioProject via the accession code PRJNA1415138. The mass spectrometry and proteomics data are available from the ProteomeXchange Consortium (through the iProX partner repository) via the dataset identifier PXD073679.Source Data are provided with this paper and are also available in Figshare (https://doi.org/10.6084/m9.figshare.33080657).

Research involving human participants, their data, or biological material

Policy information about studies with human participants or human data. See also policy information about sex, gender (identity/presentation), and sexual orientation and race, ethnicity and racism.

Reporting on sex and gender	This study utilized both internally collected clinical samples and publicly available datasets. For the internal cohort, the sex of each participant was extracted from routine clinical records, which reflect patient self-reporting at hospital admission; it was not assigned or inferred by the researchers. Sex was not considered a primary confounding variable in the study design, as the fundamental pathological mechanisms of proliferative vitreoretinopathy (PVR) investigated here are generally not considered to be sex-specific. For public datasets, sex annotations were provided by the original investigators and similarly not analyzed for sex-specific differences.
Reporting on race, ethnicity, or other socially relevant groupings	For the internal cohort, Race and ethnicity were not utilized as primary selection criteria for this study. For the internal cohort, participants are of Asian descent, which naturally reflects the local demographic of the patient population served by the collecting center in Beijing, China. For public datasets, any demographic categorizations were defined by the original investigators.
Population characteristics	The study involved 22 human participants in the internal cohort, consisting of 21 patients with PVR and 1 non-PVR donor. All participants were of Asian descent, and their ages ranged from 22 to 81 years. To strictly adhere to patient privacy and de-identification guidelines, specific individual ages are omitted, with available individual baseline characteristics (including sex and clinical diagnosis) provided in Table S1. For the publicly available datasets, participant demographics and clinical statuses rely entirely on the metadata provided by the original investigators.
Recruitment	For the internal cohort, patients with PVR undergoing vitreoretinal surgery at Beijing Chaoyang Hospital were considered for inclusion. Enrollment relied on routine clinical diagnosis and general sample suitability. The control consisted of a non-PVR donor with no clinical evidence of fundus or retinal abnormalities. Regarding the public datasets, participant recruitment and cohort definitions were managed entirely by the original investigators, and we utilized these data acknowledging that the primary recruitment parameters were established externally.
Ethics oversight	The collection of clinical samples and subsequent experiments for the internal cohort were approved by the Ethics Committee of Beijing Chaoyang Hospital (approval number: 2022-S-590). For the publicly available datasets, initial data collection and ethical approvals were secured by the original investigators. As these public datasets are fully de-identified, their secondary use in this study is exempt from additional ethical review.

Note that full information on the approval of the study protocol must also be provided in the manuscript.

Field-specific reporting

Please select the one below that is the best fit for your research. If you are not sure, read the appropriate sections before making your selection.

[x] Life sciences [ ] Behavioural & social sciences [ ] Ecological, evolutionary & environmental sciences

For a reference copy of the document with all sections, see nature.com/documents/nr-reporting-summary-flat.pdf

Life sciences study design

All studies must disclose on these points even when the disclosure is negative.

Sample size	Sample sizes were provided in the figure legends for each experiment and reasonable sample sizes were chosen to ensure they are sufficient for statistical comparison between different groups. We also referred to relevant literature to determine sample sizes. For the in vitro and in vivo experiments, we followed standards of good scientific practice. We used at least 3 biological replicates or 3 animals per group, to calculate means and standard deviations and to perform statistical analyses. Additionally, a clinical sample from a healthy donor was analyzed, but only one was available due to limited specimen accessibility.
Data exclusions	No data were excluded.
Replication	For all experiments, we report pooled results from multiple experiments or the data shown correspond to one representative experiment of

at least 3 experiments.

Randomization	For the in vitro experiments, samples were randomly allocated into experimental groups. For the in vivo studies, animals were randomly grouped.
Blinding	Investigators were not blinded for materials synthesis since the scientists must keep careful track of conditions. For the in vivo end-point evaluations, the investigators were blinded to group allocation during data collection and analysis.

Reporting for specific materials, systems and methods

We require information from authors about some types of materials, experimental systems and methods used in many studies. Here, indicate whether each material, system or method listed is relevant to your study. If you are not sure if a list item applies to your research, read the appropriate section before selecting a response.

Materials & experimental systems

n/a	Involved in the study	
☐	☒	Antibodies
☐	☒	Eukaryotic cell lines
☒	☐	Palaeontology and archaeology
☐	☒	Animals and other organisms
☒	☐	Clinical data
☒	☐	Dual use research of concern
☒	☐	Plants

Methods

n/a	Involved in the study	
☒	☐	ChIP-seq
☐	☒	Flow cytometry
☒	☐	MRI-based neuroimaging

Antibodies

Antibodies used

Anti-TGFβ1 (Abcam; ab215715; 1:500 dilution)
Anti-α-SMA (Abcam; ab7817; Use a concentration of 0.034 μg/mL)
Anti-Collagen1 (Abcam;ab88147; 1:1000 dilution),
Anti-MMP2 (Abcam; ab86607; Use a concentration of 2 μg/mL)
Anti-CXCR4 (Abcam; ab124824)
Anti-ALIX (Abcam; ab275377; 1:1000 dilution)
Anti-TSG101 (Abcam; ab125011; 1:1000 dilution)
Anti-CD63 (Abcam; ab217345; 1:1000 dilution)
Anti-iNOS (Abcam; ab178945; 1:1000 dilution)
Anti-GAPDH (Abcam; ab181602; 1:10000 dilution)
Anti-Liver Arginase (Abcam; ab124917; 1:1000 dilution)
Anti-Mannose Receptor (Abcam; ab64693; 1 μg/mL)
Anti-CD163(Abcam; ab182422; 0.5 μg/mL)
Anti-Stat6 (Abcam; ab32520; 1:1000 dilution)
Anti-STAT6 (phospho Y641) (Abcam; ab263947; 1:1000 dilution)
Goat Anti-Rabbit IgG H&L (HRP) (Abcam; ab205718; 1:10000 dilution)
Goat Anti-Mouse IgG H&L (HRP) (Abcam; ab205719; 1:10000 dilution)
Anti-Smad2 (Cell Signaling Technology; 5339; 1:1000 dilution)
Anti-Smad3 (Cell Signaling Technology; 9523; 1:1000 dilution)
Anti-phospho-Smad2 (Cell Signaling Technology; 18338; 1:1000 dilution)
Anti-phospho-Smad3 (Cell Signaling Technology; 9520; 1:1000 dilution)
Anti-CCR1 (ThermoFisher Scientific; PA1-41062)
Anti-IL4Rα (Immunoway; YT2337; 1:1000 dilution)
Anti-IL13Rα1 (Immunoway; YT2313; 1:1000 dilution)
Anti-FN1 (Immunoway; YM8309; 1:2000 dilution)
Anti-Col1A1 (Immunoway; YM8576; 1:2000 dilution)
FITC anti-mouse CD80 antibody (BioLegend; 104705)
PE anti-mouse F4/80 antibody (BioLegend; 123109)
APC anti-mouse CD206 antibody (BioLegend; 141708)

Validation

We relied on publications and on validation sources cited by manufacturer. Links for each antibody are given below:

Anti-TGFβ1 (Abcam, ab215715)
https://www.abcam.cn/products/primary-antibodies/tgf-beta-1-antibody-epr21143-ab215715.html
Anti-α-SMA (Abcam;ab7817)
https://www.abcam.cn/products/primary-antibodies/alpha-smooth-muscle-actin-antibody-1a4-ab7817.html
Anti-Collagen1 (Abcam;ab88147)
https://www.abcam.cn/products/primary-antibodies/collagen-i-antibody-3g3-bsa-and-azide-free-ab88147.html

Anti-MMP2 (Abcam; ab86607)
https://www.abcam.cn/products/primary-antibodies/mmp2-antibody-6e3f8-ab86607.html
Anti-CXCR4 (Abcam, ab124824)
https://www.abcam.cn/products/primary-antibodies/cxcr4-antibody-umb2-ab124824.html
Anti-ALIX (Abcam, ab275377)
https://www.abcam.cn/products/primary-antibodies/tsg101-antibody-epr7130b-ab125011.html
Anti-TSG101 (Abcam; ab125011)
https://www.abcam.cn/products/primary-antibodies/tsg101-antibody-epr7130b-ab125011.html
Anti-CD63 (Abcam; ab217345)
https://www.abcam.cn/products/primary-antibodies/cd63-antibody-epr21151-ab217345.html
Anti-iNOS (Abcam; ab178945)
https://www.abcam.cn/products/primary-antibodies/inos-antibody-epr16635-ab178945.html
Anti-GAPDH (Abcam; ab181602)
https://www.abcam.cn/products/primary-antibodies/gapdh-antibody-epr16891-loading-control-ab181602.html
Anti-Liver Arginase (Abcam; ab124917)
https://www.abcam.cn/products/primary-antibodies/liver-arginase-antibody-epr6671b-ab124917.html
Anti-Mannose Receptor (Abcam; ab64693)
https://www.abcam.cn/products/primary-antibodies/mannose-receptor-antibody-ab64693.html
Anti-CD163(Abcam; ab182422)
https://www.abcam.cn/products/primary-antibodies/cd163-antibody-epr19518-ab182422
Anti-Stat6 (Abcam; ab32520)
https://www.abcam.cn/products/primary-antibodies/stat6-antibody-ye361-ab32520.html
Anti-STAT6 (phospho Y641) (Abcam; ab263947)
https://www.abcam.cn/products/primary-antibodies/stat6-phospho-y641-antibody-epr22599-78-ab263947.html
Goat Anti-Rabbit IgG H&L (HRP) (Abcam; ab205718)
https://www.abcam.cn/products/secondary-antibodies/goat-rabbit-igg-hl-hrp-ab205718.html
Goat Anti-Mouse IgG H&L (HRP) (Abcam; ab205719)
https://www.abcam.cn/products/secondary-antibodies/goat-mouse-igg-hl-hrp-ab205719.html
Anti-Smad2 (Cell Signaling Technology; 5339)
https://www.cellsignal.cn/products/primary-antibodies/smad2-d43b4-xp-rabbit-mab/5339
Anti-Smad3 (Cell Signaling Technology; 9523)
https://www.cellsignal.cn/products/primary-antibodies/smad3-c67h9-rabbit-mab/9523
Anti-phospho-Smad2 (Cell Signaling Technology; 18338)
https://www.cellsignal.cn/products/primary-antibodies/phospho-smad2-ser465-ser467-e8f3r-rabbit-mab/18338
Anti-phospho-Smad3 (Cell Signaling Technology; 9520)
https://www.cellsignal.cn/products/primary-antibodies/phospho-smad3-ser423-425-c25a9-rabbit-mab/9520
Anti-CCR1 (ThermoFisher Scientific; PA1-41062)
https://www.thermofisher.cn/cn/zh/antibody/product/CCR1-Antibody-Polyclonal/PA1-41062
Anti-IL4Rα (Immunoway; YT2337)
https://www.immunoway.com/products/primary-antibodies/YT2337-IL-4Ra-Rabbit-pAb.html
Anti-IL13Rα1 (Immunoway; YT2313)
https://www.immunoway.com/products/primary-antibodies/YT2313-IL-13Ra1-Rabbit-pAb.html
Anti-FN1 (Immunoway; YM8309)
https://www.immunoway.com/products/primary-antibodies/YM8309-Fibronectin-PT0476R-Rabbit-mAb.html
Anti-Col1A1 (Immunoway; YM8576)
https://www.immunoway.com/products/primary-antibodies/YM8576-Collagen-I-A1-PT0817R-Rabbit-mAb.html
FITC anti-mouse CD80 antibody (BioLegend; 104705)
https://www.biolegend.com/en-us/products/fitc-anti-mouse-cd80-antibody-41
PE anti-mouse F4/80 antibody (BioLegend; 123109)
https://www.biolegend.com/en-us/products/pe-anti-mouse-f4-80-antibody-4068
APC anti-mouse CD206 antibody (BioLegend; 141708)
https://www.biolegend.com/en-us/products/apc-anti-mouse-cd206-mmr-antibody-7425

Eukaryotic cell lines

Policy information about cell lines and Sex and Gender in Research

Cell line source(s)	The cell lines used in this manuscript, ARPE-19 (CL-0026), and THP-1 (CL-0233), were purchased from Pricella Life Science & Technology, while the RAW264.7 (YC-C020) was obtained from Ubigene .
Authentication	The cell line was authenticated by species profiling.
Mycoplasma contamination	The cell line was tested for mycoplasma contamination. No mycoplasma contamination was found.
Commonly misidentified lines (See ICLAC register)	No commonly misidentified cell lines were used.

Animals and other research organisms

Policy information about studies involving animals; ARRIVE guidelines recommended for reporting animal research, and Sex and Gender in Research

Laboratory animals	C57BL/6J mice (6–8 weeks, weight of 20 ± 1 g) were purchased from Charles River (Beijing, China). The mice were housed in an environmentally controlled room (23 °C, with 55 ± 5% humidity and under a 12-h–12-h light–dark cycle)<br><br>NTG mice aged 6–8 weeks (20 ± 1 g) were procured from SPF Biotechnology Co., Ltd. (Beijing, China).
Wild animals	The study did not involve wild animals.
Reporting on sex	Consistent with previous reports establishing the PVR model, we selected female mice to minimize confounding factors and ensure model reproducibility. Sex was determined based on supplier documentation and anatomical inspection.
Field-collected samples	The study did not involve samples collected from the field.
Ethics oversight	The mouse experiments were approved by the Institutional Animal Care and Use Committee at the Institute of Process Engineering, Chinese Academy of Sciences (Number: IPEAECA 2021112).

Note that full information on the approval of the study protocol must also be provided in the manuscript.

Plants

Seed stocks	*Report on the source of all seed stocks or other plant material used. If applicable, state the seed stock centre and catalogue number. If plant specimens were collected from the field, describe the collection location, date and sampling procedures.*
Novel plant genotypes	*Describe the methods by which all novel plant genotypes were produced. This includes those generated by transgenic approaches, gene editing, chemical/radiation-based mutagenesis and hybridization. For transgenic lines, describe the transformation method, the number of independent lines analyzed and the generation upon which experiments were performed. For gene-edited lines, describe the editor used, the endogenous sequence targeted for editing, the targeting guide RNA sequence (if applicable) and how the editor was applied.*
Authentication	*Describe any authentication procedures for each seed stock used or novel genotype generated. Describe any experiments used to assess the effect of a mutation and, where applicable, how potential secondary effects (e.g. second site T-DNA insertions, mosiacism, off-target gene editing) were examined.*

Flow Cytometry

Plots

Confirm that:

- [x] The axis labels state the marker and fluorochrome used (e.g. CD4-FITC).
- [x] The axis scales are clearly visible. Include numbers along axes only for bottom left plot of group (a 'group' is an analysis of identical markers).
- [x] All plots are contour plots with outliers or pseudocolor plots.
- [x] A numerical value for number of cells or percentage (with statistics) is provided.

Methodology

Sample preparation	Mouse peritoneal macrophages were collected, then filtered through a 40-μm cell strainer to isolate the cells for further analysis, as detailed in Methods.
Instrument	CytoFLEX LX flow cytometer (Beckman Coulter)
Software	CytExpert software (v.2.4.0.28 version)
Cell population abundance	The flow cytometry figures in the manuscript illustrate the abundance of relevant cell populations within post-sort fractions, with quantification methods detailed in the corresponding figure captions.
Gating strategy	Cells were initially gated on the starting cell population in a FSC/SSC plot to exclude debris, followed by a FSC-A/FSC-H plot to gate single cells. Live cells were then identified by gating on the Ghost V450-negative population. Subsequently, macrophages were defined as F4/80 (PE)-positive cells, and finally gated on CD80 (FITC) versus CD206 (APC) expression to evaluate the M1 polarization phenotype.

- [x] Tick this box to confirm that a figure exemplifying the gating strategy is provided in the Supplementary Information.
