# Supplementary material for: Therapeutic modulation of the vitreoretinal fibrosis microenvironment in female mice using engineered macrophage-derived extracellular vesicles
Source: Nat Commun. 2026 Aug 25;17:10180. doi: 10.1038/s41467-026-76550-z (PMC13612821; doi:10.1038/s41467-026-76550-z)
Supplement: Supplementary file 3 [file 41467_2026_76550_MOESM3_ESM.pdf]

nature portfolio

Peer Review File

Therapeutic modulation of the vitreoretinal fibrosis microenvironment using engineered macrophage-derived extracellular vesicles

Corresponding Author: Professor wei wei

This file contains all reviewer reports in order by version, followed by all author rebuttals in order by version.

Version 0:

Reviewer comments:

Reviewer #1

(Remarks to the Author)
This manuscript presents the ACE platform, an innovative therapeutic for proliferative vitreoretinopathy (PVR). The study is based on a rational design, moving from bioinformatic target identification to the rational engineering of M1ev. The development of a patient-derived xenograft (PDMX) model is a standout contribution, greatly enhancing the work's translational potential. The data support the conclusion that this platform can effectively modulate the fibrotic microenvironment. While compelling, the following suggestions would further strengthen the manuscript.
Major Comments:
1. The study's therapeutic strategy is contingent on the use of EVs from M1-polarized macrophages, but data confirming the polarization efficiency of the parent cells are currently absent. The authors should provide flow cytometry or qPCR data to verify a dominant M1 phenotype (e.g., high CD80/iNOS, low CD206/Arg1) in the parent cells prior to EV collection.
2. In Fig. S3, regarding the characterization of the ACE, the western blot analysis should include negative markers to align with MISEV2023 guidelines. Assessing for non-EV proteins (e.g., HSPA13, LDHA/B) is essential to confirm the purity of the EV preparation.
3. To further substantiate the advantage of the smart MMP-cleavable linker, its stability in a non-pathological environment should be demonstrated. Showing that the MMP2 concentration in normal vitreous humor is insufficient to cause premature antibody release would serve as a critical control.
4. Considering that M1ev may carry a pro-inflammatory payload, the possibility of an inflammatory response within the eye should be noted. It would be very helpful for readers if the authors could clarify whether any signs of adverse intraocular inflammation were noted following the injection of the ACE platform.
5. The discussion section should further emphasize the value of the innovative PDMX model as a versatile "platform". Highlighting its potential application for other vitreoretinal fibrotic diseases, such as proliferative diabetic retinopathy, would underscore the model's broad translational relevance.
Minor Comments:
1. For improved reproducibility, the methods section should be expanded. For instance, the authors should specify the antibody concentrations and incubation conditions used for the receptor blocking experiment referenced in Figure 2G.
2. The statistical reporting could be enhanced for greater rigor. The authors should report exact p-values (unless $p < 0.0001$) and include 95% confidence intervals for key data where appropriate.

Reviewer #2

(Remarks to the Author)
There are several noteworthy results from this excellent study on developing a treatment that is targeted to the M1 conversion to M2 which is the fibrotic macrophage found in vitreo-retinal fibrosis. The authors data mined a publicly available data set that compared PVR and non PVR clinical samples they then discovered from this differential display the cytokine transforming growth factor β1 (TGFβ1)as the highest ranking fibrotic cytokine in this panel furthermore they identified in the vitreoretinal fibrosis microenvironment the extensive presence of M2 macrophages.

The identification of TGFβ1 and M2 macrophages as overexpressed in the vitreoretinal fibrosis microenvironment derived from human specimens, confirms what has been hypothesized previously from in vitro experiments. Unfortunately many of

these prior studies were not comprehensive and methodologies were not stringent and were published in journals that I would not reference.
They then developed extracellular vesicles that were linked to antibodies to TGFβ1 and demonstrated their ability to accumulate in proliferative vitreoretinal (PVR) lesions. This in effect localized TGF beta antibodies and concentrated them in the vitreous retinal fibrosis microenvironment. They then demonstrated the ability to inhibit M1 to M2 polarization and TGFβ1 activity. As a follow on construct they incorporated in the extracellular vesicle PDGFRα antibody. PDGFRα had been documented by their differential display as the second most upregulated fibrotic cytokine in the vitreoretinal fibrotic microenvironment

They then tested these constructs not only in vitro but in vivo. They used a PVR mouse model to determine the efficacy of these constructs in vivo. They performed clinical trials with both safety and efficacy endpoints in these murine patients. Their conclusion is that the TGFβ1 linked extracellular vesicle was able to prevent the formation of PVR and using the construct of PDGFRα antibody linked to an extracellular vesicle they were able to treat an advanced stage PVR model
This paper is a significant breakthrough in the field of vitreoretinal fibrosis. While they targeted vitreoretinal fibrosis that is induced by retinal detachment or retinal trauma and surgery which constitutes a minority of the cases of blindness around the world, the prevalence of fibrosis as the end stage form of blinding conditions such as macular degeneration and proliferative diabetic retinopathy present the possibility of using this same strategy for these more prevalent blinding conditions worldwide.

While the use of antibodies targeting cytokines or growth factors such as PDGFRα antibody or TGFβ1 are not novel the linkage to the extra cellular vesicles that are derived from M1 macrophages is very novel. Furthermore, the efficacy demonstrated in well characterized in vivo models is extremely convincing considering very few therapeutic molecules that have been applied to these models have resulted in such profound suppression of the fibrotic process. The magnitude of effect is rather impressive if you compare it to the literature of other molecules that have attempted to be utilized to treat PVR. The use of molecular biology and omics tools to not only study the vitreoretinal fibrotic microenvironment but to then use those confirmatory findings to develop a therapeutic that will bypass the fibrotic capsule produced by late stage PVR to target and neutralize the cytokine that is responsible for the conversion of M1 macrophages into the fibrotic M2 state is rather ingenious.
Researchers on PVR have literally given up on this condition. Having begun my research on this condition over 45 years ago I am happy to see such advancements and now the potential for a viable efficacious treatment for these patients who go blind from trauma, retinal detachments or multiple ophthalmic surgeries.

In regards to the technical aspects of this article I see no flaw in the methodology, in the experimental execution or the description and writing of this article. I feel that this novel strategy for dealing with fibrosis is revolutionary. In the field of ophthalmology and retina, fibrosis represents our biggest challenge despite the advent of anti VEGF therapies. I would hope that they would utilize this strategy for other vitreoretinal indications that involve the innate immune activation and macrophage axis.

Reviewer #3

(Remarks to the Author)
The authors of the manuscript “Therapeutic modulation of the vitreoretinal fibrosis microenvironment using engineered macrophage-derived extracellular vesicles” designed and engineered extracellular vesicles with anti-fibrotic efficacy against proliferative vitreoretinopathy (PVR). These extracellular vesicles were derived from M1 macrophages and were conjugated with a TGFβ1 antibody (and platelet derived growth factor receptor antibody) through MMP-cleavable linkers to treat PVR at an early or advanced stages. The authors used a PVR mouse model and a patient-derived PVR membrane xenograft model to test the therapeutic efficacy of their vesicles in term of in preventing retinal detachment progression. The submitted work presents noteworthy and compelling results that make a clear contribution to the current research field. The results are coherent, internally consistent, and presented within a logically structured framework. It advances existing knowledge through innovative approaches and empirical analysis. The conclusions are supported by the presented data and analyses. The data analysis, interpretation, and conclusions show no major flaws, however, some aspects are listed below.
In summary, the publication makes a valuable contribution to its field and is therefore suitable for publication after revision.

Reviewer questions:
1. Figure 1E: Which markers were used to define the different macrophage populations?
2. CD206 is not an exclusive M2 marker, as it is also partially expressed by M1 populations. Wouldn’t it be reasonable to include additional macrophage markers, such as CD163 and CD68, in combination to distinguish between the different macrophage populations?
3. Figure 1P+Q: The visualization is difficult to interpret, as the circle sizes differ only minimally. Introducing a color distinction would make the figure easier to understand.
4. Results Figure 2G: In my opinion, a control using a receptor that is not expressed on ACE would be necessary to clearly demonstrate that the observed effects are mediated by CCR1 and CXCR4.
5. Figure 3A+B and Figure 5D: It would be helpful to have representative fundus and OCT images from day 0 to assess the extent of PVR at the start of therapy.
6. In some sections of the Materials and Methods, critical details are missing. For example, the number of cells in the single-cell suspension used for xenotransplantation, the exact storage conditions for the vesicles, and the parameters for ultracentrifugation are not specified. This section should be revised to ensure accuracy and reproducibility.

Reviewer #4

(Remarks to the Author)
This manuscript explores engineered M1-macrophage-derived EVs (ACE/ACEPlus) delivering anti-TGFβ1 and anti-PDGFRα antibodies via MMP-cleavable linkers to treat proliferative vitreoretinopathy (PVR). The work is innovative and clinically meaningful, especially with the introduction of a patient-derived xenograft PVR model that increases translational value.
However, several key mechanistic and methodological issues need to be resolved before the therapeutic claims can be supported.

Major comments

1) Mechanistic evidence remains associational

The impact of ACE is described primarily by static marker reduction (α-SMA, p-Smad2/3, Arg1, STAT6) [Fig. 4E – F], without direct demonstration that:
• Effects are M2-macrophage-dependent
• Spatiotemporal MMP2-mediated linker cleavage drives efficacy
• TGF-β suppression causally prevents myofibroblast transition

Proposed experiments:
• Macrophage depletion or polarization-rescue studies
• MMP2 inhibition in vivo confirming linker necessity
• Functional macrophage assays (migration/phagocytosis/cytokine secretion)

2) Inflammatory safety of M1-EVs is not addressed

M1 EVs may induce innate inflammatory activation. Only gross histology and IOP are reported.

Proposed additions:
• Microglia activation markers (IBA1, C3, TMEM119)
• Inflammatory cytokines (TNF-α, IL-1β, IL-6)
• Comparison vs naïve EVs (M0-EVs)

3) Biodistribution and bioavailability

The authors state that aT accumulates at the lesion site (Fig. 2H), but no quantitative biodistribution is provided.
• Such as retina vs vitreous vs RPE/choroid compartments and whether EVs cross ILM into preretinal fibrosis.

Minor comments

• No description of blinding and randomization: To add per ARRIVE guidelines
• To clarify n numbers for each assay: Add into figure legends

This work represents an important step toward pharmacologic intervention for PVR. With the above revisions — particularly mechanistic validation, inflammatory safety assessment, and biodistribution — the study could merit publication in Nature Communications.

Version 1:

Reviewer comments:

Reviewer #1

(Remarks to the Author)
No further comments.

Reviewer #3

(Remarks to the Author)
The authors answered all questions in great detail and conducted many additional experiments to answer them. Overall, all questions were answered satisfactorily and I have no further questions. I recommend accepting the publication.

Reviewer #4

(Remarks to the Author)
I am acceptable of the revision done by the authors.

Open Access This Peer Review File is licensed under a Creative Commons Attribution 4.0 International License, which permits use, sharing, adaptation, distribution and reproduction in any medium or format, as long as you give appropriate credit to the original author(s) and the source, provide a link to the Creative Commons license, and indicate if changes were made.
In cases where reviewers are anonymous, credit should be given to 'Anonymous Referee' and the source.
The images or other third party material in this Peer Review File are included in the article's Creative Commons license, unless indicated otherwise in a credit line to the material. If material is not included in the article's Creative Commons license and your intended use is not permitted by statutory regulation or exceeds the permitted use, you will need to obtain permission directly from the copyright holder.
To view a copy of this license, visit https://creativecommons.org/licenses/by/4.0/

RESPONSES TO REVIEWERS

(Note: All the responses here are in **blue**. All the changes are in **red** in the main text of revised manuscript.)

Reviewer #1 (Remarks to the Author):

This manuscript presents the ACE platform, an innovative therapeutic for proliferative vitreoretinopathy (PVR). The study is based on a rational design, moving from bioinformatic target identification to the rational engineering of M1ev. The development of a patient-derived xenograft (PDMX) model is a standout contribution, greatly enhancing the work's translational potential. The data support the conclusion that this platform can effectively modulate the fibrotic microenvironment. While compelling, the following suggestions would further strengthen the manuscript.

Response: We appreciate the reviewer's positive evaluation of the novelty, rational design, and translational relevance of the ACE platform, as well as the recognition of the patient-derived xenograft (PDMX) model as a key contribution. Following the reviewer's valuable guidance, we have conducted additional experiments, supplemented the discussion on the potential of PDMX, provided additional methodological details, and refined the data presentation, which together have substantially improved the manuscript.

Major Comments:

1. The study's therapeutic strategy is contingent on the use of EVs from M1-polarized macrophages, but data confirming the polarization efficiency of the parent cells are currently absent. The authors should provide flow cytometry or qPCR data to verify a dominant M1 phenotype (e.g., high CD80/iNOS, low CD206/Arg1) in the parent cells prior to EV collection.

Response: We thank the reviewer for this insightful comment. In response, we have now included the flow cytometry data characterizing the parent macrophages used for extracellular vesicle isolation. Using a standard sequential gating strategy, we excluded debris, doublets, and dead cells to isolate the F4/80-positive population. The analysis demonstrated that these cells exhibited a dominant M1 phenotype, distinguished by high CD80 and low CD206 expression at the time of collection (Fig. R1).

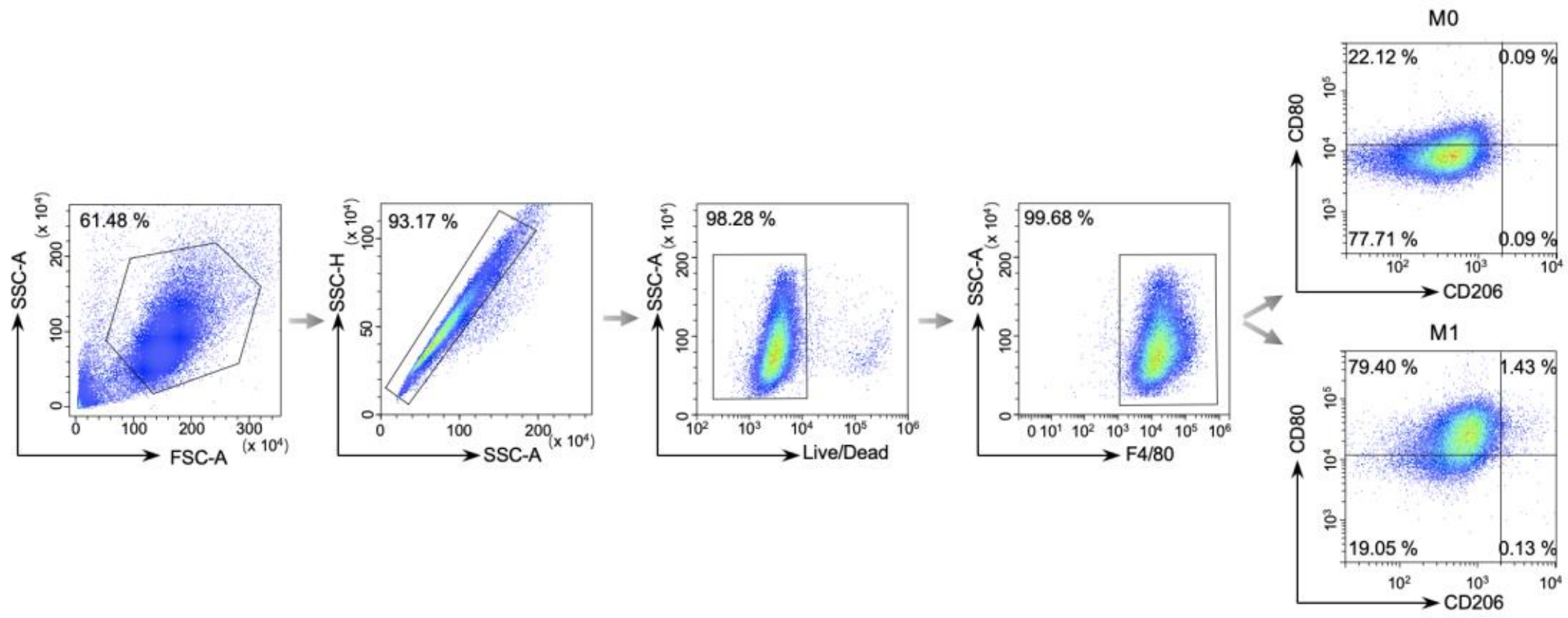

Fig. R1 (fig. S3a in revision). Flow cytometry gating strategy and phenotypic characterization of M1 macrophages.

Above results have now been added as fig. S3a in the revised manuscript with corresponding figure caption. We have also revised the corresponding statement in the main text.

Main text:

Page 4, **line 143-144, 146**: Characterization of ACE with transmission electron microscopy (TEM) indicated a cup-like morphology (Fig. 1k) and an average diameter of approximately 130 nm. The successful conjugation of aT on the surface of M1ev, derived from macrophages with an M1-dominant phenotype (fig. S3a), was verified by western blotting analysis, showing the coexistence of extracellular vesicle markers (CD63, ALIX, and TSG101), M1 macrophage specific protein (iNOS), and conjugated aT (fig. S3b,c).

Methods:

Page 12, **line 497–499**: FITC anti-mouse CD80 (104705), PE anti-mouse F4/80 (123109), and APC anti-mouse CD206 (141708) antibodies were purchased from BioLegend.

Page 15, **line 602–606**:

Mouse M1 macrophages were generated by isolating primary macrophages from the peritoneal cavity of C57BL/6 mice, followed by stimulation with lipopolysaccharide (LPS, 1 μg mL^{-1}) for 48 hours. After treatment, cells were thoroughly washed to remove residual LPS. The polarization status of the cells was assessed by flow cytometry, with cells first stained with Ghost V450 viability dye at 4 °C for 30 min to exclude dead cells, followed by surface staining with fluorophore-conjugated antibodies against F4/80

(PE), CD80 (FITC), and CD206 (APC), and finally analyzed to evaluate macrophage identity and predominant M1 polarization.

2. In Fig. S3, regarding the characterization of the ACE, the western blot analysis should include negative markers to align with MISEV2023 guidelines. Assessing for non-EV proteins (e.g., HSPA13, LDHA/B) is essential to confirm the purity of the EV preparation.

Response: We thank the reviewer for reminding us of this issue. In accordance with the MISEV2023 guidelines[1], we have now included additional western blotting analysis for non-EV protein markers (LDHA/B and HSPA13) in Fig. R2 to assess potential non-vesicular contamination and to better document the purity of the ACE preparation.

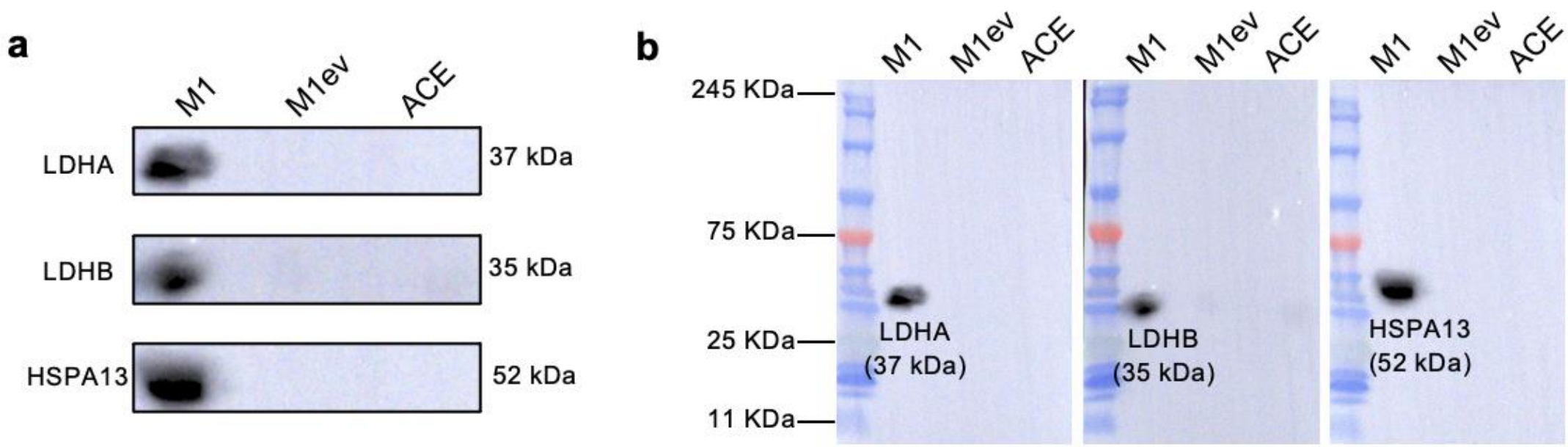

Fig. R2 (fig. S3d,e in revision). Additional characterization of ACE, related to Fig. 1.
a Western blotting analysis of the expression of LDHA, LDHB, and HSPA13 in parent M1 macrophages, M1ev, and ACE.
b Original western blotting images of Fig. R2a.

Above results have now been added as fig. S3d,e in the revised manuscript with corresponding figure captions. We have also revised the corresponding statement in the main text and methods.

Main text:

Page 4, line 146 – 148: Characterization of ACE with transmission electron microscopy (TEM) indicated a cup-like morphology (Fig. 1k) and an average diameter of approximately 130 nm. The successful conjugation of aT on the surface of M1ev, derived from macrophages with an M1-dominant phenotype (fig. S3a), was verified by western blotting analysis, showing the coexistence of extracellular vesicle markers (CD63, ALIX, and TSG101), M1 macrophage specific protein (iNOS), and conjugated aT (fig. S3b,c). Non-extracellular vesicle markers (LDHA, LDHB, and HSPA13) were absent, confirming the purity of the isolated extracellular vesicles[28] (fig. S3d,e).

Accordingly, comparing ACE to M1ev, we observed that nanoparticle tracking analysis (NTA) detected a slight decrease of zeta potential (Fig. 1l) and nano-flow cytometry revealed good colocalization efficiency of aT and M1ev (Fig. 1m).

Reference:

28. Welsh, J. A., Goberdhan, D. C. I., O'Driscoll, L., Buzas, E. I., Blenkiron, C., Bussolati, B., Cai, H., Di Vizio, D., Driedonks, T. A. P., Erdbrügger, U., Falcon-Perez, J. M., Fu, Q. L., Hill, A. F., Lenassi, M., Lim, S. K., Mahoney, M. G., Mohanty, S., Möller, A., Nieuwland, R., Ochiya, T., Witwer, K. W. (2024). Minimal information for studies of extracellular vesicles (MISEV2023): From basic to advanced approaches. Journal of extracellular vesicles, 13(2), e12404.

Methods:

Page 12, line 486–488: Anti-TGFβ1 (ab215715), anti-Mannose Receptor (ab64693), anti-α-SMA (ab7817), anti-Collagen1 (ab88147), anti-MMP2 (ab86607), anti-CXCR4 (ab124824), anti-ALIX (ab275377), anti-TSG101 (ab125011), anti-CD63 (ab217345), anti-iNOS (ab178945), anti-GAPDH (ab181602), anti-Liver Arginase (ab124917), anti-Stat6 (ab32520), anti-phospho-Stat6 (Y641) (ab263947), Goat Anti-Rabbit IgG H&L (HRP) (ab205718), and Goat Anti-Mouse IgG H&L (HRP) (ab205719) antibodies were purchased from Abcam. Anti-HSPA13 (DF1307), anti-LDHA (DF6280), and anti-LDHB (DF12101) antibodies were purchased from Affinity. Anti-Smad2 (5339), anti-Smad3 (9523), anti-phospho-Smad2 (18338), and anti-phospho-Smad3 (9520) antibodies were purchased from Cell Signaling Technology.

Page 16, line 641–642: The levels of ALIX, CD63, TSG101, iNOS, aT, β-actin, and non-extracellular vesicle markers (HSPA13, LDHA, and LDHB) in aT, M1 macrophages, M1ev, and ACE samples were evaluated by Western blot.

Reference in response:

1. Welsh, J. A., Goberdhan, D. C. I., O'Driscoll, L., Buzas, E. I., Blenkiron, C., Bussolati, B., Cai, H., Di Vizio, D., Driedonks, T. A. P., Erdbrügger, U., Falcon-Perez, J. M., Fu, Q. L., Hill, A. F., Lenassi, M., Lim, S. K., Mahoney, M. G., Mohanty, S., Möller, A., Nieuwland, R., Ochiya, T., Witwer, K. W. (2024). Minimal information for studies of extracellular vesicles (MISEV2023): From basic to advanced approaches. Journal of extracellular vesicles, 13(2), e12404.

3. To further substantiate the advantage of the smart MMP-cleavable linker, its stability

in a non-pathological environment should be demonstrated. Showing that the MMP2 concentration in normal vitreous humor is insufficient to cause premature antibody release would serve as a critical control.

Response: We thank the reviewer for this constructive suggestion. In response, we quantified MMP2 concentrations in normal vitreous humor and PVR lesions by ELISA and evaluated antibody release profiles under these conditions. As shown in Fig. R3, aT release in normal vitreous humor was significantly lower than that observed under pathological MMP2 conditions and did not differ significantly from that observed in the presence of an MMP2 inhibitor, indicating that physiological MMP2 levels fall below the threshold required to trigger premature antibody release.

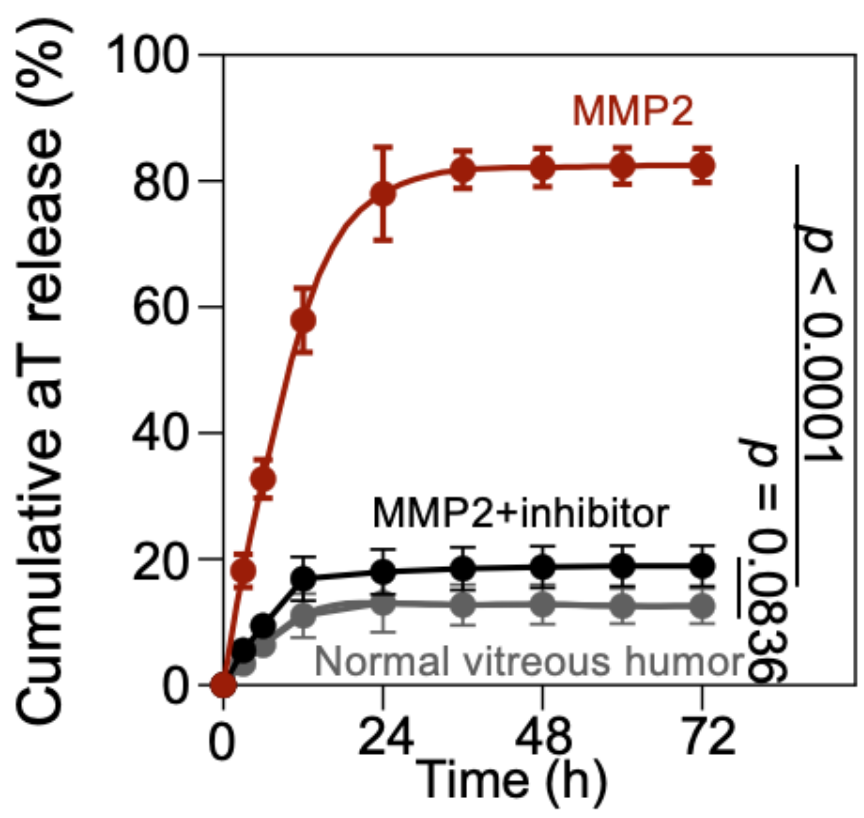

Fig. R3 (replaces Fig. 1n in revision). Additional characterization of ACE, related to Fig. 1.

Enzyme-linked immunosorbent assay (ELISA)-based quantification of aT levels following incubation in vitreous humor containing MMP2 at PVR (406.87 ng mL^{-1}, a concentration consistent with that at the mouse lesion site) or non-model control (3.38 ng mL^{-1}, normal vitreous humor) concentrations, with or without an inhibitor (n = 3 biologically independent experiments).

The data above are presented as the mean ± s.d. and were compared using one-way ANOVA.

Fig. R3 has now been replaced as Fig. 1n in the revised manuscript with corresponding figure captions. We have also revised the corresponding statement in the main text and methods.

Main text:

Page 4, line 157–158: As expected, the proportion of aT increased up to 78.0% within

24 h, indicating that most of the cl linker had been cleaved. In contrast, the cl linker was stable and released negligible aT at MMP2 levels found in normal vitreous humor. Once MMP2 inhibitor was incubated, the proportion of aT dramatically decreased (Fig. 1n), supporting the specificity of cleavage.

Page 26, line 1020–1022: n Enzyme-linked immunosorbent assay (ELISA)-based quantification of aT levels following incubation in vitreous humor containing MMP2 at PVR (406.87 ng mL^{-1}, a concentration consistent with that at the mouse lesion site) or NC (3.38 ng mL^{-1}, normal vitreous humor) levels, with or without an inhibitor (n = 3 biologically independent experiments).

4. Considering that M1ev may carry a pro-inflammatory payload, the possibility of an inflammatory response within the eye should be noted. It would be very helpful for readers if the authors could clarify whether any signs of adverse intraocular inflammation were noted following the injection of the ACE platform.

Response: We thank the reviewer for the opportunity to clarify that no detectable intraocular inflammation was observed following ACE platform injection. In the revised manuscript, we have included an assessment of intraocular inflammation. Specifically, ELISA analysis of TNF-α, IL-1β, and IL-6 in the aqueous humor showed no significant increase, consistent with the absence of clinical or histological signs of inflammation (Fig. R4).

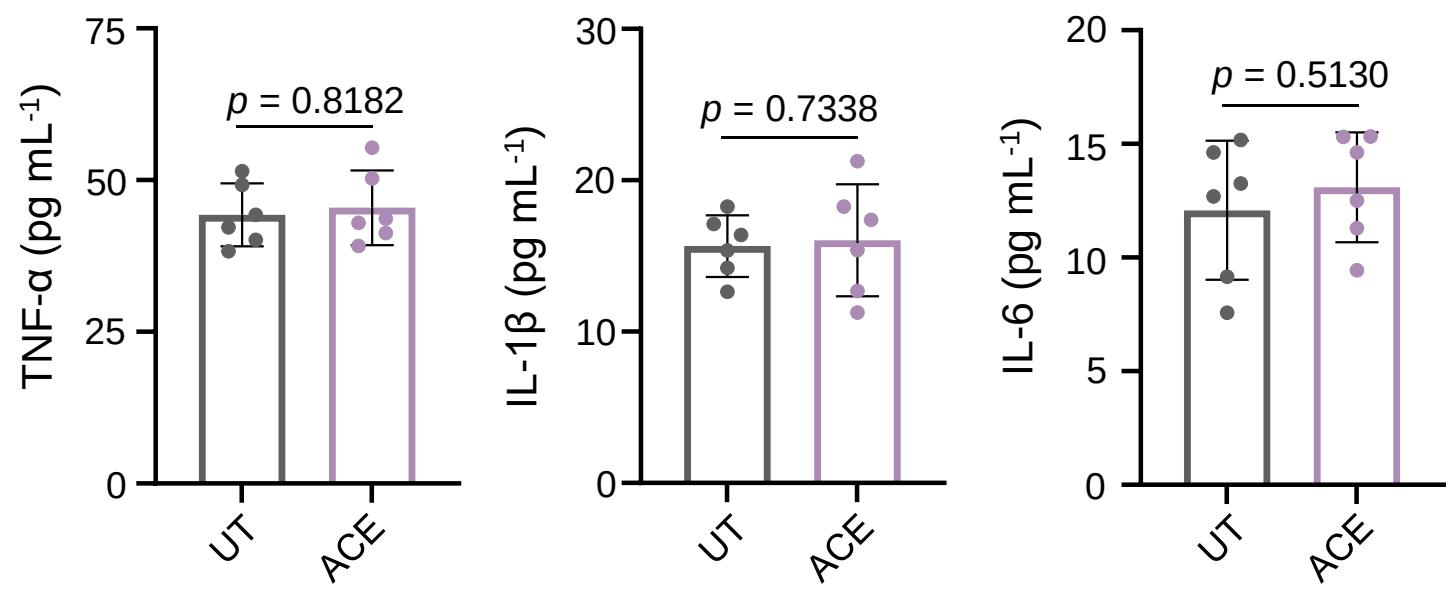

Fig. R4 (fig. S7c in revision). Biosafety evaluation of ACE, related to Fig. 3. Quantification of inflammatory cytokines (TNF-α, IL-1β, and IL-6) concentrations in the aqueous humor of untreated (UT) or ACE-treated C57BL/6 mice by ELISA (n = 6 biologically independent experiments).

The data above are represented as the mean ± s.d. and were compared using two-tailed unpaired Student's t-test.

Above results have now been added as fig. S7c in the revised manuscript with corresponding figure captions. We have also revised the corresponding statement in the main text and methods.

Main text:

Page 7, line 249–252: In addition to above therapeutic efficacy, the ACE treatment did not alter intraocular pressure (fig. S7a) or anterior segment morphology (fig. S7b), and no detectable signs of intraocular inflammation were observed following intravitreal injection (fig. S7c), collectively supporting the apparent safety of ACE upon local application.

Methods:

Page 12, line 495: ELISA kits (TGFβ1, MMP2, TNF-α, IL-1β, and IL-6) and DAPI were from Solarbio.

Page 19, line 785–787:

To assess the ocular safety and tolerance of the intravitreal injection of nanomedicines, intraocular pressure was measured using an iCare tonometer (TONOLAB, Finland), and the anterior segment was evaluated with an ultramicro ophthalmol imaging system (Optoprobe Science, United Kingdom). In addition, aqueous humor samples were collected for ELISA analysis of inflammatory cytokines to monitor potential intraocular inflammatory responses.

5. The discussion section should further emphasize the value of the innovative PDMX model as a versatile "platform". Highlighting its potential application for other vitreoretinal fibrotic diseases, such as proliferative diabetic retinopathy, would underscore the model's broad translational relevance.

Response: We thank the reviewer for this valuable suggestion, which strengthens the manuscript's focus on translational potential. In response, we have revised the Discussion section to emphasize the PDMX model’s applicability to other vitreoretinal fibrotic diseases, such as proliferative diabetic retinopathy and idiopathic epiretinal membrane, highlighting broad translational relevance. Accordingly, we have added the discussion in our revised manuscript as follows.

Main text:

Page 12, **line 470–474**: To address the challenges of conventional murine models in mimicking the complex pathophysiology of human PVR, we established the PDMX

murine model by engrafting surgically excised patients' PVR membranes into immunodeficient mice. This newly established and clinically relevant model faithfully recapitulated PVR pathology and vitreoretinal fibrosis microenvironment, providing a useful tool for evaluating the therapeutic efficacy, especially for human-derived antibodies that are species-specific and incompatible with conventional murine models. Additionally, the PDMX model offers an opportunity for in vivo drug sensitivity testing and personalized treatment strategy development for PVR. Surgically excised PVR membranes, routinely discarded, can be repurposed to create individualized PDMX models. These patient-specific models enable direct assessment of therapeutic responses to a panel of existing or investigational agents, facilitating the identification of the most effective treatment strategy for each individual. This PDMX model serves as a versatile platform for precision medicine, applicable not only to PVR but potentially to other vitreoretinal fibrotic conditions that share fibrotic mechanisms, such as proliferative diabetic retinopathy and idiopathic epiretinal membrane, which are driven by aberrant cell migration, phenotypic transition, and excessive ECM deposition.

Minor Comments:

1. For improved reproducibility, the methods section should be expanded. For instance, the authors should specify the antibody concentrations and incubation conditions used for the receptor blocking experiment referenced in Figure 2G.

Response: We appreciate the reviewer's careful review. The antibody concentration and incubation conditions used for the receptor-blocking experiment in Fig. 2g were 1:200 and 4°C overnight, respectively. We have now added the details in the methods, including other required expanded information.

Methods:

Page 18, line 709–710: A+E, AFE, ACE or ACE pretreated with different blocking strategies (PKH67-labeled M1ev) was intravitreally injected and allowed to release over a 24-hour period. Specifically, ACE was incubated with the corresponding chemokine receptor antibodies (CCR1, CXCR4) at a 1:100 dilution overnight at 4°C. Prior to enucleation, dextran was administered via tail vein injection to label retinal vasculature. Eyeballs were then collected, and retinas were flat-mounted for fluorescence imaging to quantify the selective accumulation of PKH67-labeled M1ev in lesion areas.

Page 14, line 577: These membranes were enzymatically digested into single-cell suspensions, and 1×10^4 cells were used for xenotransplantation.

Page 15, line 618–619:

The final extracellular vesicle preparations were resuspended in PBS and stored at 4 °C for short-term use (within 1 week) or at −80 °C for long-term storage prior to downstream applications. All samples were processed consistently following standardized procedures.

Page 15, line 615:

The clarified fluid was subsequently subjected to ultracentrifugation at 100,000 g for 70 minutes to pellet the extracellular vesicles.

2. The statistical reporting could be enhanced for greater rigor. The authors should report exact p-values (unless p < 0.0001) and include 95% confidence intervals for key data where appropriate.

Response: We thank the reviewer for this valuable suggestion to improve the statistical rigor of our study. In the revised manuscript, we have reported exact *p*-values (unless $p < 0.0001$) for all relevant statistical comparisons, with values for comparisons involving fewer groups included directly in the figure panels. Furthermore, we have included 95% confidence intervals for key quantitative data in the main text, specifically for the critical findings presented in Fig. 3g and Fig. 5i.

These updates have been incorporated into the revised figure panels and manuscript to ensure clarity and transparency, as detailed below.

Main text:

Page 25: Fig. 1e,l,n,o.

Page 28: Fig. 2c–g.

Page 30: Fig. 3d–g and j. **line 1099–1101:** Data in **d**–**g** and **j** are presented as mean ± s.d.. Data in **d**–**g** and **j** were compared using one-way ANOVA. In **g**, ACE resulted in a mean difference of 4.17 (95% confidence interval: 3.02 to 5.31) compared to PBS. ~~$*p < 0.05$, $**p < 0.01$, $***p < 0.001$, $****p < 0.0001$.~~

Page 32: Fig. 4h–j.

Page 34: Fig. 5f–l and n. **line 1173–1174:** Data in **f**–**l** and **n** are presented as means ± s.d.. Data in **f**–**l** and **n** were compared using one-way ANOVA. In **i**, the mean difference between the PBS and ACE^{Plus} groups was 3.00 (95% confidence interval: 2.03 to 3.97). ~~$**p < 0.01$, $***p < 0.001$, $****p < 0.0001$.~~

Page 36: Fig. 6m,o.

Methods:

Page 34, line 794–796: A two-sided p-value < 0.05 was considered statistically significant. Exact p-values are reported (unless $p < 0.0001$), and 95% confidence intervals for the difference in means are provided for key outcome measures. Significance levels were denoted as *$p < 0.05$, **$p < 0.01$, ***$p < 0.001$, and ****$p < 0.0001$ (ns, not significant). The correlations were analyzed by Spearman's rank correlation or Pearson correlation analysis.

Reviewer #2 (Remarks to the Author):

There are several noteworthy results from this excellent study on developing a treatment that is targeted to the M1 conversion to M2 which is the fibrotic macrophage found in vitreo-retinal fibrosis. The authors data mined a publicly available data set that compared PVR and non PVR clinical samples they then discovered from this differential display the cytokine transforming growth factor β1 (TGFβ1) as the highest ranking fibrotic cytokine in this panel furthermore they identified in the vitreoretinal fibrosis microenvironment the extensive presence of M2 macrophages.

The identification of TGFβ1 and M2 macrophages as overexpressed in the vitreoretinal fibrosis microenvironment derived from human specimens, confirms what has been hypothesized previously from in vitro experiments. Unfortunately many of these prior studies were not comprehensive and methodologies were not stringent and were published in journals that I would not reference.

They then developed extracellular vesicles that were linked to antibodies to TGFβ1 and demonstrated their ability to accumulate in proliferative vitreoretinal (PVR) lesions. This in effect localized TGF beta antibodies and concentrated them in the vitreous retinal fibrosis microenvironment. They then demonstrated the ability to inhibit M1 to M2 polarization and TGFβ1 activity. As a follow on construct they incorporated in the extracellular vesicle PDGFRα antibody. PDGFRα had been documented by their differential display as the second most upregulated fibrotic cytokine in the vitreoretinal fibrotic microenvironment.

They then tested these constructs not only in vitro but in vivo. They used a PVR mouse model to determine the efficacy of these constructs in vivo. They performed clinical trials with both safety and efficacy endpoints in these murine patients.

Their conclusion is that the TGFβ1 linked extracellular vesicle was able to prevent the formation of PVR and using the construct of PDGFRα antibody linked to an extracellular vesicle they were able to treat an advanced stage PVR model.

This paper is a significant breakthrough in the field of vitreoretinal fibrosis. While they targeted vitreoretinal fibrosis that is induced by retinal detachment or retinal trauma and surgery which constitutes a minority of the cases of blindness around the world, the prevalence of fibrosis as the end stage form of blinding conditions such as macular degeneration and proliferative diabetic retinopathy present the possibility of using this same strategy for these more prevalent blinding conditions worldwide.

While the use of antibodies targeting cytokines or growth factors such as PDGFRα antibody or TGFβ1 are not novel the linkage to the extra cellular vesicles that are

derived from M1 macrophages is very novel. Furthermore, the efficacy demonstrated in well characterized in vivo models is extremely convincing considering very few therapeutic molecules that have been applied to these models have resulted in such profound suppression of the fibrotic process. The magnitude of effect is rather impressive if you compare it to the literature of other molecules that have attempted to be utilized to treat PVR.

The use of molecular biology and omics tools to not only study the vitreoretinal fibrotic microenvironment but to then use those confirmatory findings to develop a therapeutic that will bypass the fibrotic capsule produced by late stage PVR to target and neutralize the cytokine that is responsible for the conversion of M1 macrophages into the fibrotic M2 state is rather ingenious.

Researchers on PVR have literally given up on this condition. Having begun my research on this condition over 45 years ago I am happy to see such advancements and now the potential for a viable efficacious treatment for these patients who go blind from trauma, retinal detachments or multiple ophthalmic surgeries.

In regards to the technical aspects of this article I see no flaw in the methodology, in the experimental execution or the description and writing of this article. I feel that this novel strategy for dealing with fibrosis is revolutionary. In the field of ophthalmology and retina, fibrosis represents our biggest challenge despite the advent of anti VEGF therapies. I would hope that they would utilize this strategy for other vitreoretinal indications that involve the innate immune activation and macrophage axis.

Response: We sincerely thank the reviewer for the insightful and encouraging comments on our work, particularly regarding the engineered M1ev design and its efficacy in suppressing vitreoretinal fibrosis.

We agree that the magnitude of effect observed in our study is notable when viewed in the context of prior efforts to treat proliferative vitreoretinopathy (PVR). In the Discussion, we have therefore included a direct comparison between our combinatorial ACE strategy and antibody monotherapy, highlighting the advantages conferred by the engineered platform in terms of lesion-selective accumulation and prolonged intraocular retention (page 11, line 440–444: In the absence of this combination strategy, antibody monotherapy lacks the aforementioned advantages, exhibiting lower lesion-selective accumulation and reduced intraocular retention, thereby limiting its therapeutic duration to roughly 50% of that provided by our engineered platform). With respect to broader efficacy comparisons with other molecules reported in the literature, we note that substantial differences in animal

species, disease models, and experimental designs make direct quantitative comparisons potentially misleading. To maintain scientific rigor and avoid overinterpretation, we have therefore chosen not to include additional cross-study efficacy comparisons in the Discussion. We hope the Reviewer understands our rationale for prioritizing strict comparability to ensure the conclusions drawn are robust and defensible.

We greatly appreciate the insightful suggestion to utilize this strategy for other vitreoretinal indications involving the innate immune activation and macrophage axis. Given the shared pathological basis, diseases like proliferative diabetic retinopathy (PDR) involve a vitreoretinal fibrotic microenvironment analogous to PVR, driven by dysregulated cytokines and macrophages. Since our ACE platform is engineered to specifically neutralize profibrotic cytokines and suppress profibrotic macrophages, it is uniquely positioned to modulate this pathological microenvironment. We are enthusiastic about evaluating this combinatorial strategy in broader clinical contexts, investigating its capacity to mitigate the excessive ECM deposition and membrane contraction inherent to PDR, and thereby prevent the progression to tractional retinal detachment.

Reviewer #3 (Remarks to the Author):

The authors of the manuscript "Therapeutic modulation of the vitreoretinal fibrosis microenvironment using engineered macrophage-derived extracellular vesicles" designed and engineered extracellular vesicles with anti-fibrotic efficacy against proliferative vitreoretinopathy (PVR). These extracellular vesicles were derived from M1 macrophages and were conjugated with a TGFβ1 antibody (and platelet derived growth factor receptor antibody) through MMP-cleavable linkers to treat PVR at an early or advanced stages. The authors used a PVR mouse model and a patient-derived PVR membrane xenograft model to test the therapeutic efficacy of their vesicles in term of in preventing retinal detachment progression. The submitted work presents noteworthy and compelling results that make a clear contribution to the current research field. The results are coherent, internally consistent, and presented within a logically structured framework. It advances existing knowledge through innovative approaches and empirical analysis. The conclusions are supported by the presented data and analyses. The data analysis, interpretation, and conclusions show no major flaws, however, some aspects are listed below.

In summary, the publication makes a valuable contribution to its field and is therefore suitable for publication after revision.

Response: We appreciate the reviewer's positive evaluation. By providing additional experimental data and detailed explanations, we have fully addressed all raised issues, mainly including the definition and identification strategies of different macrophage populations, the incorporation of additional macrophage markers in combination to distinguish between distinct macrophage subsets, further validation of the selective accumulation mechanism of ACE, refinement of multi-omics data visualization, and repeated assessments of therapeutic efficacy, preserving baseline status.

Reviewer questions:

1. Figure 1E: Which markers were used to define the different macrophage populations?

Response: We thank the reviewer for providing us this opportunity to clarify the methodology used in Fig. 1e. In our study, macrophage populations were identified using CIBERSORTx, a well-established and widely validated computational framework for deconvoluting immune cell subsets from bulk RNA-seq data based on gene expression signatures, with the LM22 reference signature matrix, which defines immune cell types according to characteristic multi-gene expression profiles rather than individual markers[4-6].

We have now clarified this methodology in the revised Methods section and added a brief note in the Fig. 1e legend to indicate that macrophage populations were determined by CIBERSORTx analysis of RNA-seq data.

Method:

Page 13, line 535–539: Macrophage phenotypes were estimated using CIBERSORTx. Bulk RNA-seq data were uploaded to the CIBERSORTx platform, and the LM22 signature matrix was applied to deconvolute immune cell fractions. The relative abundance of macrophage subsets, including M0, M1, and M2 phenotypes, was calculated for each sample following the default parameters.

Main text:

Page 26, line 996–997: e Cell type enrichment analysis of the sequencing data via CIBERSORTx: M0 and M2 macrophages were significantly enriched in PVR samples (n = 16 PVR membranes from patients with PVR) when compared to non-PVR samples (n = 7 ILMs from non-PVR donors). Macrophage populations were determined by CIBERSORTx analysis of RNA-seq data.

Reference in response:

4. Newman, A. M., Steen, C. B., Liu, C. L., Gentles, A. J., Chaudhuri, A. A., Scherer, F., Khodadoust, M. S., Esfahani, M. S., Luca, B. A., Steiner, D., Diehn, M., & Alizadeh, A. A. (2019). Determining cell type abundance and expression from bulk tissues with digital cytometry. *Nature biotechnology*, 37(7), 773–782.

5. Hanley, C. J., Waise, S., Ellis, M. J., Lopez, M. A., Pun, W. Y., Taylor, J., Parker, R., Kimbley, L. M., Chee, S. J., Shaw, E. C., West, J., Alzetani, A., Woo, E., Ottensmeier, C. H., Rose-Zerilli, M. J. J., & Thomas, G. J. (2023). Single-cell analysis reveals prognostic fibroblast subpopulations linked to molecular and immunological subtypes of lung cancer. Nature communications, 14(1), 387.

6. Steen, C. B., Luca, B. A., Esfahani, M. S., Azizi, A., Sworder, B. J., Nabet, B. Y., Kurtz, D. M., Liu, C. L., Khameneh, F., Advani, R. H., Natkunam, Y., Myklebust, J. H., Diehn, M., Gentles, A. J., Newman, A. M., & Alizadeh, A. A. (2021). The landscape of tumor cell states and ecosystems in diffuse large B cell lymphoma. Cancer cell, 39(10), 1422–1437.e10.

2. CD206 is not an exclusive M2 marker, as it is also partially expressed by M1 populations. Wouldn't it be reasonable to include additional macrophage markers, such as CD163 and CD68, in combination to distinguish between the different macrophage

populations?

Response: We thank the reviewer for drawing our attention to this issue. To address the reviewer's concern and improve the robustness of our assessment, we have now incorporated additional immunohistochemical staining on consecutive sections using CD163 as an additional M2-associated marker.

First, we performed additional validation to examine the vitreoretinal fibrosis microenvironment in both PVR clinical samples and the PVR mouse model. Consistent with our previous observations using CD206 as a marker for M2 macrophages (Fig. 1i), CD163-positive M2 macrophages showed a similar increase in clinical PVR samples compared with a non-PVR donor eye (Fig. R5a – c), providing additional validation of M2 macrophage enrichment. Similarly, in the mouse model, CD163 expression was significantly increased in PVR membranes relative to those from non-model control (NC) mice (Fig. R5d – f).

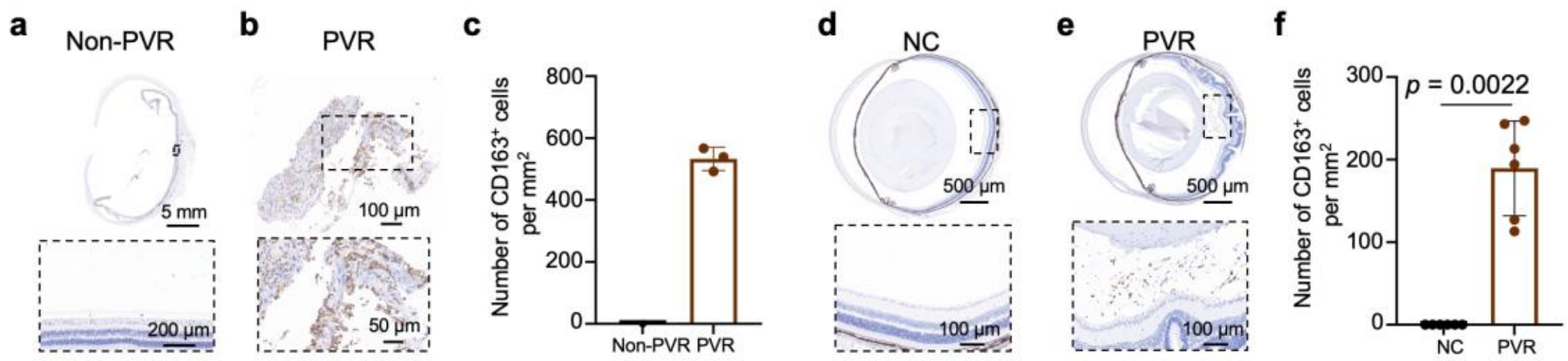

Fig. R5 (fig. S1a–c,e,g,h in revision). Additional characterizations of M2 macrophages, related to Fig. 1h,i, and fig. S1d–g.

a IHC staining of sectioned eyeball from a non-PVR donor, showing the expression of CD163. The outlined area is magnified in the bottom sub-panel.

b IHC staining of sectioned PVR membrane from a PVR patient, showing the expression of CD163. The outlined area is magnified in the bottom sub-panels.

c Quantitative analysis of the number of CD163-positive cells (n = 1 eyeball from a non-PVR donor, n = 3 PVR membranes from PVR patients).

d IHC staining showing the expression of CD163 in retinal slices of NC mice. The images are presented at the same magnification. The outlined areas are magnified in the bottom sub-panels.

e IHC staining showing the expression of CD163 in retinal slices of PVR model mice.

f Quantitative analysis of the number of CD163-positive cells (n = 6, 1 eye from each of 6 mice).

Data in **c** and **f** are presented as mean ± s.d.. Data in **f** were compared using Mann-Whitney U test.

Above results have now been added as fig. S1a – c,e,g,h in the revised manuscript with corresponding figure captions. We have also revised the corresponding statement in the main text and methods.

Main text:

Page 4, line 121, 123: Similar to α-smooth muscle actin (α-SMA) and Collagen1 (fibrotic markers), TGFβ1, CD206, and CD163 (M2 macrophage-associated markers) exhibited significantly higher expression levels in the PVR membrane samples (Fig. 1h,i, and fig. S1a–c). Such upregulation was also validated in PVR model mice induced by intravitreal injection of gas and primary mouse RPE (mRPE) cells (fig. S1d–h)22.

Methods:

IHC analysis of samples from PVR model mice

Page 15, line 588: For IHC analysis, the sections were incubated with primary antibodies targeting TGFβ1, CD206, CD163, MMP2, α-SMA, and Collagen1, using the manufacturer-recommended dilutions.

Second, in evaluating the therapeutic modulation of the vitreoretinal fibrosis microenvironment by ACE, we incorporated CD163 in combination to distinguish M2 macrophage populations. Consistent with our previous observations that ACE most effectively downregulated CD206-positive M2 macrophages (Fig. 4i), the ACE-treated group also induced the greatest reduction in CD163-positive M2 macrophages compared with the PBS group (Fig. R6). Together, these results indicate that ACE effectively modulates the M2 macrophage population within the vitreoretinal fibrosis microenvironment.

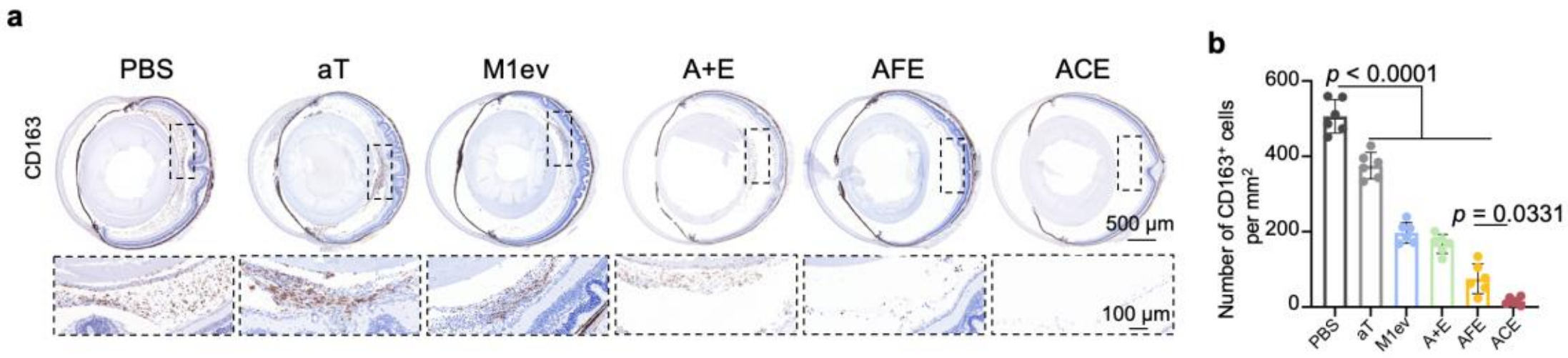

Fig. R6 (fig. S11 in revision). Additional evaluation of therapeutic modulation of the vitreoretinal fibrosis microenvironment after ACE treatment, related to Fig. 4i.

a IHC staining showing the expression of CD163 in retinal slices of PVR model mice of the indicated treatment groups on day 28. The images are presented at the same magnification. The outlined areas are magnified in the bottom sub-panels.

b Quantitative analysis of the number of CD163-positive cells on the PVR membrane

(n = 6, 1 eye from each of 6 mice).

Data in **b** are presented as mean ± s.d. and were compared using one-way ANOVA.

Above results have now been added as fig. S11 in the revised manuscript with corresponding figure captions. We have also revised the corresponding statement in the main text.

Main text:

Page 8, line 301: Consistent with our western blotting results, both the monotherapies and the combination treatments significantly reduced α-SMA levels in myofibroblasts and the number of M2 macrophages compared to the PBS group, with the ACE group conferring the maximal reduction (Fig. 4h,i and fig. S11).

Third, to assess the therapeutic efficacy of aTP-cl-M1ev (ACE^{Plus}) in advanced-stage PVR model mice, CD163 was included as an additional M2 macrophage marker. Consistent with prior observations that ACE^{Plus} strongly downregulated CD206-positive M2 macrophages (Fig. 5k), ACE^{Plus} treatment also decreased CD163-positive M2 macrophages more effectively than ACE, while both treatments resulted in significant reductions compared with PBS (Fig. R7).

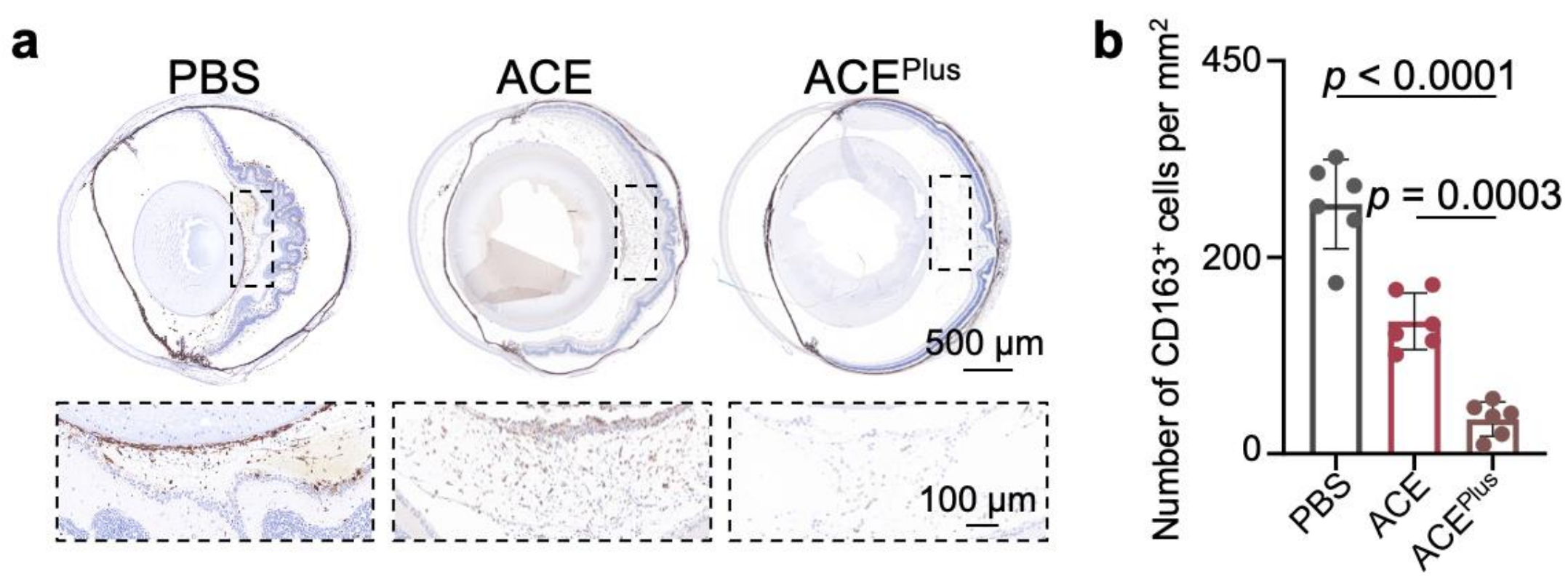

Fig. R7 (fig. S13d,e in revision). Additional anti-fibrotic efficacy of ACE^{Plus}, related to Fig. 5k.

a IHC staining showing CD163 expression in retinal sections from advanced-stage PVR model mice on day 28 following the indicated treatments. The images are presented at the same magnification.

b Quantitative analysis of the number of CD163-positive cells on the PVR membrane (n = 6, 1 eye from each of 6 mice).

Data in **b** are presented as mean ± s.d. and were compared using one-way ANOVA.

Above results have now been added as fig. S13d,e in the revised manuscript with corresponding figure captions. We have also revised the corresponding statement in the main text.

Main text:

Page 9, line 344–345: Subsequently, IHC analysis was performed to further investigate the modulation of the vitreoretinal fibrosis microenvironment and the extent of fibrotic remodeling. Specifically, both ACE and ACE^{Plus} reduced fibroblast and M2 macrophage levels compared to PBS group, with ACE^{Plus} achieving a greater reduction (Fig. 5j,k and fig. S13d,e).

Finally, to assess whether the patient-derived membrane xenograft (PDMX) model faithfully reproduces the key characteristics of the PVR microenvironment from clinical samples, CD163 was included in combination with other markers to evaluate M2 macrophages. Consistent with our previous observations of CD206-positive M2 macrophages (Fig. 6j), CD163-positive M2 macrophages were significantly increased in the eyes of mice in the PDMX model compared with NC mice (Fig. R8).

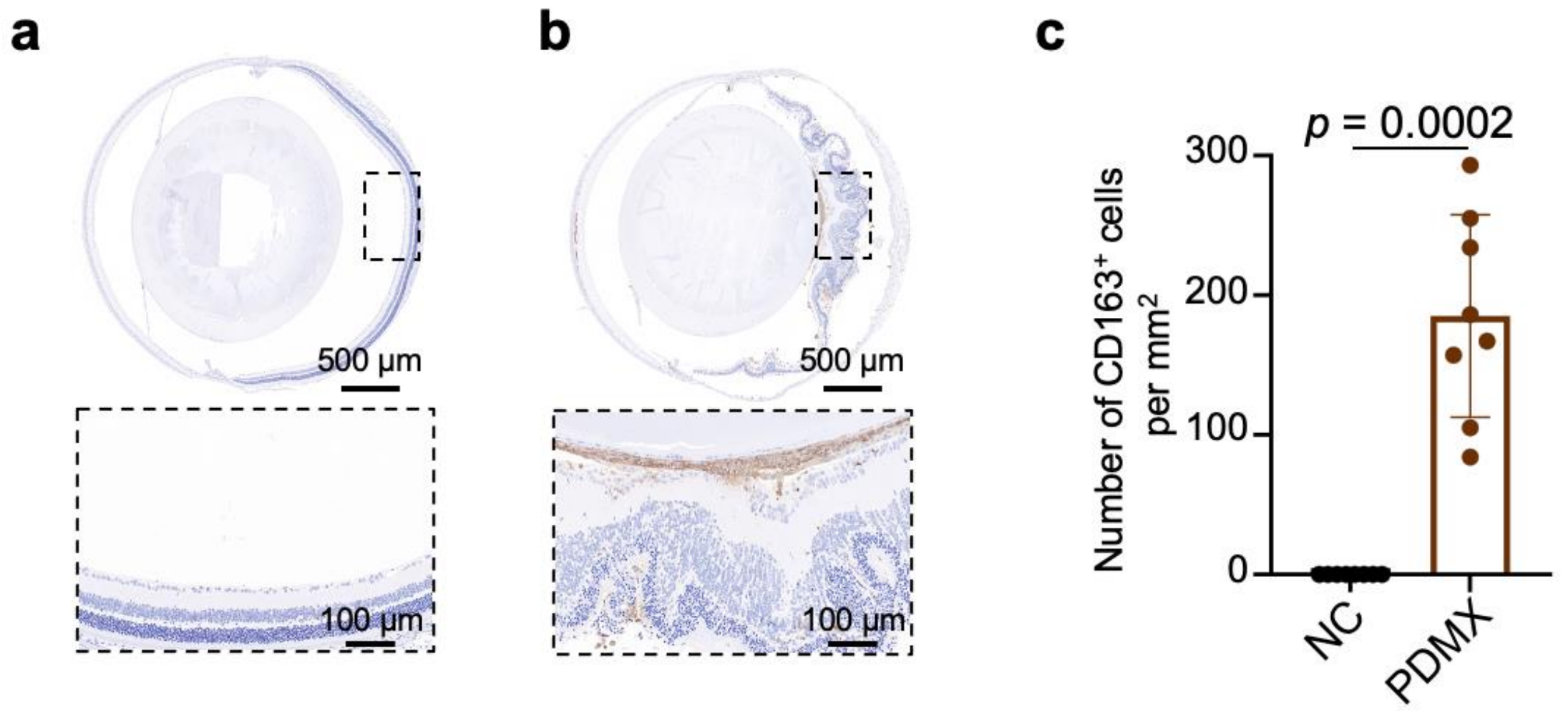

Fig. R8 (fig. S15c–e in revision). Additional characterization of PDMX model mice, related to Fig. 6j.

a IHC staining showing the expression of CD163 in retinal slices of NC mice. The outlined area is magnified in the bottom sub-panel.

b IHC staining showing the expression of CD163 in retinal slices of PDMX mice on day 28. The outlined area is magnified in the bottom sub-panel.

c Quantitative analysis of the number of CD163-positive cells on the PVR membranes (n = 8, 1 eye from each of 8 mice).

Data in **c** are presented as mean ± s.d. and were compared using Mann-Whitney U test.

Above results have now been added as fig. S15c–e in the revised manuscript with corresponding figure captions. We have also revised the corresponding statement in the main text and methods.

Main text:

Page 10, line 389–390: Notably, compared to the non-model control (NC) group, IHC staining showed significantly elevated levels for α-SMA, Collagen1, TGFβ1, M2-associated markers (CD206 and CD163) and MMP2 for the patient-derived PVR membrane (Fig. 6j and fig. S15c–e), supporting that this PDMX model informatively recapitulates key features of the PVR microenvironment.

3. Figure 1P+Q: The visualization is difficult to interpret, as the circle sizes differ only minimally. Introducing a color distinction would make the figure easier to understand.

Response: We thank the reviewer for promoting us to improve the comprehensibility of Fig. 1p,q. Accordingly, we introduced a color distinction and adjusted the visual contrast of the circles in our revised manuscript as follows (Fig. R9).

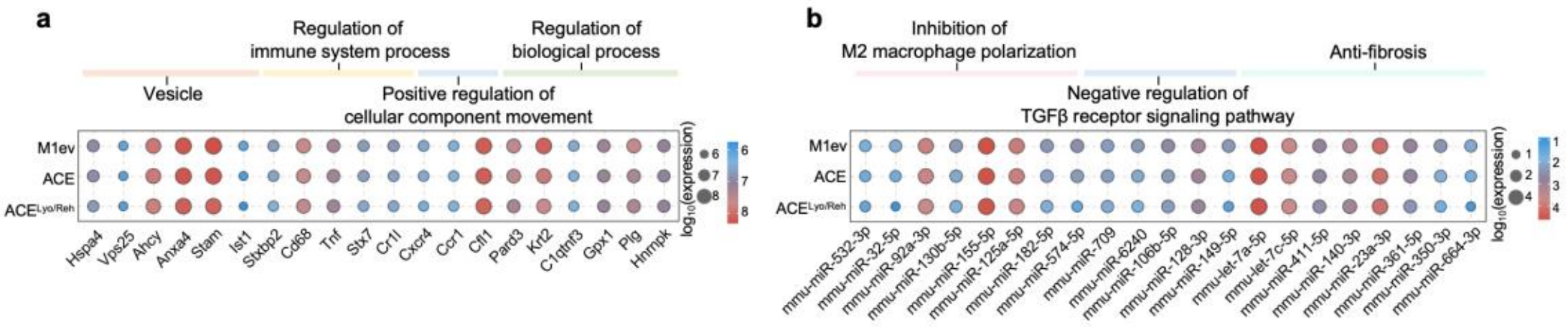

Fig. R9 (replaces Fig. 1p,q in revision). Analysis of the proteome and microRNA transcriptome.

a Proteomics analysis of fresh M1ev, ACE, and $ACE^{Lyo/Reh}$.

b miRNA sequencing of fresh M1ev, ACE, and $ACE^{Lyo/Reh}$.

Above results have now been updated to replace Fig. 1p,q in the revised manuscript.

4. Results Figure 2G: In my opinion, a control using a receptor that is not expressed on ACE would be necessary to clearly demonstrate that the observed effects are mediated by CCR1 and CXCR4.

Response: We thank the reviewer for bringing this to our attention. Regarding the suggestion to clearly demonstrate that the observed effects are mediated by CCR1 and CXCR4, we initially recognized that including a control using a receptor not expressed on ACE would be meaningful. However, performing gene knockout in macrophages is

technically very challenging, particularly in primary macrophages[7,8]. Therefore, we chose a blocking strategy to verify the potential mechanism underlying this selective accumulation.

To address the reviewer's concern that a non-expressed receptor control is needed to confirm CCR1 and CXCR4 mediation, we generated stable knockout mouse macrophage cell lines (Fig. R10a,c). Using extracellular vesicles derived from these cells, we then conducted experiments with single knockouts of CCR1 or CXCR4, as well as double knockouts of both receptors (Fig. R10b,d), to further explore whether the observed effects are mediated by CCR1 and CXCR4 (Fig. R10e,f).

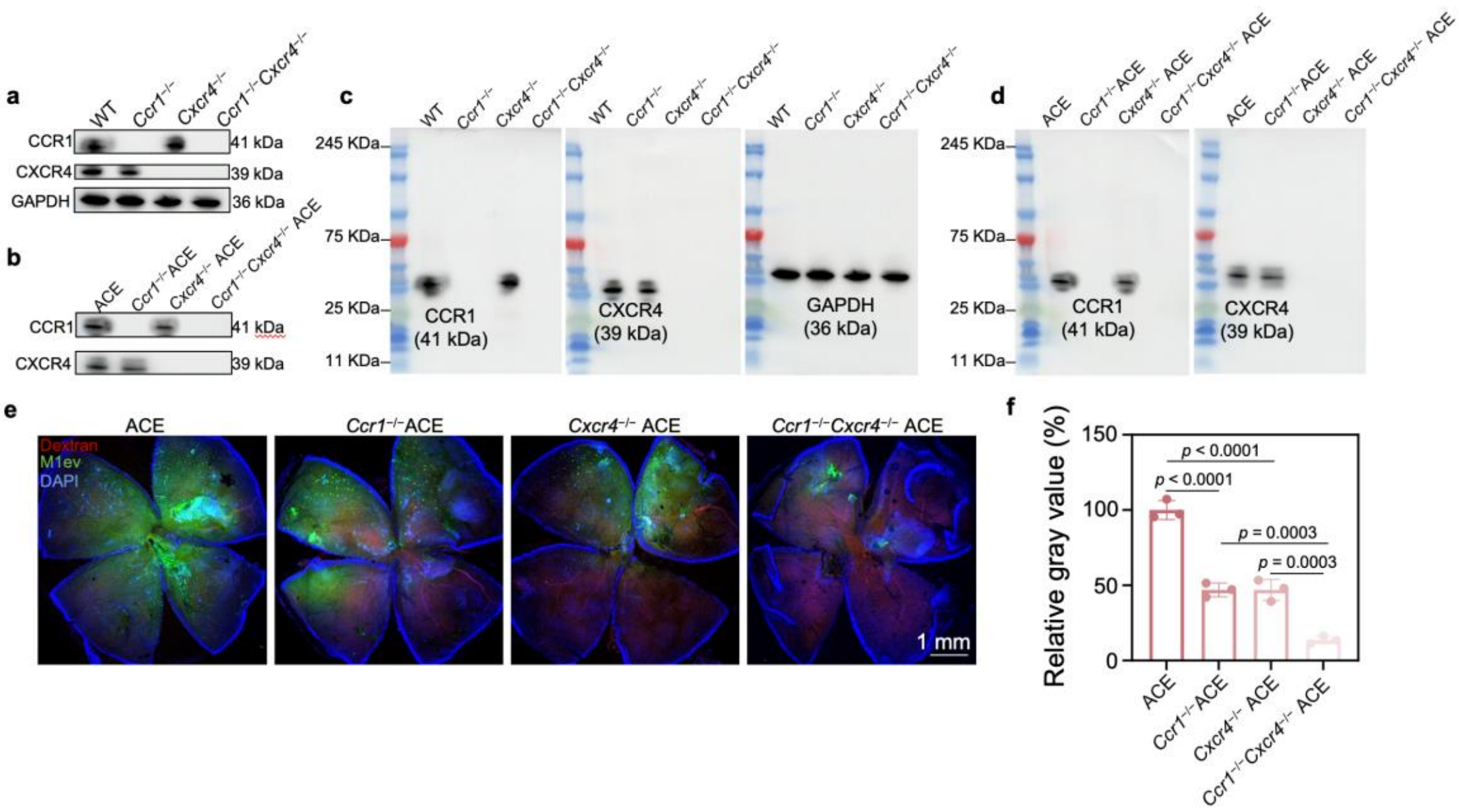

Fig. R10 (fig. S4b–g in revision). Additional characterization of the selective accumulation of ACE.

a Western blot analysis of the expression of CCR1 and CXCR4 in M1 macrophages, including wild-type (WT), Ccr1$^{-/-}$, Cxcr4$^{-/-}$, and double-knockout (Ccr1$^{-/-}$Cxcr4$^{-/-}$) groups.

b Western blot analysis of the expression of CCR1 and CXCR4 in ACE, including ACE, Ccr1$^{-/-}$ACE, Cxcr4$^{-/-}$ACE, and Ccr1$^{-/-}$Cxcr4$^{-/-}$ACE groups.

c Original western blotting images of Fig. R10a.

d Original western blotting images of Fig. R10b.

e Representative images and quantification of the accumulation of M1ev in flat-mounted retinas of PVR model mice on day 1 after indicated treatment (n = 3, 1 eye from each of 3 mice; green, PKH67-labelled M1ev; red, Dextran-labelled retinal vessels; blue, DAPI-labelled cell nuclei). The images are presented at the same magnification.

f Quantification of the accumulation of M1ev in flat-mounted retinas of PVR model

mice

Data in **f** are presented as mean ± s.d. and were compared using one-way ANOVA.

Above results have now been added as fig. S4b–g in the revised manuscript with corresponding figure captions. We have also revised the corresponding statement in the main text and methods.

Reference in response:

7. Chen, Y., Chen, X., Zhang, Y., Wang, M., Yang, M., Wang, R., Yan, X., Shao, S., Xin, H., Hu, Q., Wei, W., & Ping, Y. (2025). Macrophage-specific in vivo RNA editing promotes phagocytosis and antitumor immunity in mice. Science translational medicine, 17(781), eadl5800.

8. Xi, W., Xu, Y., Bao, W., Ding, Z., Xiao, H., Yang, J., Yan, X., & Ping, Y. (2025). In vivo chemogenetic RNA editing of macrophages by bioengineered viruses for sepsis treatment. Nature communications, 10.1038/s41467-025-67655-y. Advance online publication.

Main text:

Page 6, line 209–210: Taking ACE for an example, M1ev levels decreased by 61.5%, 61.8% and 86.8% in the ACE+anti-CCR1, ACE+anti-CXCR4, and ACE+anti-CCR1/CXCR4 groups, respectively (Fig. 2g). Similarly, a consistent effect was observed in the gene knockout setting (fig. S4b–g). Together, these findings confirm that the accumulation to PVR lesions is primarily mediated by CCR1 and CXCR4.

Methods

Page 18, line 714–727: We employed the CRISPR-U™ system from Ubigene to generate CCR1 and/or CXCR4 knockout models in RAW 264.7 murine macrophages. Gene-specific sgRNA pairs were designed to delete key coding regions within the target genes (194 bp for CCR1; 128 bp for CXCR4). For transfection, cells were electroporated with ribonucleoprotein (RNP) complexes consisting of Cas9 protein and the respective sgRNA(s). Following transfection, cells were subjected to limited dilution to isolate single-cell clones. Knockout efficacy was confirmed by PCR amplification of the target loci followed by Sanger sequencing analysis of the monoclonal populations. Double-knockout cells were generated by co-delivering both CCR1 and CXCR4 sgRNA pairs during the RNP electroporation step, with subsequent clonal screening verifying biallelic disruption of both genes. We next isolated extracellular vesicles from the aforementioned gene-edited cells using the same

procedures as described above and performed retinal flat-mount imaging to quantify the selective accumulation of PKH67-labeled M1ev in lesion areas, following the same experimental paradigm.

5. Figure 3A+B and Figure 5D: It would be helpful to have representative fundus and OCT images from day 0 to assess the extent of PVR at the start of therapy.

Response: We thank the reviewer for drawing our attention to this issue. In response to this suggestion, we repeated the fundus and optical coherence tomography (OCT) experiments corresponding to Fig. 3a,b (Fig. R11) and Fig. 5d (Fig. R12), preserving the baseline status at the time of treatment.

Regarding Fig. 3a,b, at the time of treatment initiation, all mice showed cellular aggregates in the vitreous with an average PVR grade of 1 (Fig. R11a,b). On day 28 after intravitreal injection of indicated treatments (at the endpoint), ACE markedly reduced PVR severity compared with PBS (Fig. R11c,d), consistent with our previous observations.

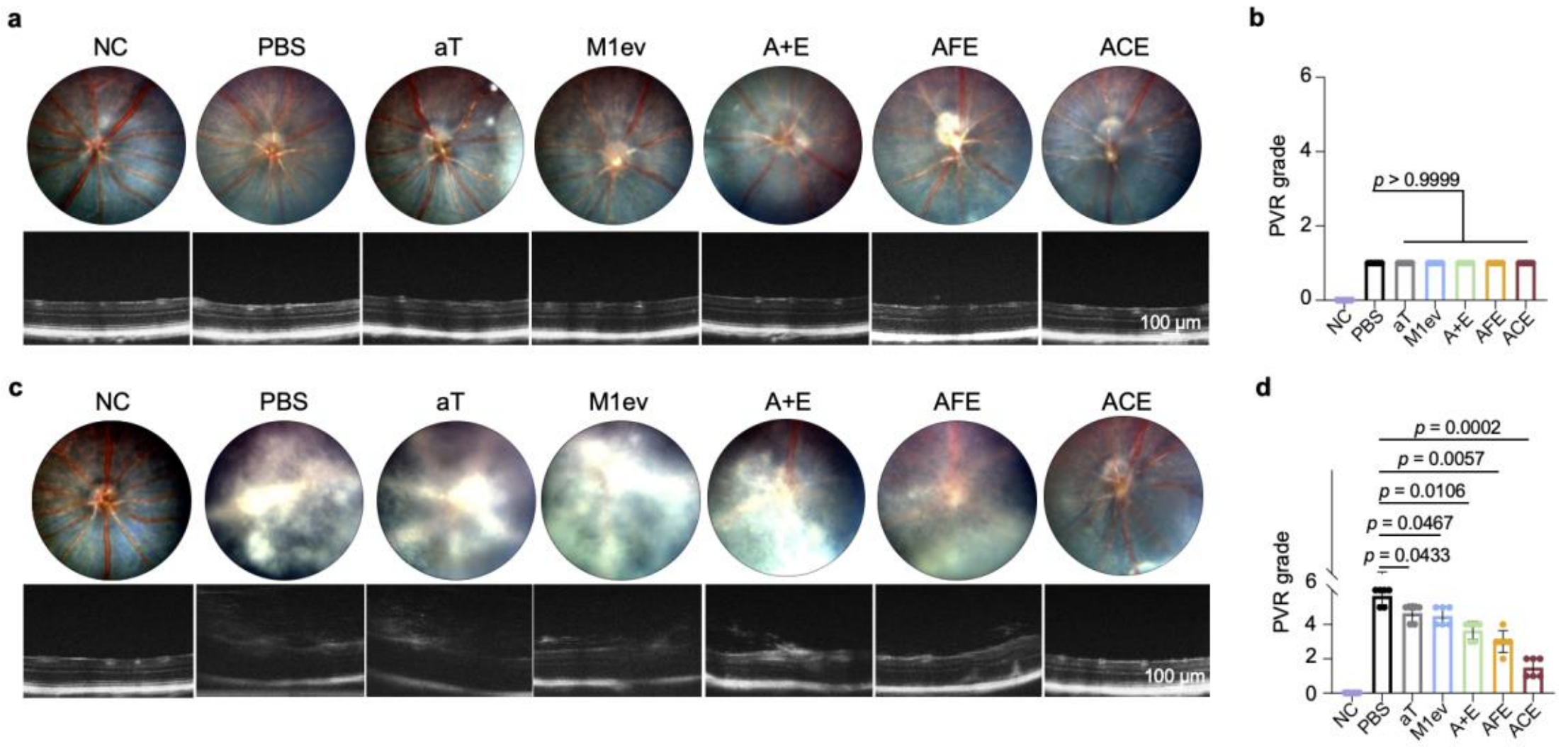

Fig. R11 (a and b as revised fig. S6a,b; c and d as revised Fig. 3a,b,g). Additional anti-fibrotic efficacy of ACE, related to Fig. 3.

a Representative fundus (top) and OCT (bottom) images of NC mice and PVR model mice, assessing PVR severity at the time of treatment initiation. The images are presented at the same magnification.

b Quantitative analysis of the PVR grade, performed based on clinical assessment, fundus, and OCT; table S2 for grade criteria (n = 6, 1 eye from each of 6 mice).

c Representative fundus (top) and OCT (bottom) images of NC mice and PVR model mice, assessing therapeutic efficacy on day 28 after intravitreal injection of indicated treatments. The images are presented at the same magnification.

d Quantitative analysis of the PVR grade, performed based on clinical assessment,

fundus, and OCT; table S2 for grade criteria ($n = 6$, 1 eye from each of 6 mice).
Data in **b** and **d** are presented as mean ± s.d. and were compared using one-way ANOVA.

The above results have now been incorporated into the revised manuscript as fig. S6a,b and Fig. 3a,b,g, with corresponding figure captions. We have also revised the corresponding statement in the main text.

Main text:

Page 6, line 227: As shown in Fig. 3a–c and fig. S6a–e, color fundus photography (CFP), optical coherence tomography (OCT), and H&E staining revealed that mice treated with PBS showed a central hypopigmented area corresponding to the optic nerve, along with a large PVR membrane infiltrated by numerous cells and extensive RD.

Regarding Fig. 5d, at the time of treatment initiation, all mice exhibited early epiretinal membrane formation and some retinal damage, with average PVR grades around 4 (Fig. R12a–c). Consistent with our previous observations, at the endpoint, ACE^{Plus} effectively attenuated the major pathological processes of advanced PVR, demonstrating superior therapeutic efficacy (Fig. R12d–f).

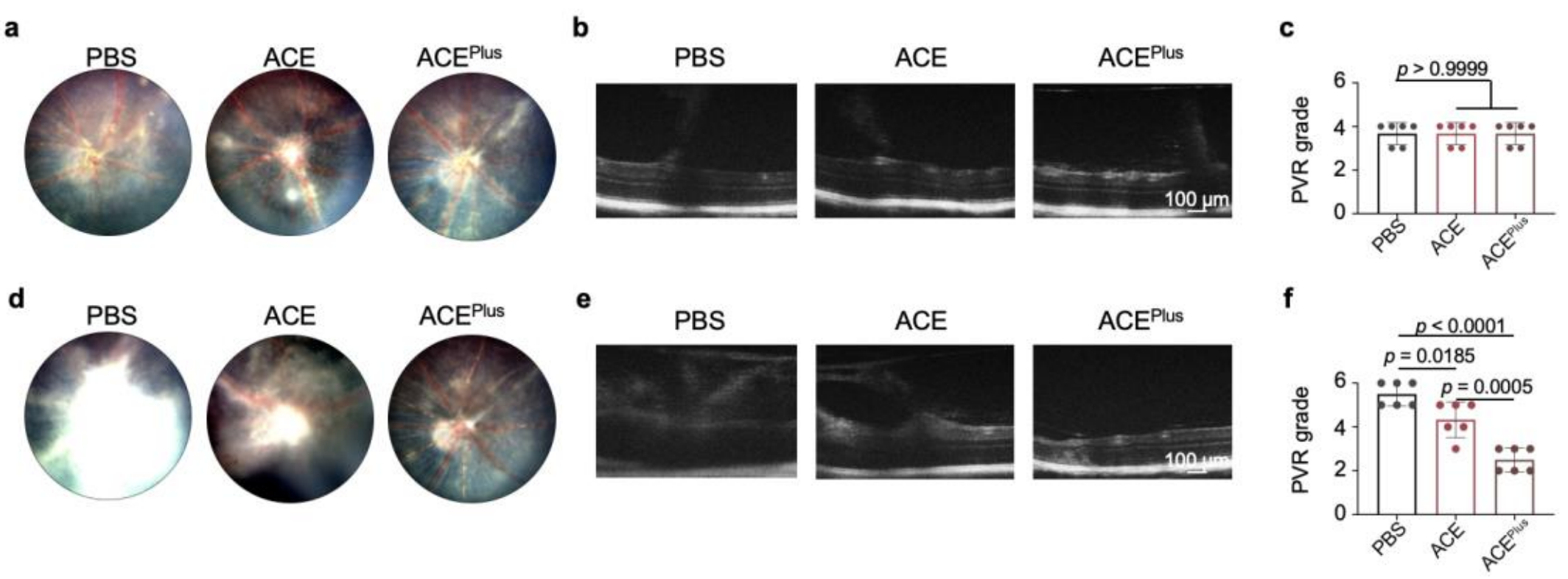

Fig. R12 (a–c as revised fig. S13a–c; d–f as revised Fig. 5d,i). Additional anti-fibrotic efficacy of ACE^{Plus}.

a,b Representative fundus (a) and OCT images (b) assessing PVR severity in advanced-stage model mice at the time of treatment initiation. The images are presented at the same magnification.

c Quantitative analysis of the PVR grade, performed based on clinical assessment, fundus, and OCT; table S2 for grade criteria ($n = 6$, 1 eye from each of 6 mice).

d,e Representative fundus (d) and OCT images (e) assessing the therapeutic efficacy in advanced-stage PVR model mice on day 28 following intravitreal injection of the

indicated treatments. The images are presented at the same magnification.

f Quantitative analysis of the PVR grade, performed based on clinical assessment, fundus, and OCT; table S2 for grade criteria ($n = 6$, 1 eye from each of 6 mice).

Data in **c** and **f** are presented as mean ± s.d. and were compared using one-way ANOVA.

The above results have now been incorporated into the revised manuscript as fig. S13a–c and Fig. 5d,i, with corresponding figure captions. We have also revised the corresponding statement in the main text.

Main text:

Page 8, line 331: For the assessment of ACE^{Plus} at an advanced PVR mouse model, intravitreal injections (PBS, ACE, or ACE^{Plus}) were administered on day 7 after the mRPE injection, and evaluations of CFP, OCT, and H&E staining were conducted on day 28 (Fig. 5d,e and fig. S13a–c).

6. In some sections of the Materials and Methods, critical details are missing. For example, the number of cells in the single-cell suspension used for xenotransplantation, the exact storage conditions for the vesicles, and the parameters for ultracentrifugation are not specified. This section should be revised to ensure accuracy and reproducibility.

Response: We thank the reviewer for drawing attention to this point. Accordingly, we have revised this section to add the relevant experimental details, including the number of cells used for single-cell suspension in xenotransplantation and the storage conditions of the vesicles. The ultracentrifugation parameters were described in the original manuscript (page 15, line 615–616: “The clarified fluid was subsequently centrifuged at 100,000 g for 70 minutes to pellet the extracellular vesicles”) and have now been made more clearly identifiable for the reader. For clarity, several representative examples of these revisions are briefly outlined below.

Methods

Page 14, line 577: These membranes were enzymatically digested into single-cell suspensions, and 1×10^4 cells were used for xenotransplantation.

Page 15, line 618–619: The final extracellular vesicle preparations were resuspended in PBS and stored at 4 °C for short-term use (within 1 week) or at -80 °C for long-term storage prior to downstream applications. All samples were processed consistently following standardized procedures.

Page 15, line 615: The clarified fluid was subsequently subjected to ultracentrifugation at 100,000 g for 70 minutes to pellet the extracellular vesicles.

Reviewer #4 (Remarks to the Author):

This manuscript explores engineered M1-macrophage-derived EVs (ACE/ACE^{Plus}) delivering anti-TGFβ1 and anti-PDGFRα antibodies via MMP-cleavable linkers to treat proliferative vitreoretinopathy (PVR). The work is innovative and clinically meaningful, especially with the introduction of a patient-derived xenograft PVR model that increases translational value.

However, several key mechanistic and methodological issues need to be resolved before the therapeutic claims can be supported.

Response: We sincerely thank the reviewer for their thorough assessment of our manuscript and for recognizing the clinical relevance of the patient-derived xenograft PVR model. We appreciate the constructive guidance provided regarding the mechanistic characterization, inflammatory safety, and biodistribution quantifications of our engineered extracellular vesicles (ACE).

In formulating our response strategy, we have strictly adhered to the guidance provided by the editor in the decision letter:

"We wish to emphasize that, from an editorial perspective, we do not find that additional mechanistic data are essential (R4). However, it is important that the limitations of this aspect of your study are clearly acknowledged and discussed. Conversely, given the potential clinical relevance of your work, we consider it essential that further data be provided regarding inflammatory safety (R1 and R4) and biodistribution quantifications."

Driven by this editorial directive, we have structured our revision to prioritize the essential safety and biodistribution data (Major Comments 2 & 3). For the mechanistic questions (Major Comment 1), we have provided a detailed rationale for the experimental design choices and explicitly discussed the study limitations in the revised text.

1) Mechanistic evidence remains associational

The impact of ACE is described primarily by static marker reduction (α-SMA, p-Smad2/3, Arg1, STAT6) [Fig. 4E – F], without direct demonstration that:

- Effects are M2-macrophage-dependent
- Spatiotemporal MMP2-mediated linker cleavage drives efficacy
- TGF-β suppression causally prevents myofibroblast transition

Proposed experiments:

• Macrophage depletion or polarization-rescue studies

• MMP2 inhibition in vivo confirming linker necessity

• Functional macrophage assays (migration/phagocytosis/cytokine secretion)

Response: We thank the reviewer for reminding us of these issues. Consistent with the editor's guidance that extensive new mechanistic data might be not essential for this revision, we have addressed these points by clarifying the biological rationale for omitting the depletion study and by reinforcing the alternative lines of evidence, while also incorporating additional experiments to bolster our findings.

(i) Effects are M2-macrophage-dependent

While we acknowledge that macrophage depletion or polarization-rescue studies would, in principle, validate the M2-dependency of ACE, performing global depletion (e.g., via clodronate liposomes[9,10]) in our proliferative vitreoretinopathy (PVR) model presents fundamental biological constraints that render such approaches scientifically infeasible. Specifically, since macrophages are known drivers of PVR[11], their depletion is expected to attenuate disease severity. This compromised baseline prevents a valid assessment of ACE's therapeutic efficacy, rendering the proposed polarization-rescue studies methodologically impractical. Moreover, given the lack of strategies with sufficient specificity to exclusively deplete the fibrosis-driving M2 subset[12,13], a global strategy would indiscriminately eliminate M1 populations essential for physiological functions [14,15], introducing non-specific pathological injuries that would inevitably confound data interpretation rather than clarifying the mechanism.

Our original data provide converging lines of evidence supporting the M2-dependency of ACE. Specifically, data mining identified M2 macrophages as the predominant immune population in PVR, a finding we extensively corroborated in both clinical samples and our mouse model (Fig. 1e,h,i and fig. S1). Mechanistically, ACE treatment significantly downregulated signaling pathways and specific markers governing M2 polarization (Fig. 4a,b,f). Notably, this molecular suppression of M2 macrophages (Fig. 4i) was accompanied by the restoration of retinal structure and visual function (Fig. 3h–j). Collectively, the strict correlation between M2 suppression and phenotypic improvement underscores M2-dependency of ACE efficacy.

(ii) Spatiotemporal MMP2-mediated linker cleavage drives efficacy

Our original data demonstrated the superior therapeutic efficacy of ACE compared

to AFE (the non-cleavable linker) (Fig. 3), indicating that linker cleavage facilitates the efficient release and bioavailability of aT. To specifically address the point regarding *in vivo* MMP2 inhibition experiments for confirming linker necessity, we conducted an additional experiment utilizing fundus and OCT imaging, along with H&E analysis, on day 28 following intravitreal injection of ACE with or without an MMP2 inhibitor. Consistent with the lack of efficacy observed with the non-cleavable AFE, these analyses revealed that pharmacological inhibition of MMP2 significantly diminished the therapeutic efficacy of ACE (Fig. R13). Together, these complementary findings support that MMP2-mediated cleavage drives the therapeutic bioactivity of ACE.

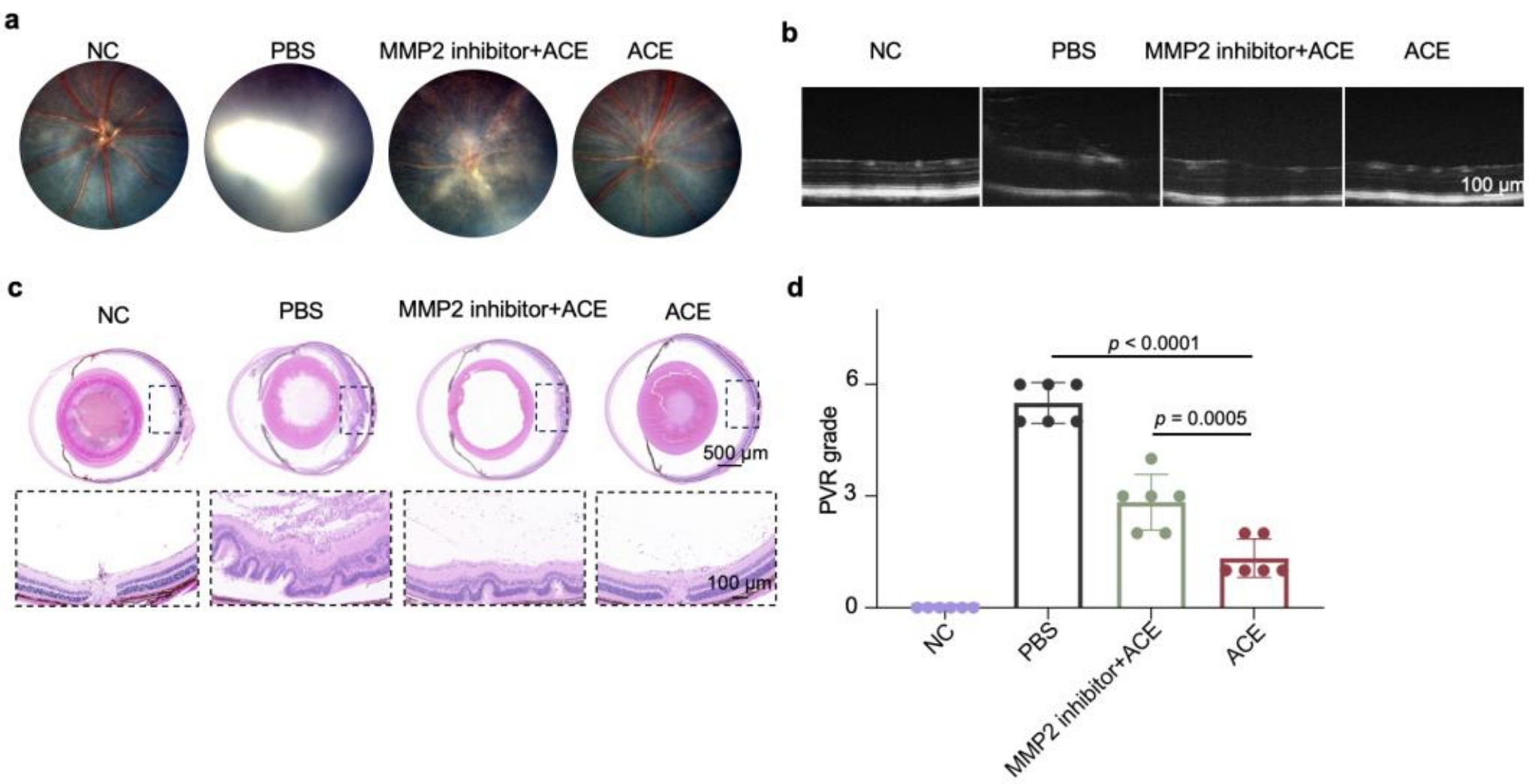

Fig. R13 (fig. S6c–f in revision). Evaluation of the role of MMP2-mediated linker cleavage *in vivo*.

a,**b** Representative fundus (a) and OCT (b) images of NC mice and PVR model mice, assessing the role of MMP2-mediated linker cleavage *in vivo* on day 28 after intravitreal injection of indicated treatments. The images are presented at the same magnification.

c H&E staining of retinal slices from PVR model mice of indicated treatment groups on day 28. The images are presented at the same magnification. The outlined areas are magnified in the bottom sub-panels.

d Quantitative analysis of the PVR grade, performed based on clinical assessment, fundus images, OCT images, and H&E staining; table S2 for grade criteria (n = 6, 1 eye from each of 6 mice).

Data in **d** are presented as mean ± s.d. and were compared using one-way ANOVA.

(iii) TGF-β suppression causally prevents myofibroblast transition

Since TGFβ1 is the established master regulator driving myofibroblast transition

in PVR[16,17], ACE is designed to disrupt this process through a dual-targeting mechanism: directly neutralizing TGFβ1 via aT and indirectly cutting off its supply by suppressing M2 macrophages. Consistent with this design, transcriptomic and proteomic analyses demonstrated that ACE suppressed core TGFβ components (Tgfb1, Tgfbr2) and its downstream canonical signaling (p-Smad2/3) significantly more potently than aT or M1ev monotherapies (Fig. 4b,e). This molecular blockade was mirrored by the prevention of myofibroblast activation (reduced α-SMA and ECM production) (Fig. 4g,h,j), providing mechanistic evidence that ACE prevented fibrosis by blocking the TGFβ/Smad signaling axis essential for myofibroblast differentiation.

In the revised manuscript, we have acknowledged and discussed the limitations regarding the associational nature of our mechanistic evidence within the relevant contexts of the "Therapeutic modulation of the vitreoretinal fibrosis microenvironment" section, particularly concerning the direct demonstration that effects are M2-macrophage-dependent and that TGFβ suppression causally prevents myofibroblast transition.

In addition, Fig. R13 has now been incorporated into the revised manuscript as fig. S6c–f, with corresponding figure captions. We have also revised the corresponding statement in the main text.

Main text:

Page 7-8, line 288-293: The TGFβ–SMAD pathway has been previously implicated in myofibroblast activation[36], including in the context of retinal pathology[37]. We examined the levels of multiple TGFβ–SMAD pathway components in retinal tissues, including α-SMA, TGFβ1, Smad2/3, and p-Smad2/3[38,39]. Compared to the PBS group, both the monotherapies (aT and M1ev) and the combination treatments (A+E, AFE, and ACE) showed reduced expression levels of all measured proteins, with the ACE group exhibiting the most significant reduction (Fig. 4e and fig. S8). Given that multiple DEGs from our transcriptome analysis are known to function in M2 macrophage polarization, we detected consistent decreasing trends across the six groups for proteins including IL4Rα, IL13Rα1, Stat6, p-Stat6, and Arg1 (Fig. 4f and fig. S9a,b)[40–44], supporting an impact from inhibited M2 macrophage polarization in the observed ACE therapeutic benefits on retinal pathology. Notably, we also found that the levels of fibronectin (FN1) and Collagen1 (both included in the ECM–receptor interaction gene set) showed similar decreased trend across the six groups (Fig. 4g and fig. S9c,d), lending additional support to anti-fibrotic impacts from each ACE components. <u>Collectively, the convergence of these findings provides support for our rationale that</u>

inhibiting M2 macrophage polarization and neutralizing TGFβ1 drive the therapeutic efficacy. Establishing a direct causal link to confirm that the benefits are strictly M2-macrophage-dependent and that TGFβ1 suppression is the direct driver preventing myofibroblast transition would necessitate future investigations using cell-specific genetic models.

Page 6, line 227, 234–235: As shown in Fig. 3a–c and fig. S6a–e, color fundus photography (CFP), optical coherence tomography (OCT), and H&E staining revealed that mice treated with PBS showed a central hypopigmented area corresponding to the optic nerve, along with a large PVR membrane infiltrated by numerous cells and extensive RD. After treatments, the number of infiltrating cells, the area of PVR membrane, the extent of RD, and the PVR grade gradually decreased in the sequence of monotherapies (aT and M1ev) and the combination treatments (A+E, AFE, and ACE) (Fig. 3d–g). Compared with PBS group, the PVR grade in group ACE were reduced by 3.8-fold, whereas this therapeutic efficacy was significantly attenuated by MMP2 inhibition (fig. S6f). Note that the area of PVR membrane formation and the extent of RD were comparable to those observed in the non-model control (NC) group, suggesting therapeutic potential in suppressing the progression of vision-threatening PVR.

Reference in response:

9. Xie, M., Lin, Z., Ji, X., Luo, X., Zhang, Z., Sun, M., Chen, X., Zhang, B., Liang, H., Liu, D., Feng, Y., Wang, Y., Li, Y., Liu, B., Huang, W., & Xia, L. (2023). FGF19/FGFR4-mediated elevation of ETV4 facilitates hepatocellular carcinoma metastasis by upregulating PD-L1 and CCL2. Journal of hepatology, 79(1), 109–125.

10. Sung, C. Y. W., Hayase, N., Yuen, P. S. T., Lee, J., Fernandez, K., Hu, X., Cheng, H., Star, R. A., Warchol, M. E., & Cunningham, L. L. (2024). Macrophage depletion protects against cisplatin-induced ototoxicity and nephrotoxicity. Science advances, 10(30), eadk9878.

11. Pastor, J. C., Rojas, J., Pastor-Idoate, S., Di Lauro, S., Gonzalez-Buendia, L., & Delgado-Tirado, S. (2016). Proliferative vitreoretinopathy: A new concept of disease pathogenesis and practical consequences. Progress in retinal and eye research, 51, 125–155.

12. Xu, Y., Ying, L., Lang, J. K., Hinz, B., & Zhao, R. (2024). Modeling mechanical activation of macrophages during pulmonary fibrogenesis for targeted anti-fibrosis therapy. Science advances, 10(13), eadj9559.

13. Runtsch, M. C., Angiari, S., Hooftman, A., Wadhwa, R., Zhang, Y., Zheng, Y., Spina,

J. S., Ruzek, M. C., Argiriadi, M. A., McGettrick, A. F., Mendez, R. S., Zotta, A., Peace, C. G., Walsh, A., Chirillo, R., Hams, E., Fallon, P. G., Jayamaran, R., Dua, K., Brown, A. C., O'Neill, L. A. J. (2022). Itaconate and itaconate derivatives target JAK1 to suppress alternative activation of macrophages. Cell metabolism, 34(3), 487–501.e8.
14. Chen, D., Xie, J., Fiskesund, R., Dong, W., Liang, X., Lv, J., Jin, X., Liu, J., Mo, S., Zhang, T., Cheng, F., Zhou, Y., Zhang, H., Tang, K., Ma, J., Liu, Y., & Huang, B. (2018). Chloroquine modulates antitumor immune response by resetting tumor-associated macrophages toward M1 phenotype. Nature communications, 9(1), 873.
15. van Elsas, M. J., Middelburg, J., Labrie, C., Roelands, J., Schaap, G., Sluijter, M., Tonea, R., Ovcinnikovs, V., Lloyd, K., Schuurman, J., Riesenfeld, S. J., Gajewski, T. F., de Miranda, N. F. C. C., van Hall, T., & van der Burg, S. H. (2024). Immunotherapy-activated T cells recruit and skew late-stage activated M1-like macrophages that are critical for therapeutic efficacy. Cancer cell, 42(6), 1032–1050.e10.
16. Hoerster R, Muether PS, Vierkotten S, Hermann MM, Kirchhof B, Fauser S. Upregulation of TGF-ß1 in experimental proliferative vitreoretinopathy is accompanied by epithelial to mesenchymal transition. Graefes Arch Clin Exp Ophthalmol. 2014 Jan;252(1):11-6.
17. Yang, S., Li, H., Yao, H., Zhang, Y., Bao, H., Wu, L., Zhang, C., Li, M., Feng, L., Zhang, J., Zheng, Z., Xu, G., & Wang, F. (2021). Long noncoding RNA ERLR mediates epithelial-mesenchymal transition of retinal pigment epithelial cells and promotes experimental proliferative vitreoretinopathy. Cell death and differentiation, 28(8), 2351–2366.

2) Inflammatory safety of M1-EVs is not addressed
M1 EVs may induce innate inflammatory activation. Only gross histology and IOP are reported.

Proposed additions:

- Microglia activation markers (IBA1, C3, TMEM119)
- Inflammatory cytokines (TNF-α, IL-1β, IL-6)
- Comparison vs naïve EVs (M0-EVs)

Response: We thank the reviewer for bringing this important point to our attention. To evaluate this, C57BL/6 mice were intravitreally injected with ACE or engineered extracellular vesicles derived from naïve macrophages (ACE-M0). On day 28 post-injection, we assessed retinal microglial status using a panel of markers (IBA1, TMEM119, and C3) and quantified inflammatory cytokines (TNF-α, IL-1β, and IL-6)

in ocular fluids.

As shown in Fig. R14, the ACE-treated group exhibited no detectable signs of innate immune activation compared to the PBS or ACE-M0 control groups. Specifically, we observed no significant upregulation of microglial activation markers or pro-inflammatory cytokines within the eye. We attributed this absence of aberrant immune activation to two main aspects. On the one hand, we utilized an optimized therapeutic dose established via dose-escalation studies (fig. S5), thereby avoiding the administration of excessive extracellular vesicles. On the other hand, the normal vitreoretinal microenvironment lacked the pathological ligand expression, which limited receptor-mediated selective accumulation. Collectively, these factors likely minimized non-specific microglial uptake and subsequent activation.

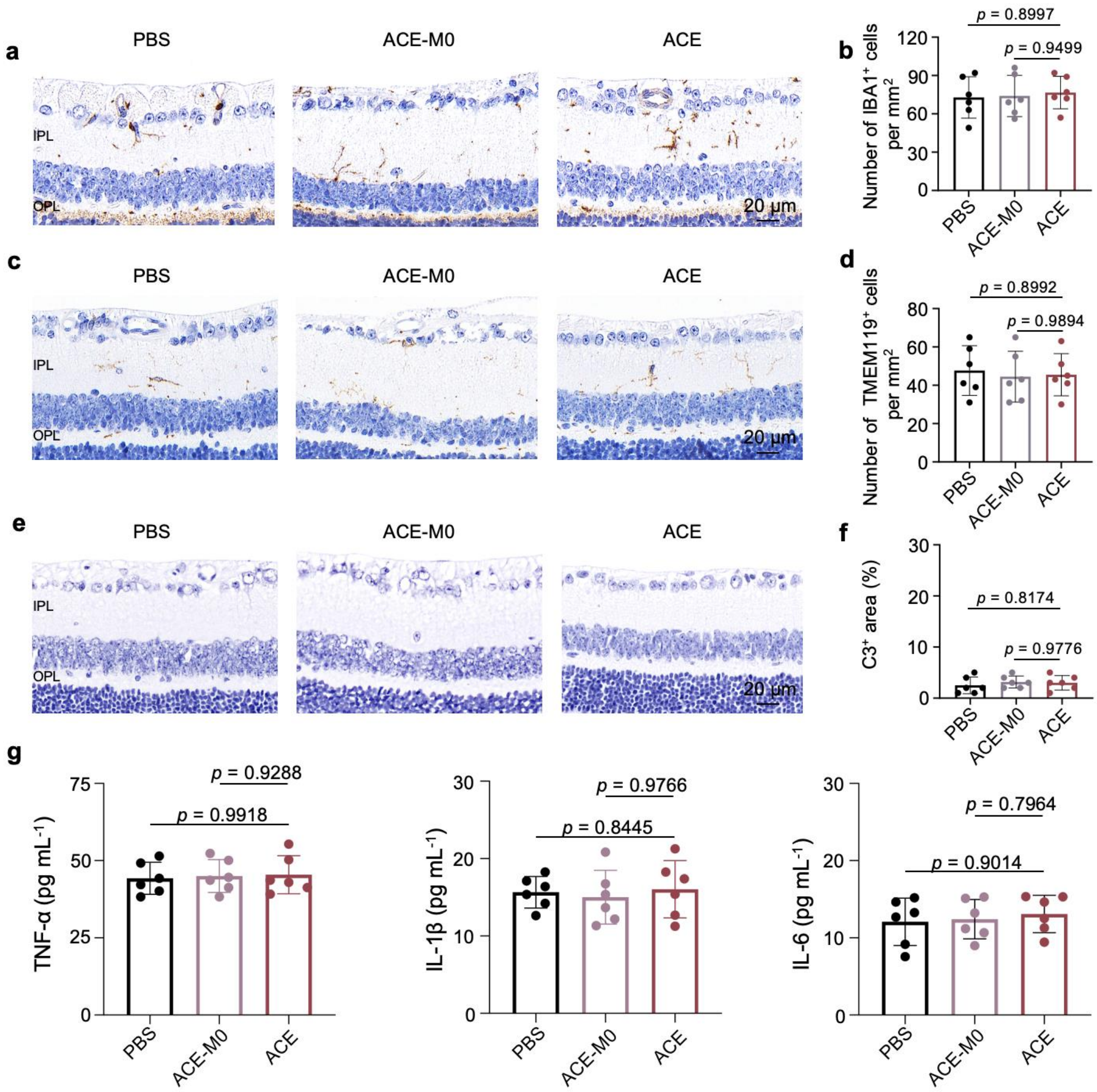

Fig. R14. Biosafety evaluation of ACE in C57BL/6 mice.

a IHC staining showing the expression of IBA1 in retinal slices of C57BL/6 mice of the indicated treatment groups on day 28. The images are presented at the same magnification.

b Quantitative analysis of the number of IBA1-positive cells (*n* = 6, 1 eye from each of 6 mice).

c IHC staining showing the expression of TMEM119 in retinal slices of C57BL/6 mice of the indicated treatment groups on day 28. The images are presented at the same magnification.

d Quantitative analysis of the number of TMEM119-positive cells (*n* = 6, 1 eye from each of 6 mice).

e IHC staining showing the expression of C3 in retinal slices of C57BL/6 mice of the indicated treatment groups on day 28. The images are presented at the same magnification.

f Quantitative analysis of the C3-positive area (*n* = 6, 1 eye from each of 6 mice).

g Quantification of inflammatory cytokines (TNF-α, IL-1β, and IL-6) concentrations in indicated treatment by ELISA assay.

Data in **b**,**d**,**f**,**g** are presented as mean ± s.d. and were compared using one-way ANOVA.

3) Biodistribution and bioavailability

The authors state that aT accumulates at the lesion site (Fig. 2H), but no quantitative biodistribution is provided.

• Such as retina vs vitreous vs RPE/choroid compartments and whether EVs cross ILM into preretinal fibrosis.

Response: We thank the reviewer for raising these issues. In response, we performed a quantitative statistical analysis of the fluorescence intensity based on the images in Fig. 2h (Fig. R15a), showing that the AFE and ACE groups exhibit a significantly higher aT signal at the lesion site compared to the A+E group, attributable to M1ev-mediated selective accumulation of aT at the lesion site versus the rapid ocular clearance of free aT.

To further spatially resolve this biodistribution across ocular compartments as requested, we quantified the partitioning of aT specifically within the vitreous cavity, the neurosensory retina, and the RPE/choroid layers. Our analysis reveals that aT is predominantly enriched at the vitreoretinal interface within the posterior vitreous, consistent with the pathological characteristics of the PVR model (Fig. R15b,c). Consequently, this observation supports the M1ev-mediated delivery and subsequent accumulation of aT precisely within the vitreoretinal fibrosis microenvironment. Notably, as shown in Fig. R15b, the observation of M1ev at the retina indicates successful penetration of the internal limiting membrane (ILM).

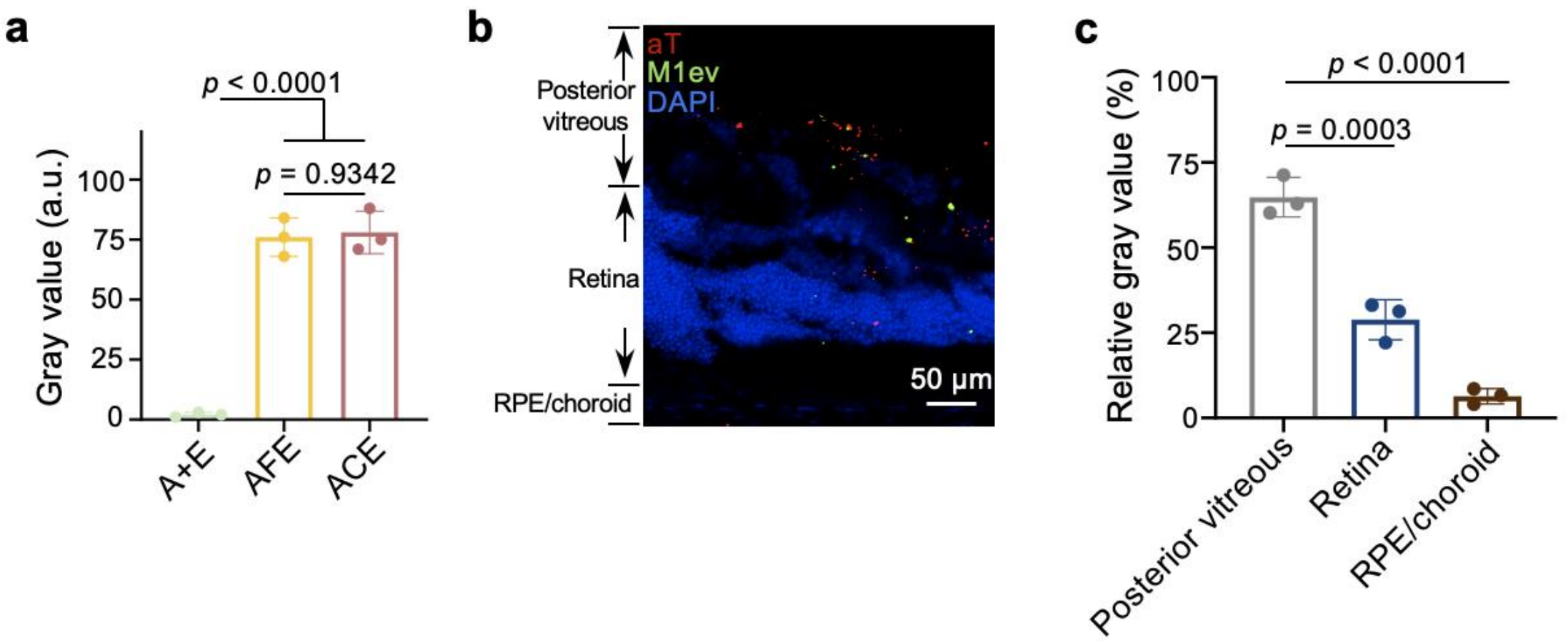

Fig. R15 (a as revised fig. S4h). Biodistribution and bioavailability of ACE.

a Quantification of aT at the lesion site (n = 3, 1 eye from each of 3 mice).

b Representative images of frozen retinal tissue sections from PVR model mice, showing localization of M1ev and aT at PVR lesions.

c Quantification of the accumulation of aT (n = 3, 1 eye from each of 3 mice).

Data in **a** and **c** are presented as mean ± s.d.. Data in **a** and **c** were compared using one-way ANOVA.

In the revised manuscript, Fig. R15a has now been added as fig. S4h, along with related figure captions. We have also provided corresponding descriptions in the main text.

Main text:

Page 6, line 214, 216: Moreover, we analyzed retina sections to investigate the release of aT following accumulation at lesions. In contrast to the pronounced overlap of aT and M1ev signals observed in AFE-treated mice and the significantly reduced aT signals in A+E group, a notable separation between aT and M1ev signals was evident in ACE group (Fig. 2h and fig. S4h). This difference should be attributed to the efficient cleavage of the linker by the elevated levels of MMP2 in PVR lesions. These results demonstrated that the ACE underwent cleavage-mediated release at PVR lesions, enabling the antibody and extracellular vesicles to function independently without interference.

Minor comments

• No description of blinding and randomization: To add per ARRIVE guidelines

Response: We thank the reviewer for pointing out this oversight. To comply with the ARRIVE guidelines, we have now added a description of blinding and randomization procedures in the methods section as follows.

Methods:

Page 14, line 569–572: All animal procedures were conducted in accordance with protocols approved by the Institutional Animal Care and Use Committee of the Institute of Process Engineering, Chinese Academy of Sciences (IPEAECA 2021112). Specifically, animals were randomly assigned to treatment groups using a computer-generated randomization sequence, and investigators performing the assessments were blinded to the group allocations until data analysis was complete.

• To clarify n numbers for each assay: Add into figure legends

Response: We appreciate the reviewer's careful review. As suggested, we have conducted a comprehensive review of all figure legends. We identified an omission in Fig. 1e, which has now been rectified by adding the specific sample size. Additionally, the n numbers for all other independent assays were verified to be correctly stated.

Main text:

Page 26, line 994-997: **e** Cell type enrichment analysis of the sequencing data via CIBERSORTx: M0 and M2 macrophages were significantly enriched in PVR samples (n = 16 PVR membranes from patients with PVR) when compared to non-PVR samples (n = 7 ILMs from non-PVR donors). Macrophage populations were determined by CIBERSORTx analysis of RNA-seq data.

This work represents an important step toward pharmacologic intervention for PVR. With the above revisions — particularly mechanistic validation, inflammatory safety assessment, and biodistribution — the study could merit publication in Nature Communications.
